# A Practical Guide to All-Atom and Coarse-Grained Molecular Dynamics Simulations Using Amber and Gromacs: A Case Study of Disulfide-Bond Impact on the Intrinsically Disordered Amyloid Beta

**DOI:** 10.3390/ijms25126698

**Published:** 2024-06-18

**Authors:** Pamela Smardz, Midhun Mohan Anila, Paweł Rogowski, Mai Suan Li, Bartosz Różycki, Pawel Krupa

**Affiliations:** Institute of Physics Polish Academy of Sciences, Al. Lotników 32/46, 02-668 Warsaw, Poland; psmardz@ifpan.edu.pl (P.S.); midhun@ifpan.edu.pl (M.M.A.); progowski@ifpan.edu.pl (P.R.); masli@ifpan.edu.pl (M.S.L.); rozycki@ifpan.edu.pl (B.R.)

**Keywords:** molecular modeling, molecular dynamics, all-atom force fields, coarse-grained force fields, Amber, SIRAH, Martini, simulation protocols, amyloid beta, intrinsically disordered proteins

## Abstract

Intrinsically disordered proteins (IDPs) pose challenges to conventional experimental techniques due to their large-scale conformational fluctuations and transient structural elements. This work presents computational methods for studying IDPs at various resolutions using the Amber and Gromacs packages with both all-atom (Amber ff19SB with the OPC water model) and coarse-grained (Martini 3 and SIRAH) approaches. The effectiveness of these methodologies is demonstrated by examining the monomeric form of amyloid-β (Aβ42), an IDP, with and without disulfide bonds at different resolutions. Our results clearly show that the addition of a disulfide bond decreases the β-content of Aβ42; however, it increases the tendency of the monomeric Aβ42 to form fibril-like conformations, explaining the various aggregation rates observed in experiments. Moreover, analysis of the monomeric Aβ42 compactness, secondary structure content, and comparison between calculated and experimental chemical shifts demonstrates that all three methods provide a reasonable choice to study IDPs; however, coarse-grained approaches may lack some atomistic details, such as secondary structure recognition, due to the simplifications used. In general, this study not only explains the role of disulfide bonds in Aβ42 but also provides a step-by-step protocol for setting up, conducting, and analyzing molecular dynamics (MD) simulations, which is adaptable for studying other biomacromolecules, including folded and disordered proteins and peptides.

## 1. Introduction

Proteins are one of the most important biological macromolecules, playing a variety of roles in organisms, such as serving as building blocks, catalyzing biochemical reactions as enzymes, facilitating molecular transport as transporters, and governing essential cellular processes as regulatory molecules [1]. For a considerable time, it was thought that every protein possessed a single unique structure, which was considered crucial for its functional role and believed to be deducible solely from its sequence (Anfinsen Dogma [2]). However, with advancements in science, it was discovered that many proteins in physiological conditions contain disordered regions or exist entirely in a disordered state [3]. Such systems are referred to as intrinsically disordered proteins (IDPs), although it may be more precise to classify them as ‘multi-structure proteins’ with low-energy conformational transitions or simply ‘intrinsically flexible systems’ [4]. This behavior is somewhat similar to the fluxional character of some molecules, which is typically used to describe small molecules that undergo rapid interconversion between structurally distinct but energetically equivalent isomers, a process also known as degenerate rearrangement [5]. However, conformations of IDPs are not energetically equivalent, and transitions between them are more complicated in nature due to the larger size of proteins. Nevertheless, due to the analogy, some works are naming the behavior of some metalo- and disordered proteins as fluxional [6,7,8].

Studying IDPs presents challenges due to their highly dynamic nature and the strong dependence of their properties on various environmental conditions, including pH, temperature, redox potential, and the presence of other molecular entities such as receptors or ligands [9]. They are also prone to oligomerization, aggregation, or nanodroplet formation [10]. One example of such a system is the monomeric form of 1–42 amyloid-β (Aβ42), a fundamental component of toxic Aβ42 oligomers [11] and fibrils associated with neurodegenerative diseases, including Alzheimer’s disease [12]. An interesting approach to expedite the study of Aβ fibril formation is the mutation of residues Leu17 and Leu34, which can be efficiently cross-linked in the fibril form, to cysteines with subsequent disulfide bond formation, forcing the peptide conformation to obtain a mature fibril structure without disturbing the fibril formation process [13]. A similar effect is also observed in other polypeptides involved in neurodegenerative diseases, such as α-synuclein, in which the addition of a disulfide bond to the C-terminus significantly increases the mechanical and thermal stability of the fibrils [14].

Disulfide bonds are the most common post-translational modification of proteins, occurring in over 20% of proteins with known structures [15]. They fulfill a myriad of roles, including structural and enzymatic stabilization, cycle regulation, and response to stress conditions, and are commonly used in industry [16]. Despite their high importance, disulfide bonds are often marginalized or even omitted in protein studies. In this work, methods for simulating the presence or absence of disulfide bonds in proteins are discussed. In addition, a novel approach allowing for dynamic formation and disruption of disulfide bonds during simulations based on finite distance restraints [17] is presented, which is most useful for studying disulfide bond breaking and reforming upon mechanical or environmental stress. It should be noted that this approach is of significantly lower resolution than ab initio MD [18,19], such as Born-Oppenheimer or second-generation Car–Parrinello MD [20] implemented in software packages such as CP2K [21] or Quantum Espresso [22], quantum mechanics/molecular mechanics (QM/MM), which can be run in Amber and Gromacs combined with the interface of CP2K or other tools, such as Quick [23], and reactive force field approaches, such as ReaxFF [24,25,26].

MD simulations have emerged as a powerful tool for investigating the structural, mechanical, and dynamical properties of macromolecules. In particular, detailed information about molecular motions at atomic resolution, as obtained from MD simulations, provides valuable insights into the conformational landscapes and dynamics of IDPs. Among the numerous options and parameters available in molecular dynamics (MD) simulations, three of them hold paramount importance and can significantly impact the results: (i) the resolution of the molecular model, (ii) the force field, and (iii) the sampling method [27]. Together, they influence how efficiently the conformational space of the studied system is explored and whether the obtained results are reliable. While most force fields for proteins have been developed over many years primarily based on well-structured molecules, the study of IDPs necessitates the consideration of methods optimized and well-benchmarked for such systems [28,29]. For instance, it has been demonstrated that older force fields, such as ff99SB [30], ff14SB [31], CHARMM22 [32], and CHARMM36 [33], yield results that more deviate from experimental data than newer parameter sets, such as ff19SB [34] and CHARMM36m [35]. Notably, parameters for elements other than proteins, particularly water [36] and ions [37], are especially significant in the case of studies involving IDPs [38]. To achieve satisfactory sampling of the conformational space, more advanced techniques, such as replica exchange MD [39] or conventional MD simulations consisting of multiple independent trajectories, each of reasonable duration, should be employed [40]. However, running simulations with more and longer trajectories can be very costly, especially for large systems comprising more than a single protein chain. Consequently, one can leverage powerful supercomputers or GPUs [41] to accelerate classical all-atom simulations or opt for simplified models, including coarse-grained ones, in which groups of atoms are represented by single interaction centers or beads. Such simplifications not only expedite the calculation of a single MD step but also permit the use of longer time steps and, by reducing the number of degrees of freedom, smooth out the energy landscape, thereby accelerating processes within the simulation [42]. However, most coarse-grained force fields are based on statistical analyses of databases and are thus less suitable for studying IDPs, which are underrepresented in the training data [43]. One approach to addressing this issue is the utilization of a physics-based coarse-grained force field, such as UNRES [44], a component of the UNICORN package [45], or coarse-grained methods developed for IDPs, such as AWSEM-IDP [46]. Nevertheless, with the development of the Martini model [47], the most popular coarse-grained force field to date [47], it has been discovered that accurate scaling of interactions between solutes and solvents can be a key factor enabling MD studies of IDPs [48]. As a matter of fact, it has been recognized that MD simulations with the Martini force field typically generate IDP conformations that are more compact than those observed in experiments. Importantly, it is possible to diminish the discrepancies between simulations and experiments by carefully scaling the protein-water interactions in the Martini force field.

In this work, we present comprehensive protocols for conducting molecular dynamics simulations of IDPs and their mutants, using the monomeric form of amyloid-β42 (Aβ42) and its L17C/L34C mutant (Aβ42_disul_) as illustrative examples. The protocol covers the application of both all-atom and coarse-grained force fields in two widely used simulation packages: Amber [49] and Gromacs [50]. For the all-atom simulations in Amber, we employ the state-of-the-art ff19SB force field [34] coupled with the OPC water model [51], which has been shown to perform well for both folded and disordered proteins and peptides [52,53,54]. We also demonstrate the use of the coarse-grained SIRAH [55] force field in Amber. In Gromacs, we showcase the application of the popular coarse-grained Martini 3 force field [47], with corrections for protein-water interactions [48] to better capture the behavior of IDPs, as well as the SIRAH force field [55]. Our protocol not only guides users through the setup and execution of these simulations but also introduces a novel approach for treating disulfide bonds dynamically, allowing them to form and break during the course of the simulation [56]. This dynamic treatment is achieved through the use of distance restraints, providing a more realistic representation of the disulfide bond behavior compared to the conventional static approach. To validate our protocol and demonstrate its utility, we present new results comparing the conformational dynamics of Aβ42 and Aβ42_disul_ using the various force fields and simulation packages. Our findings clearly illustrate the impact of the disulfide bond on the structural similarity between the monomeric form and the fibrillar state of Aβ42. Furthermore, we highlight the differences in the results obtained from the different methods employed in this study, providing valuable insights into their strengths and limitations for investigating IDPs.

## 2. Results

**Figure A1:**
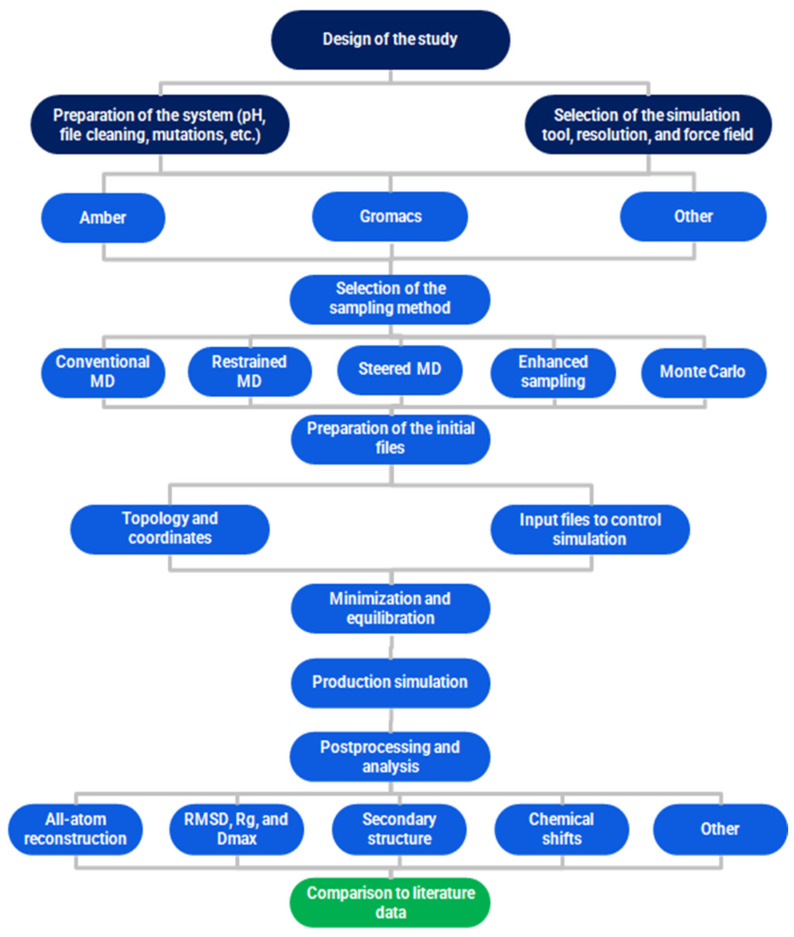
Schematic workflow for performing typical molecular dynamics simulations.

All of the results described in this manuscript are based on a specific choice of models and their parameters, all of which are described in the Section 4 and in Appendix A and Appendix B, which should provide reasonable results for IDPs and folded proteins. However, the model parameters may be easily modified by editing input files, e.g., to switch the water model, scale the water-protein interactions, or change the force field. Execution of the example scripts provided in the Supplementary Materials generates a complete workflow for preparing input files for MD simulation, performing minimization, equilibration, and production runs, as well as conducting basic analyses, as illustrated in Figure A1. Appendix C contains the technical checks needed to test the convergence of the simulations and to ensure that the periodic boundary box used in the simulations is of sufficient size to prohibit interactions between periodic images of the solute. These tests, although technical in nature, are necessary before performing standard analyses of the results. Having performed the initial verification analysis steps, as described in Appendix C, a proper analysis of the results can be conducted.

### 2.1. Structural Properties of the Monomeric Aβ42 Captured by the Force Fields

The radius of gyration (R_g_) and the maximum dimension (D_max_) are useful quantities that are independent of a reference structure and can usually be easily compared with other computational and experimental measurements. The presented results show that in the all-atom simulations with the ff19SB force field, Aβ42 tends to adopt a rather compact state with an average R_g_ of 1.29 ± 0.14 nm (Table 1). In the SIRAH coarse-grained model, Aβ42 exhibits an even more compact state, with an average R_g_ of 1.13 ± 0.11 nm. This suggests that the SIRAH force field may favor more compact conformations and underestimate the structural diversity of the peptide compared to the all-atom ff19SB force field. On the other hand, the Martini 3 force field generated the least compact conformations on average, with an average R_g_ of 1.32 ± 0.01 nm, which is not much different from the ff19SB results. This finding is further supported by the average values of D_max_, which are the highest for Amber ff19SB, slightly higher than for Martini 3, and much lower for SIRAH (Table 1). This indicates that Martini 3 allows Aβ42 to explore a wider range of conformations, thus better capturing the intrinsically disordered nature of the monomeric peptide.

**Figure 1:**
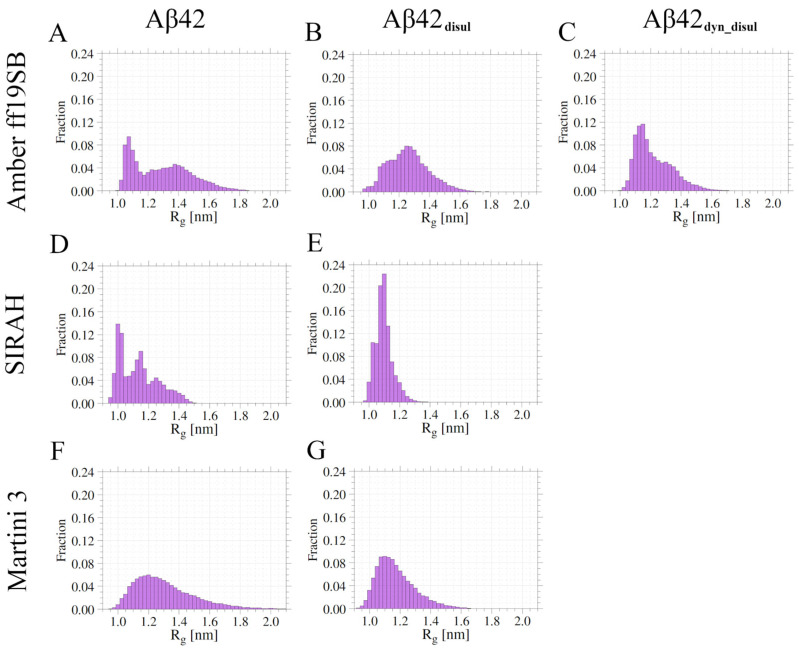
Histograms of R_g_ values obtained from the simulations in Amber ff19SB (panels **A**–**C**), SIRAH (panels **D**,**E**), and Martini 3 (panels **F**,**G**) force fields for wild-type (panels **A**,**D**,**F**) and disulfide-bond mutants (panels **B**,**C**,**E**,**G**).

The analysis of R_g_ histograms (Figure 1) provides insights into the conformational preferences of monomeric Aβ42 in the different force fields. In the Martini 3 force field, a broad single-peak distribution is observed, indicating that monomeric Aβ42 explores a wide range of conformations without strong preferences for specific structures. This suggests that Martini 3 captures well the intrinsically disordered nature of monomeric Aβ42. In contrast, the R_g_ histograms obtained from the simulations with the Amber ff19SB and SIRAH force fields exhibit multi-peak behavior. This indicates that in these force fields, monomeric Aβ42 has a tendency to form semi-stable conformations, sampling distinct structural states. The presence of multiple peaks suggests that certain conformations are energetically favored and more frequently visited during the simulations. Notably, the main peak of the R_g_ distribution in SIRAH is observed at a very low R_g_ value of around 1.0 nm, and the overall R_g_ distributions span the smallest range among the force fields studied. This finding implies that the SIRAH force field may not fully capture the disordered character of monomeric Aβ42, as it appears to favor more compact conformations and underestimate the structural diversity of the peptide. It should be noted that due to the coarse-grained representation and longer computational time, the Martini simulations yield much more rapid and frequent jumps of both R_g_ and D_max_ than the all-atom simulations (see Appendix C); however, the average values are very similar in these methods, with only SIRAH generating more compact conformations (Table 1).

**Figure 2:**
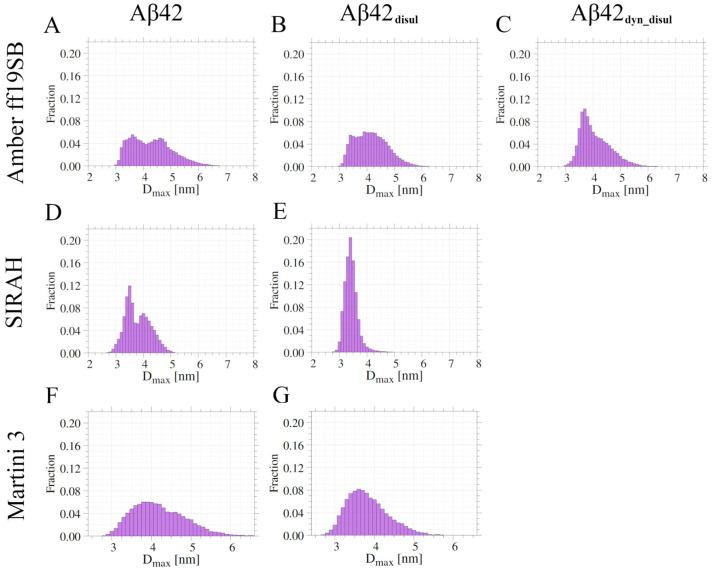
Histograms of D_max_ values obtained from the simulations in Amber ff19SB (panels **A**–**C**), SIRAH (panels **D**,**E**), and Martini 3 (panels **F**,**G**) force fields for wild-type (panels **A**,**D**,**F**) and disulfide-bond mutants (panels **B**,**C**,**E**,**G**).

The distributions of maximum dimension (D_max_) obtained from the simulations (Figure 2) exhibit similar trends to the R_g_ distributions, but are generally more uniform. While the R_g_ distributions obtained from the Amber ff19SB and SIRAH force fields show multiple peaks, indicating semi-stable conformations, the D_max_ distributions are generally smoother. The presence of multiple peaks in the R_g_ distributions suggests energetically favored conformations that are more frequently sampled, while the smoother D_max_ distributions highlight the inherent flexibility of the monomeric Aβ42, with significant portions of the peptide remaining highly dynamic and disordered.

Analysis of the solvent accessible surface area (SASA) shows that the highest SASA values are obtained for the Martini force field, while the lowest values are observed for SIRAH. Interestingly, the Amber ff19SB force field generated the average SASA values much closer to those from SIRAH than from Martini. This finding is noteworthy as it does not fully correlate with the compactness of the monomeric Aβ42 captured by the R_g_ and D_max_ values and demonstrates a higher solubility of the Aβ42 in the Martini 3 than in the other force fields.

**Figure 3:**
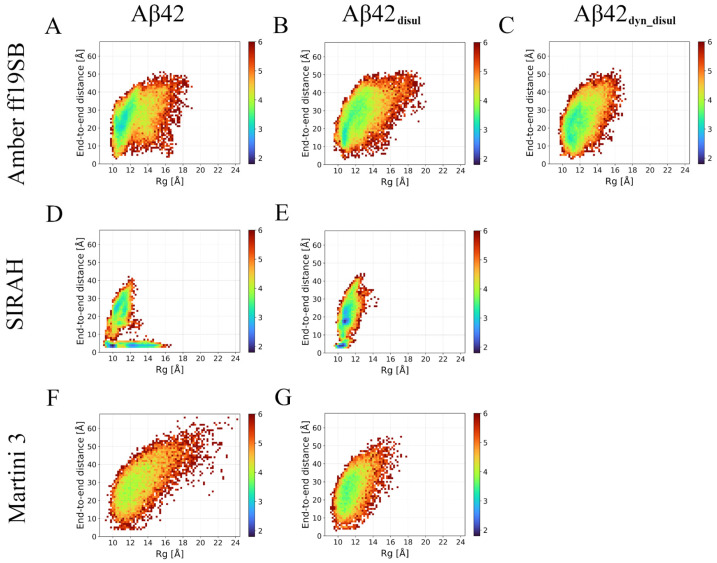
Energy landscapes calculated based on the end-to-end distance and R_g_ values in all trajectories in Amber ff19SB (panels **A**–**C**), SIRAH (panels **D**,**E**), and Martini 3 (panels **F**,**G**) force fields for wild-type (panels **A**,**D**,**F**) and disulfide-bond mutants (panels **B**,**C**,**E**,**G**).

Energy landscapes can provide valuable insights into how well each force field captures the intrinsically disordered nature of the monomeric Aβ42. Analysis of the R_g_ versus end-to-end distance maps (Figure 3) shows that the Martini 3 force field exhibits a very shallow and broad energy minimum, corresponding to an ensemble of conformations with many quite extended structures. This suggests that Martini effectively represents the highly flexible and disordered character of the monomeric Aβ42, allowing it to explore a wide range of conformations without strong preferences for specific structures. The Amber ff19SB force field shows similar behavior to Martini, but with a slightly deeper and narrower energy minimum. This indicates the presence of some semi-stable conformations in the simulations, implying that Amber ff19SB captures the disordered nature of Aβ42 to a slightly lesser extent compared to Martini. However, the energy landscape is still relatively broad, suggesting that Amber ff19SB allows for significant conformational flexibility. In contrast, the SIRAH force field displays a rather narrow energy landscape with multiple deep energy minima. This indicates a strong tendency for the monomeric Aβ42 to form specific conformations, particularly those where the N- and C-termini are in close proximity. Thus, SIRAH may not fully capture the intrinsically disordered character of the monomeric Aβ42. However, it should be noted that in Martini there is also a population of conformations, in which there are interactions between the termini; however, they are much less frequent. Overall, the energy landscape analysis provides further evidence that the Martini force field most accurately represents the disordered nature of monomeric Aβ42, followed by Amber ff19SB, while SIRAH appears to have limitations in capturing the full extent of the Aβ42 intrinsic disorder.

**Figure 4:**
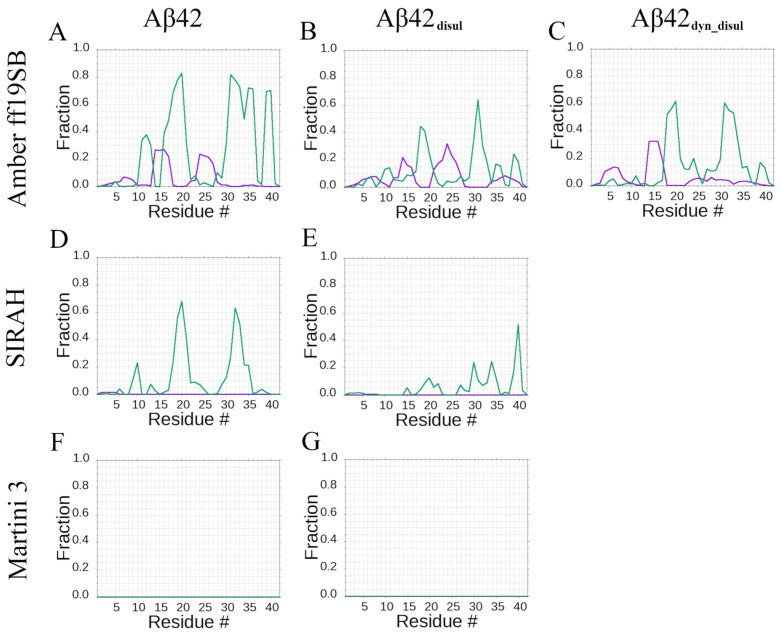
Propensity for amino-acid residues to form β-strands (green) or α-helices (violet), averaged over simulation time and trajectories in Amber ff19SB (panels **A**–**C**), SIRAH (panels **D**,**E**), and Martini 3 (panels **F**,**G**) force fields for wild-type (panels **A**,**D**,**F**) and disulfide-bond mutants (panels **B**,**C**,**E**,**G**).

Distributions of the secondary structure content per residue show that both the all-atom Amber ff19SB and coarse-grained SIRAH force fields predict the same amino acid residues to have a preference for forming β-strands: residues 16–21 and 30–35 (Figure 4). However, this propensity is higher in the all-atom Amber ff19SB simulations and covers slightly wider ranges, including residues 30–37 and additionally 38–40. Quantitatively, the Amber ff19SB force field predicts β-strand content of approximately 60–80% for residues 16–21 and 30–40, while SIRAH yields around 40–60% β-strand content for residues 16–21 and 30–35. These results indicate that the all-atom Amber ff19SB force field captures a stronger tendency for these regions to adopt β-strand conformations compared to the coarse-grained SIRAH model. The tendency to form α-helices by the monomeric Aβ42 is noticeable only in the all-atom Amber ff19SB simulations, mainly for residues 13–17 and 23–27, with the α-helical propensities reaching around 20–25%. In contrast, the coarse-grained SIRAH force field does not predict any significant α-helical content for the monomeric Aβ42, while in Martini 3 simulations, no secondary structure content is observed.

### 2.2. Impact of Solute-Solvent Interaction Scaling on the Monomeric Aβ42 in Martini 3 Force Field

**Figure 5:**
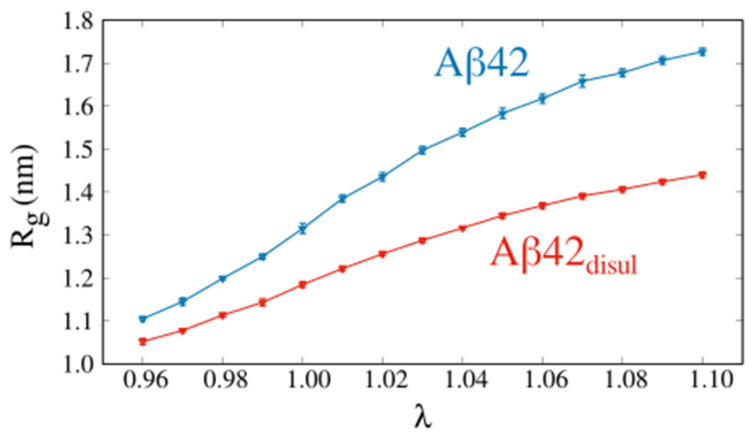
Average radius of gyration, R_g_, as a function of the water-protein interaction rescaling parameter λ obtained from the Martini simulations of Aβ42 (blue) and Aβ42_disul_ (red). The error bars indicate the standard deviation calculated over a single trajectory using block averaging.

In this work, we also present a novel approach to scale interactions between solute and solvent in the Martini force field, which significantly impacts the compactness of the Aβ42 (Figure 5). As one would expect, the average R_g_ increases monotonically with increasing the water-protein interaction rescaling parameter, λ, both in the case of Aβ42 and Aβ42_disul_. Indeed, enhancing the protein-water interactions should result in expanded conformations of IDPs [48,57], but this has not been tested so far for the monomeric Aβ42. Importantly, by comparing the average R_g_ from the simulations with the R_g_ from the experiments, one can determine the optimal value of parameter λ that yields the best agreement between the simulation and experiment [48].

**Figure 6:**
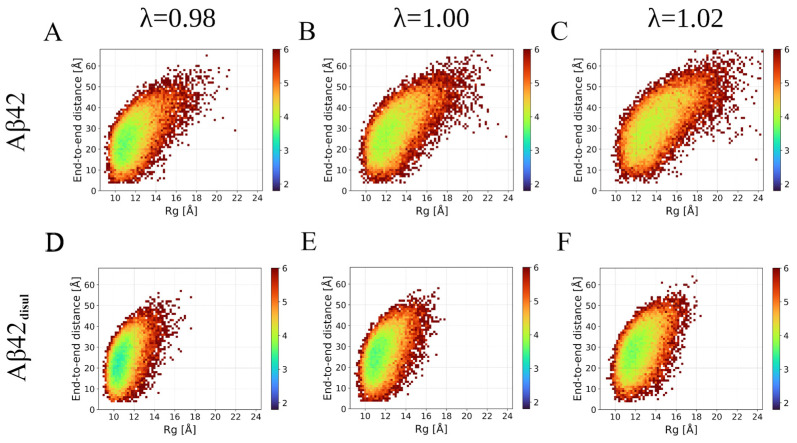
Energy landscapes based on the end-to-end distance and R_g_ distributions obtained from the Martini 3 simulations of Aβ42 (upper panels: **A**–**C**) and Aβ42_disul_ (lower panels **D**,**F**) with the solute-solvent scaling parameter λ set to 0.98 (left panels: **A**,**D**), 1.00 (center panels: **B**,**E**), and 1.02 (right panels: **C**,**F**).

Distributions of the end-to-end distance and R_g_ (Figure 6) clearly show that relatively small changes in the solute-solvent interactions, corresponding to a 2% reduction or increase in λ, do not significantly alter the overall behavior of the system. However, even these small variations in λ can result in a shift in the compactness of the structure by approximately 10% (Figure 5 and Figure 6, Table 2). Therefore, applying a small solute-solvent scaling may be safely used to achieve better agreement with experimental data while preserving the overall behavior of the system. This approach allows one to fine-tune the Martini 3 force field to more accurately reproduce experimental observables related to the compactness of such IDPs as the monomeric Aβ42, while still preserving the key characteristics and dynamics of the system.

### 2.3. Impact of the Disulfide Bond Addition to the Monomeric Aβ42

The L17C/L34C mutant of Aβ42 with an engineered disulfide bond (Aβ42_disul_) is an experimental construct that facilitates studies of the initial aggregation steps of Aβ42 without significantly altering the fibril morphology. Our simulations reveal that Aβ42_disul_ adopts more compact structures compared to the wild-type Aβ42 (Table 1) across all force fields tested, with this effect being most pronounced in the Martini force field. Larger differences are observed in D_max_, indicating that while the overall compactness is not significantly altered, the presence of the disulfide bond prevents Aβ42_disul_ from adopting the most extended conformations. Interestingly, despite the tendency of Aβ42_disul_ to form more compact structures, it exhibits slightly higher solubility in the Amber ff19SB and SIRAH force fields, as evidenced by the increased SASA values. This trend is less apparent in the simulations with the Martini 3 force field, where the more substantial decrease in R_g_ has a greater impact on the SASA. However, the decrease in SASA is proportionally smaller than the decrease in R_g_ (7.8% versus 10.0%), suggesting that a similar solubility-enhancing effect is still present.

**Figure 7:**
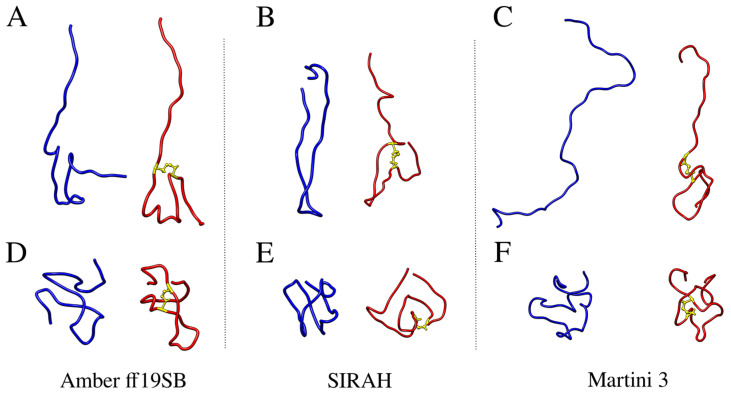
Ribbon representation of the structures from the MD simulations with the all-atom Amber ff19SB (**A**,**D**), coarse-grained SIRAH (**B**,**E**) and coarse-grained Martini 3 (**C**,**F**) force fields with the maximum (**A**–**C**) and minimum (**D**–**F**) R_g_ values shown in blue and red, respectively. Disulfide bonds are marked as yellow sticks.

Only small differences between average R_g_ values with and without the disulfide bond are observed for the Amber ff19SB and SIRAH force fields. This is contrary to Martini, in which the presence of a disulfide bond decreased the average radius of gyration by about 10%. This effect is caused by the propensity of Aβ42 to form β-hairpins by central and C-terminal amino-acid residues, thereby stabilizing the interactions in which the disulfide bond is being added in the first two force fields, which is not observed in Martini (Figure 7 and Table 3). Visualization of the most extended and most compact structures obtained from the all-atom and coarse-grained simulations with and without the disulfide bond clearly shows that Aβ42 can unfold almost completely in Martini when the disulfide bond is not present, while in Amber ff19SB and SIRAH, some stabilizing interactions are always present. Introduction of the disulfide bond prevents the complete unfolding of monomeric Aβ42 in Martini, while it does not significantly impact the conformation of the peptide in Amber ff19SB and SIRAH. What is a visualization of the impact of the disulfide bond on the R_g_ values (Table 1 and Figure 1).

**Figure 8:**
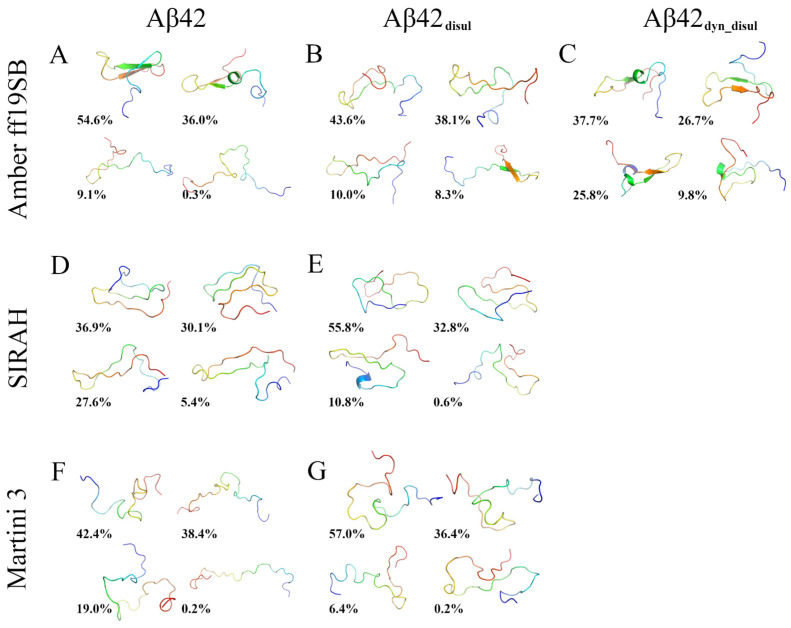
Cartoon representation of the most abundant Aβ42 conformations determined from the clustering analysis in Amber ff19SB (panels **A**–**C**), SIRAH (panels **D**,**E**), and Martini 3 (panels **F**,**G**) force fields for wild-type (panels **A**,**D**,**F**) and disulfide-bond mutants (panels **B**,**C**,**E**,**G**). The population percentages are given below the snapshots. The peptide structure is rainbow colored from blue (N-terminus) to red (C-terminus).

A hierarchical agglomerative clustering was performed to identify the most abundant conformations of the monomeric Aβ42 sampled during the simulations using the different force fields (Figure 8). Then similarities and differences between these most populated conformations (cluster centroids) were assessed by calculating the root-mean-square deviation (RMSD) of atomic positions (Table S1). The RMSD analysis reveals that each simulation method generated a diverse set of structures. Some of these structures are found to be relatively similar to those obtained by the other methods, with pairwise RMSD values ranging from 4.7 to 21.1 Å. However, the conformations sampled in the SIRAH simulations exhibit the greatest differences compared to those obtained by the other methods. This suggests that SIRAH may explore a somewhat different region of conformational space compared to the Amber ff19SB and Martini 3 force fields. This is also evident on the energy landscapes (Figure 3), where only in SIRAH there is a large group of conformations, in which the N- and C-termini are very close to each other forming stable interactions. Interestingly, the addition of the disulfide bond in the SIRAH simulations alters the sampled conformations, bringing them much closer to those obtained by the other methods, with fewer conformations comprising contacts between the N- and C- termini. This indicates that the presence of the disulfide bond constrains the conformational flexibility of Aβ42 in the SIRAH model, leading to structures that are more similar to those observed in the Amber ff19SB and Martini 3 simulations. In general, populations of conformations, as characterized by the compactness properties (histograms of R_g_ and D_max_ in Figure 1 and Figure 2, respectively), indicates that the addition of the disulfide bond alters the conformational ensemble towards unimodal distributions, which is especially pronounced in cases of Amber ff19SB and SIRAH, while for the Martini 3 the most important change is the shift towards lower values (or more compact structures).

The analysis of cluster representatives shows that only the all-atom Amber ff19SB force field allows the monomeric Aβ42 to adopt stable secondary structure elements such as β-strands and α-helices (Figure 8). In contrast, such well-defined structures are not present in the representative structures obtained from the coarse-grained SIRAH and Martini simulations. While the SIRAH models do contain structures with some resemblance to β-strands, these secondary structure elements appear distorted and are not recognized by standard secondary structure assignment algorithms such as DSSP [58] or those implemented in PyMol [59] (see Section 3 for more details). The addition of the disulfide bond has the most pronounced effect on the monomeric Aβ42 in Martini, in which none of the cluster-representative structures are almost completely unfolded, contrary to the simulation of the wild-type peptide, where the least abundant ensemble (0.2% of the total population) is almost completely extended.

**Figure 9:**
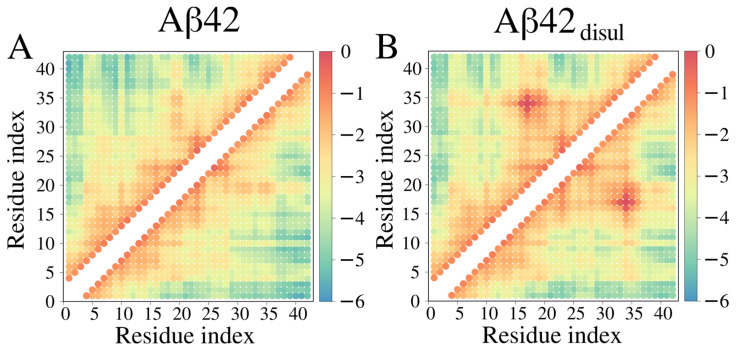
Contact maps obtained from coarse-grained structures of Aβ42 (**A**) and Aβ42_disul_ (**B**) generated in the Martini simulations with λ = 1 (no scaling of solute-solvent interactions). The color scale indicates the natural logarithm of contact frequency. The points in red, yellow, and blue represent frequent, transient, and rare contacts, respectively.

The presence of the disulfide bond between Cys17 and Cys34 is clearly reflected in the contact map (Figure 9), which shows an increased frequency of contacts in the region surrounding these two residues. Additionally, a small increase in contact frequency is observed between residues 4–8 and 33–37 in Aβ42_disul_, suggesting that the disulfide bond promotes some longer-range interactions between the N- and C-terminal regions of the peptide. In general, the contact map analysis reveals that the introduction of the Cys17-Cys34 disulfide bond results in a notable increase in the number of stabilizing intramolecular interactions in Aβ42 when simulated using the Martini 3 force field.

Another interesting property of the Aβ42 is its secondary structure content (Table 4), which can be compared to the experimental findings (see Section 3 for more details). Interestingly, the addition of a disulfide bond slightly decreased the β-content of Aβ42. Examination of the secondary structure content (Table 4) showed that the Martini simulations failed to predict any semistable secondary structures. A similar effect, but to a smaller extent, was observed for the SIRAH force field. This may be caused by imperfections arising from all-atom reconstruction and secondary structure prediction based mostly on hydrogen bonds, which is, however, the most popular method for determining secondary structures based on PDB files [58].

The incorporation of the disulfide bond decreases the propensity of Aβ42_disul_ to form β-strands by approximately half in the simulations with the Amber ff19SB and SIRAH force fields. However, it does not change the specific regions exhibiting the β-strand propensity. Notably, the disulfide bond reduces the β-strand content in residues 30–35 from around 60–80% to 30–40% in Amber ff19SB and from 40–60% to 20–30% in SIRAH.

Since the monomeric Aβ42 is intrinsically disordered and lacks a stable structure for reference in RMSD calculations, RMSD plots of the initial structure are not presented here. Nevertheless, RMSD for the initial or average structure can be easily calculated with minor modifications of the analysis scripts. On the other hand, we examined how similar the conformations during simulation are to those in fibrils by measuring the RMSD for seven fibril models (U-(2BEG, 2M4J, and 2LMN), S-(5KK3, 2MXU, 2NAO), and LS-(5OQV) shape) deposited in the PDB database (Table 5). Analysis revealed that there is a significant increase in the similarity to fibrils when a disulfide bond is present in the all-atom Amber ff19SB force field. An even larger increase can be observed in the SIRAH force field. On the other hand, results from Martini show that there is a very low similarity of the obtained structure to fibrils, and the addition of a disulfide bond only slightly increases it. This is partially caused by the very large flexibility of the polypeptide chain in the Martini force field, observed in large R_g_ fluctuations, and the ability to temporarily obtain conformations resembling various types of fibrils (Table 6).

## 3. Discussion

In this work, we demonstrated how all-atom and coarse-grained force fields can be utilized to study a model IDP—monomeric Aβ42. To ensure the high reliability of the results, state-of-the-art methods were employed. Specifically, the all-atom Amber ff19SB force field for proteins was used, coupled with the four-point OPC water model [51] and a co-optimized ions model [37] to mimic physiological salt concentration, approximately 0.15 M NaCl [60]. Additionally, the SIRAH coarse-grained force field within the Amber and Gromacs packages and the Martini 3 coarse-grained model and force field within the Gromacs package were employed. In the latter approach, solute-solvent interactions were scaled up and down, and the results were compared to the radius of gyration values to ensure the correct compactness of the simulation structures. The use of a coarse-grained representation significantly accelerated the simulations by approximately 1–2 orders of magnitude, with no significant impact on the results. However, it is worth noting that the use of coarse-grained models can complicate the analysis, as it often requires reconstruction to all-atom models, and some atomistic details are lost due to simplifications of the protein representation. Still, coarse-grained models seem to be a method of choice when it comes to extensive simulations of large IDP complexes [61] and biomolecular condensates [62,63].

Overall, the results obtained indicate that all the methods produced largely unstructured conformations, as expected for the monomeric Aβ42. However, this outcome is not always achieved, especially when older methods are employed [27,64]. Due to the much larger computational cost associated with the all-atom simulations, only three trajectories, each of 2 ms, were executed, which may not provide the highest level of robustness in the results. In contrast, with the coarse-grained SIRAH and Martini methods, simulations were at least 10 times longer, and convergence was reached more quickly due to the reduction in the number of degrees of freedom, resulting in a much lower total computational cost. This approach of employing two entirely different computational methods, such as all-atom and coarse-grained force fields, is recommended for obtaining highly reliable results, especially in the absence of or with limited experimental data.

Earlier computational studies suggest that R_g_ values for Aβ42 are in the range of 0.8 to 1.2 nm [65,66,67], while the experimentally lowest found R_g_ values are about 1 nm, which should correspond to the most compact conformations of the monomers. However, it should be noted that the experimentally observed hydrodynamic radius strongly depends on the conditions and is found to be in the range of 0.8 to 1.5 nm [67], further confirming the molecular flexibility of Aβ42. More recent studies still present very different R_g_ values, ranging from 1.0 [68] to 1.6 nm [69], obtained from SANS experiments. In the context of this evidence, all of the calculated values in this work fit into experimental and theoretical R_g_ ranges, however, all-atom Amber ff19SB and Martini 3 better sample the extended conformations.

The observed average SASA values for the Amber ff19SB force field coupled with the OPC water model of about 38 nm^2^ are significantly larger than the previously obtained SASA values of 32–33 nm^2^ for other Amber force fields coupled with the TIP3P model, while the SASA of about 43 nm^2^ is very close to the results obtained in CHARMM36m, which yielded SASA = 42 nm^2^ [27]. This may also explain why (although Martini shows comparable R_g_ and D_max_ values to Amber ff19SB) the observed SASA values are greater in Martini, as the CHARMM36 force field was used for the backmapping and may contribute to a larger exposure of side chains to solvent.

Analyzing not only average properties but also distributions of their values in the form of histograms and conformational maps provides valuable insights into the structural preferences and dynamic behavior of the system [10,70]. Histograms of such properties as the R_g_ and D_max_ reveal whether the system exhibits a tendency to form semi-stable conformations or explores a wide range of conformations with no strong preferences for specific structures. Energy landscapes, calculated based on the distributions of the end-to-end distance and R_g_, provide information on the relative stability of different conformational states and the energy barriers between them. These analyses are particularly important for IDPs such as the monomeric Aβ42, as they capture the inherent flexibility and conformational diversity of these systems, which may not be adequately represented by average values alone.

Although this manuscript describes most of the standard analyses used for typical studies involving proteins or peptides, more advanced techniques can be employed to gain deeper mechanistic insights, especially when studying complex systems. For example, normal mode analysis (NMA) [71] or essential dynamics [72] can extract the dominant motions from MD trajectories, revealing functionally relevant conformational changes. Markov state models (MSMs) built from MD simulations [73] provide a powerful framework for analyzing the conformational dynamics of proteins, allowing for the identification of metastable states and the kinetics of transitioning between them. This approach is particularly useful for systems exhibiting long timescale processes such as protein folding or oligomerization. Graph-based visualizations based on transition network analysis methods [73,74] can also yield valuable information about the free energy landscape by representing the conformational space as a network, with nodes representing distinct conformational microstates and edges representing the transitions between them. These advanced techniques would be especially beneficial when investigating more complex systems, such as Aβ42 oligomers [75]. The energy landscape in the form of conformational maps of Aβ42 tetramers and higher-order oligomers is highly intricate, featuring multiple association-dissociation events and conformational rearrangements that are challenging to fully capture using standard analyses alone. Integrating NMA, MSMs, and graph-based approaches with conventional methods would provide a more comprehensive understanding of the dynamical behavior and mechanisms underlying the formation and interconversion of these oligomeric species, which are crucial in the pathogenesis of Alzheimer’s disease. However, in the case of the monomeric Aβ42, simple energy landscapes should be sufficient to represent the disordered character of this peptide. This is especially true for the Martini 3 simulations, in which no semi-stable structures are observed. On the other hand, some stable conformations are noticeable in the SIRAH simulations without the disulfide bond; however, they are likely an effect of the Aβ42 overstabilization.

Due to the disordered character of monomeric Aβ42 in water, we cannot compare it to experimental structures in similar conditions. Although the monomeric Aβ42 is disordered in water solution, it can attain relatively stable conformations in apolar environments [76]. As a matter of fact, structures of the monomeric Aβ42 in such conditions can be found in the PDB (see, e.g., entries with the deposition codes 1IYT, 1Z0Q, and 6SZF) [76,77,78]. However, their properties and conformations are significantly different from those in water and cannot be directly compared. For an aqueous environment, only structures of Aβ42 (proto)fibrils are available so far.

To test how accurate the results obtained in this study are compared to the experimental results, we calculated correlations, differences (ΔH), and root-mean-square-errors (RMSE) between chemical shifts predicted for PDB structures from the trajectories and the experimental values [79] (Table 7 and Table 8). This analysis shows that all of the methods agree well with the experiment, with an especially high correlation observed for the Martini simulations without solute-solvent scaling. Not surprisingly, the cysteine mutant with a disulfide bond shows worse agreement with the experimental data than the wild-type Aβ42 peptide; however, the difference is not very large, indicating that this mutation does not significantly alter the properties of the molecule. In general, Amber ff19SB coupled with the OPC water model obtained similar results to the previously tested ff14SB with the TIP3P water model, yet worse than CHARMM36m [27]. It should be noted that these results are significantly better than those obtained using older force fields, such as Amber ff99SB [27] or variants of CHARMM22 [80,81]. According to previous studies, these results could probably be further improved by extending the trajectories even further [82] or using enhanced sampling methods, such as replica exchange molecular dynamics simulations [27].

It should be noted that although chemical shifts provide a measure that can be used for benchmarking force fields for IDPs, such as monomeric amyloid β [80], the correlation between computational and experimental values of the chemical shifts for carbon atoms is almost always higher than for hydrogen and nitrogen atoms, which are encumbered by higher relative errors [83]. While it is understandable for hydrogens, as they are most mobile and therefore often stiffened during simulations to allow the use of a large timestep, or poorly reconstructed from coarse-grained representations, this is not the case for nitrogen atoms, which tend to have too low ΔH values in many all-atom force fields [84], independent of the method used for the chemical shift prediction.

Our results support these previous observations, showing that despite using much different and newer force fields, the chemical shifts for nitrogens are underestimated (Table 8). A direct comparison of the chemical shift values shows that SIRAH, and not Martini, obtained values closest to the experimental ones; however, these values are not as highly correlated with the experimental data as those obtained using the Amber ff19SB force field (Table 7 and Table 8). This observation suggests that while SIRAH may reproduce the magnitude of nitrogen chemical shifts more accurately, the Amber ff19SB and Martini 3 force fields capture the relative variations between residues better, as reflected by the higher correlation coefficients.

**Table 7:** Correlations between chemical shifts calculated by Shifts 5.6 [85] and those determined experimentally. The correlation coefficients are averaged over trajectories. Standard deviations are given in brackets.

Force Field	Variant	Cα	Cβ	N
Amber ff19SB	wt	0.982 (0.007)	0.990 (0.004)	0.838 (0.036)
	CC mutant static	0.984 (0.003)	0.978 (0.004)	0.813 (0.029)
	CC mutant dynamic	0.981 (0.006)	0.978 (0.001)	0.864 (0.038)
SIRAH	wt	0.981 (0.001)	0.991 (0.002)	0.780 (0.023)
	CC mutant	0.972 (0.004)	0.979 (0.003)	0.806 (0.037)
Martini 3	wt	0.992	0.995	0.896
	CC mutant	0.990	0.979	0.853

**Table 8:** Difference (ΔH = H_experimental_ − H_computational_) [ppm] and RMSE [ppm] between chemical shifts calculated by Shifts 5.6 [85] and those determined experimentally for the wild-type monomeric Aβ42.

Force Field	Cα		Cβ		N	
	ΔH	RMSE	ΔH	RMSE	ΔH	RMSE
Amber ff19SB	0.22	1.06	−0.58	1.52	−2.17	4.03
SIRAH	0.03	0.67	0.18	0.96	−1.01	2.60
Martini 3	0.36	0.75	0.23	0.93	−6.29	6.65

The average secondary structure content obtained using Amber ff19SB and SIRAH aligns well with previous experimental findings, which have established the α-helical content to be within the 3–9% range and the β-content to be 12–25% [86]. These values are also similar to those obtained in our previous studies using the CHARMM36m force field [27]. Distributions of the secondary structure content per residue show good agreement with previous theoretical studies (Figure 4). Teplow et al. have observed the α-helical propensity for residues 26–31 and the β-strand propensity for residues 19, 29–33, and 37–39 [87]. Similarly, Strodel et al. have found the α-helical propensity for residues 11–19, 22–26, and the β-strand propensity for residues 16–21 and 30–40 across various force fields [80]. The ranges for the α-helical propensity obtained in this study are very similar to those previously reported for Amber ff14SB using the same sampling methods [27]. However, the β-strand propensity observed here more closely resembles the results from CHARMM36m and ff14SB obtained using replica exchange molecular dynamics (REMD)-enhanced sampling. This suggests that while the ff14SB force field coupled with the TIP3P water model may overstabilize certain conformations, requiring enhanced sampling to accurately capture structural elements and their transitions, the Amber ff19SB force field exhibits proper behavior even with classical MD sampling. The improved performance of ff19SB likely stems from its optimized parameters and the use of the OPC water model, which better represent the balance between protein-protein and protein-water interactions crucial for accurately simulating intrinsically disordered systems such as the monomeric Aβ42. In summary, the secondary structure propensities obtained using Amber ff19SB and SIRAH in this study are consistent with both experimental data and previous computational studies. The results highlight the importance of force field selection and sampling methods in accurately capturing the conformational ensemble of IDPs such as Aβ42.

It should be noted that the presence of distorted secondary structure elements (Figure 8) is not unexpected in coarse-grained simulations because the simplified representation of amino-acid residues, coupled with the use of standard force fields (with no terms for secondary structure corrections), can lead to inaccuracies in local protein structures. This is a commonly known limitation of coarse-graining. Moreover, the procedure used to reconstruct all-atom chains from coarse-grained models is another potential source of structural imperfections. The backmapping from coarse-grained to all-atom representations is a challenging problem and can introduce artifacts [42]. The structural imperfections from backmapping could potentially be alleviated by applying more sophisticated reconstruction procedures, similar to the one employed in Martini 3, which involves a series of short MD simulations with restraints using an all-atom force field such as CHARMM36, rather than relying on the standard backmapping tool such as the one provided by the SIRAH developers. Such restrained MD simulations allow for the reconstructed all-atom model to relax and adopt a more realistic local geometry while still maintaining the overall tertiary structure captured by the coarse-grained simulation. However, this approach may transfer some local tendencies arising from the used all-atom force field, such as the increased SASA values in the case of CHARMM36 and 36m force fields (Table 1).

Point mutations are commonly introduced to proteins and peptides to either decrease or increase their aggregation properties, depending on the study’s objectives. In the case of Aβ42, a series of MD simulations were conducted on the L17C/L34C mutant with a disulfide bond acting as a cross-link between the amino-acid residues that come into contact in the fibrillar form. The designed mutant is known to efficiently form fibrils with both the wild-type and mutated Aβ42, emphasizing some of the fibril-like contacts, including Q15-V36, L17-L34, and F19-I32 [13]. Interestingly, our all-atom and coarse-grained simulations, even without the disulfide bond, exhibit a significant number of conformations forming the latter two contacts, and these conformations were further stabilized when a disulfide bond was incorporated (see Figure 6 and Table 3). We also observed many contacts between the central hydrophobic core (residues 16–22) and the C-terminal region (residues 30–42), indicating the formation of β-hairpins, which were previously shown to be important for the oligomerization of Aβ42 and its variants [88,89]. Surprisingly, despite the higher similarity of the monomeric Aβ42 conformations to the fibrils for the disulfide-bond variant, a decrease in β-content is observed in the Amber ff19SB and SIRAH force fields. The higher propensity of Aβ42 to form fibril-like conformations when a disulfide bond is present seems to be a major reason for the experimentally observed speed-up in aggregation without wild-type fibril seeding in Aβ40 [13], which is consistent with the N* theory that connects the number of fibril-like conformations in the monomeric state with the aggregation rate [90]. This effect, in this case, cannot be simplified to an examination of the impact of β-content on the aggregation rate, which, on the other hand, is probably responsible for the decrease of the aggregation rate when wild-type fibril seeding is used in Aβ40 [13]. These effects also suggest that although the disulfide-bonded L17C/L34C variant can form fibrils with wild-type Aβ, the morphology of these fibrils is not identical, which is typical for disulfide-bond modifications in other amyloids [91,92]. It should be noted that other mutations involving disulfide-bond cross-linking in Aβ may have opposite effects. For example, the introduction of the Cys21-Cys30 disulfide bond prohibited the formation of fibrils; however, it increased the formation of toxic oligomers [93].

The use of the coarse-grained SIRAH model enabled us to obtain approximately a 6-fold decrease in the computational time needed to perform 1,000,000 steps compared to the all-atom ff19SB force field (Table 9). Taking into account a 10 times longer timestep value, this provided a 60-fold speed-up when using a low-end GPU, while the total number of interaction centers decreased by about 13 times. These values are in agreement with those obtained by SIRAH developers for a much larger system [55]. However, it should be noted that in the case of the monomeric Aβ42, the SIRAH simulations on a GPU did not scale at all, even with the use of a much more powerful GPU, while for the all-atom system, a 4-fold speed-up was observed, which most likely was caused by the small size of the studied system. An 80-fold speed-up was observed in the Amber all-atom force field when using a state-of-the-art GPU instead of a 20-core CPU node, whereas this difference was only about 10-fold if SIRAH is used in the Amber package. Interestingly, the SIRAH simulations in Gromacs are significantly faster than the SIRAH simulations in Amber when the CPU is used. This difference may be, at least partially, explained by the use of a simpler cutoff for the Van der Waals interactions in Gromacs (simple cutoff) than in Amber (Particle Mesh Ewald—PME). The dissimilarity disappears when the same GPU is used with PME treatment for all long-range interactions. While this observation may suggest that the CPU performance of Gromacs is superior to that of Amber, further investigations are needed to fully assess the impact of a simpler cutoff approach and to examine various systems on different hardware. It should be noted that the use of the Martini 3 model in the Gromacs package provides about a 15-fold speed-up compared to SIRAH in the Amber package when a similar CPU is used, which most likely comes from a much different coarse-grained model used (especially for water), different cutoff methods, and different ranges. Moreover, the performance of Martini in Gromacs scales well even when a high number of CPU cores is used (e.g., three times speed-up when 64 cores are used instead of 12). It should be noted that the Gromacs package [50] is versatile in the sense that it can be used to perform MD simulations not only with such coarse-grained models as Martini [47] and SIRAH [55], but also within the all-atom model with various force fields, including GROMOS [94], CHARMM [95], and AMBER [34]. Similarly, the Amber package can be used with other than Amber force fields, including CHARMM for proteins and lipids and GAFF [96] for small organic molecules.

In general, all of the tested force fields provided reasonable results in terms of the data that can be experimentally compared, such as the radius of gyration and chemical shifts. SIRAH tends to generate more compact conformations, and fully extended conformations are not observed in it, which may suggest that the method overstabilizes the occurrence of some contacts; however, the obtained properties are still of reasonable quality. On the other hand, Martini tends to generate highly fluctuating conformations within relatively short timescales, and almost no semistable secondary structure elements are observed during the simulations. This effect is probably caused by the fact that for folded proteins, an elastic network coupled with secondary structure parameters is generated in the initial steps of Martini 3, which is necessary to provide the proper secondary structure of proteins [97,98,99,100]. Here, we find that without such bias based on the initial structure, Martini can generate overall conformations of Aβ42 with very high accuracy, as indicated by the comparison of chemical shifts to the experimental data, yet lacking secondary structure detectable by the DSSP and similar methods. Therefore, after the planned optimization of the Martini 3 parameters by the developers, it may be an optimal combination of computational cost and simulation accuracy. However, at this moment, its use is limited if detailed conformational changes are to be observed.

The protocol and description provided here (see Appendix A and Appendix B) can be easily employed for routine simulations of most biomacromolecules, with the flexibility to change the force field, water model, and other components of the system. This adaptability can be managed even by less experienced users. While it is recommended to conduct classical MD simulations on supercomputers, access to which is often limited, the use of GPU computations and coarse-grained representations allows these simulations to be successfully run on modern desktop computers, which are usually equipped with up to 32-core CPUs. This democratizes access to scientific research by eliminating the need for substantial budgets, thereby promoting inclusivity in scientific studies.

## 4. Materials and Methods

Detailed protocols on how the MD simulations and analyses were performed, enabling full reproducibility of the results even by inexperienced users, are provided in Appendix A, along with the exemplary scripts (Supplementary Materials) and a detailed description of their implementation (Appendix B). The representative results discussed in this manuscript are based on three force fields: the all-atom ff19SB force field coupled with the four-point OPC water model, and the coarse-grained SIRAH 2 and Martini 3 force fields coupled with their respective coarse-grained water models. For the all-atom simulations, three independent trajectories, each 2 µs long, were run for each system, providing a total of 6 µs per system. For the coarse-grained simulations, a single 20 µs trajectory was run for Martini, while three 20 µs trajectories were run for SIRAH. Monomeric Aβ42 was simulated in its wild-type form and with L17C and L34C mutations to allow the formation of a disulfide bond, which was previously used as a tool to accelerate Aβ42 aggregation [13]. The disulfide bond was treated as both a static covalent bond and a distance restraint, allowing for breaking and formation during the simulation, as presented in the previous work on dynamic treatment [17].

The simulation protocols for the wild-type and disulfide-bond mutants are described in detail in Appendix A, Options 1–2 for the all-atom simulations and Options 3–4 for the SIRAH coarse-grained simulations using the Amber package, as well as Options 5–6 for the Martini coarse-grained simulations and Option 7 for the SIRAH simulations using the Gromacs package. Option 8 demonstrates the use of CHARMM-GUI to generate initial simulation files.

**Figure 10:**
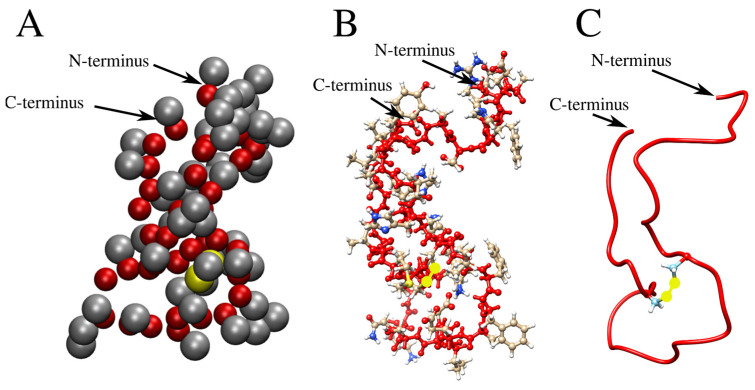
The final structure obtained from the Martini simulation of Aβ42_disul_ is shown in three different representations. (**A**) The coarse-grained structure, where the backbone and sidechain beads are colored in red and gray, respectively. The SC1 beads of Cys17 and Cys34 are highlighted in yellow. (**B**) The all-atom structure, as obtained from the backmapping calculation, is displayed in a stick-and-ball representation. The backbone atoms are shown in red. The sidechain carbon, nitrogen, and oxygen atoms are shown in gray, blue, and red, respectively. The sulfur atoms forming the covalent disulfide bridge are highlighted in yellow. (**C**) The all-atom structure is presented in a ribbon representation: the main chain is shown in red, while sidechains are not displayed, except for Cys17 and Cys34, which are shown in the stick representation with sulfur atoms forming a disulfide bond marked in yellow.

The coarse-grained models were reconstructed to an all-atom representation using the respective scripts described in the protocol (Appendix A) and its accompanying description (Appendix B). The result of mapping coarse-grained simulation structures back to the all-atom representation is illustrated in Figure 10. Specifically, the final structure of Aβ42_disul_ obtained from the Martini simulation with λ = 1 is shown in the van der Waals representation (panel A), the stick-and-ball representation (panel B), and the ribbon representation (panel C). The all-atom structures obtained from the backmapping calculations can be used not only for visualization purposes but also for analysis and comparison with experimental data [48].

Most of the analysis of the simulation results was performed in AmberTools for simulations run in the Amber package and by using Gromacs tools (e.g., gyrate) as well as Python libraries (including MDTraj [101] and Contact Map Explorer 0.7.0) for Gromacs simulations, together with in-house scripts.

Structural analysis, which requires all-atom conformations (Solvent Available Surface Area (SASA) with Linear Combinations of Pairwise Overlaps (LCPO) method [102], secondary structure with Define Secondary Structure of Proteins (DSSP) method [58] was performed using AmberTools [103] based on the original all-atom or reconstructed all-atom trajectories from the coarse-grained models. Carbon and nitrogen chemical shifts were predicted using the most recent version of the SHIFTS 5.6 tool [85], with default options. Free energy maps were calculated using the following formula:

$$\;\Delta F_{n}=-RTln\;\left(I_{n}\;/\;\sum_{a=1}^{N}I_{a}\right)$$

where *R* is the gas constant, *T* is the absolute temperature, $I_{n}$ is the number of structures in *n* bin of 0.02 and 0.1 nm for R_g_ and D_max_, respectively, and $\sum_{a=1}^{N}I_{a}$ is a total number of structures. To determine the most abundant conformations during simulations, a hierarchical agglomerative (bottom-up) clustering method, built in AmberTools’ cpptraj [104], was used.

## 5. Conclusions

In this work, we presented comprehensive protocols for conducting MD simulations on the example of the monomeric form of amyloid-β42 (Aβ42) and its L17C/L34C mutant (Aβ42_disul_). The protocol covers the application of both all-atom and coarse-grained force fields in two widely used simulation packages: Amber and Gromacs. For the all-atom simulations in Amber, we employed the state-of-the-art ff19SB force field coupled with the OPC water model, which has been shown to perform well for both folded and disordered proteins and peptides. We also demonstrated the use of the coarse-grained SIRAH force field in Amber and Gromacs. In Gromacs, we also showcased the application of the broadly-used coarse-grained Martini 3 force field, with corrections for protein-water interactions to better capture the behavior of IDPs, as well as the SIRAH force field. Our protocol not only guides users through the setup and execution of these simulations but also introduces a novel approach for treating disulfide bonds dynamically, allowing them to form and break during the course of the simulation. This dynamic treatment is achieved through the use of distance restraints, providing a more realistic representation of the disulfide bond behavior compared to the conventional static approach. To validate our protocol and demonstrate its utility, we presented new results comparing the conformational dynamics of Aβ42 and Aβ42_disul_ using the various force fields and simulation packages. Our findings clearly illustrate the impact of the disulfide bond on the increased structural similarity between the monomeric form and the fibrillar state of Aβ42, without significantly impacting the secondary structure content. This explains the experimental observation [13] of the increased aggregation rate of the disulfide-bonded Aβ42 mutant. Furthermore, we highlighted the differences in the results obtained from the different methods employed in this study, providing valuable insights into their strengths and limitations for investigating IDPs. In particular, we found that the all-atom Amber ff19SB force field and the coarse-grained Martini 3 force field captured the intrinsically disordered nature of the monomeric Aβ42, while the SIRAH force field overstabilized certain compact conformations. However, all three force fields yield a reasonable agreement with experimental results on such quantities as the radius of gyration and chemical shifts.

By combining the detailed, step-by-step protocols with novel scientific results, this work serves as a valuable resource for researchers interested in studying the dynamics of IDPs and the role of disulfide bonds in their conformational behavior. The protocols can be easily adapted to investigate other IDPs or folded proteins, making them a versatile tool for the broader biomolecular simulation community. Moreover, the insights gained from comparing results obtained with different force fields will help guide force field selection and improvement in future studies of IDPs.

## Figures and Tables

**Table 1:** Average R_g_, D_max_, and SASA with SD (for the Amber and SIRAH force fields, the averages and SDs are calculated over three trajectories, while for Martini it is calculated over a single trajectory with block averaging).

	Amber ff19SB Aβ42	Amber ff19SB Aβ42_disul_	Amber ff19SB Aβ42_dyn-disul_	SIRAH Aβ42	SIRAH Aβ42_disul_	Martini Aβ42 with λ = 1	Martini Aβ42_disul_ with λ = 1
R_g_ (nm)	1.29 ± 0.14	1.27 ± 0.01	1.22 ± 0.07	1.13 ± 0.11	1.10 ± 0.04	1.32 ± 0.01	1.19 ± 0.01
D_max_ (nm)	4.22 ± 0.42	4.16 ± 0.06	4.09 ± 0.21	3.84 ± 0.32	3.48 ± 0.09	4.20 ± 0.04	3.83 ± 0.03
SASA (nm^2^)	37.67 ± 2.78	38.57 ± 0.92	37.73 ± 1.89	36.47 ± 2.63	38.08 ± 0.74	42.90 ± 3.45	39.54 ± 3.87

**Table 2:** Average R_g_, D_max_, and end-to-end distance with SD calculated over a single trajectory in Martini 3 with the solute-solvent scaling parameter λ set to 0.98, 1.00, and 1.02.

	Martini Aβ42 with λ = 0.98	Martini Aβ42 with λ = 1.00	Martini Aβ42 with λ = 1.02	Martini Aβ42_disul_ with λ = 0.98	Martini Aβ42_disul_ with λ = 1.00	Martini Aβ42_disul_ with λ = 1.02
R_g_ (nm)	1.20 ± 0.16	1.32 ± 0.19	1.44 ± 0.22	1.11 ± 0.10	1.18 ± 0.12	1.26 ± 0.13
D_max_ (nm)	3.85 ± 0.59	4.20 ± 0.67	4.52 ± 0.71	3.59 ± 0.47	3.83 ± 0.52	4.06 ± 0.55
End-to-end distance (nm)	2.65 ± 0.94	3.00 ± 1.05	3.32 ± 1.11	2.47 ± 0.83	2.70 ± 0.90	2.93 ± 0.94

**Table 3:** Frequency (in % with SD) of contacts between selected amino-acid residues averaged over trajectories. A contact between two amino acid residues is counted if the Cβ atoms in the all-atom representation or centers of interactions in coarse-grained representations are within a 0.7 nm range.

	Amber ff19SB Aβ42	Amber ff19SB Aβ42_disul_	Amber ff19SB Aβ42_dyn-disul_	SIRAH Aβ42	SIRAH Aβ42_disul_	Martini Aβ42	Martini Aβ42_disul_
Q15-V36	12.54 ± 6.40	9.09 ± 9.94	1.55 ± 1.67	3.45 ± 2.65	0.00 ± 0.00	1.63 ± 0.35	7.11 ± 0.63
L17-L34 (C17-C34)	59.10 ± 29.75	100.00 ± 0.00	100.00 ± 0.00	43.35 ± 7.25	100.00 ± 0.00	5.39 ± 0.75	100.00 ± 0.00
F19-I32	82.34 ± 10.27	58.20 ± 41.84	70.05 ± 36.89	25.72 ± 22.65	26.88 ± 36.38	12.02 ± 0.92	24.72 ± 1.09

**Table 4:** Average secondary content and standard deviations (in brackets) for each of the trajectories.

Force Field	Variant	α-Helices	β-Strand	Other
Amber ff19SB	wt	5.4 (7.3)	23.9 (11.3)	70.7 (7.0)
	CC mutant static	7.7 (3.4)	9.8 (4.6)	82.5 (7.9)
	CC mutant dynamic	5.6 (2.0)	13.1 (8.6)	81.2 (9.3)
SIRAH	wt	0.1 (0.2)	11.4 (3.5)	88.4 (3.7)
	CC mutant	0.0 (0.1)	5.4 (2.8)	94.5 (2.8)
Martini 3	wt	0.8 (2.5)	0.5 (1.6)	98.7 (3.0)
	CC mutant	0.8 (2.5)	0.6 (1.8)	98.6 (3.1)

**Table 5:** Percentage of snapshots with CαRMSD < 6 Å to selected fibril models and standard deviations (in brackets) averaged over trajectories calculated for residues 17–40.

Force Field	Variant	2BEG	2LMN	2M4J	2MXU	2NAO	5KK3	5OQV
Amber ff19SB	wt	0.0 (0.0)	6.3 (10.2)	43.2 (17.6)	2.6 (2.2)	5.9 (4.1)	6.4 (4.9)	2.2 (1.5)
	CC mutant static	0.1 (0.1)	2.6 (3.7)	20.2 (13.8)	34.4 (12.7)	53.7 (20.2)	53.0 (21.2)	31.9 (12.0)
	CC mutant dynamic	1.6 (2.7)	2.3 (2.6)	43.8 (28.7)	12.3 (11.7)	29.4 (13.9)	21.1 (4.5)	7.7 (6.7)
SIRAH	wt	0.0 (0.0)	0.0 (0.0)	10.8 (10.3)	3.6 (6.2)	29.7 (46.5)	8.8 (14.4)	0.3 (0.6)
	CC mutant	0.0 (0.0)	0.0 (0.0)	64.7 (56.0)	26.1 (45.3)	93.1 (5.1)	75.2 (16.4)	20.6 (30.6)
Martini 3	wt	2.5	2.8	13.2	8.5	11.4	8.7	5.5
	CC mutant	1.3	2.3	25.8	7.5	18.1	13.5	5.0

**Table 6:** Lowest RMSD values for selected fibril models calculated for residues 17–40.

Force Field	Variant	2BEG	2LMN	2M4J	2MXU	2NAO	5KK3	5OQV
Amber ff19SB	wt	6.39	5.20	4.24	4.23	4.07	4.06	4.66
	CC mutant static	5.74	4.51	3.46	3.78	3.31	3.28	4.03
	CC mutant dynamic	4.87	4.89	3.68	3.69	3.61	3.60	4.04
SIRAH	wt	6.27	6.22	5.46	5.55	4.69	5.34	5.90
	CC mutant	7.02	6.94	4.48	4.18	3.52	3.86	4.84
Martini 3	wt	3.95	4.40	3.38	3.31	3.82	3.80	3.62
	CC mutant	5.12	4.68	3.52	3.96	3.18	3.34	3.97

**Table 9:** Computational times for various all-atom and coarse-grained approaches for the monomeric Aβ42 with and without disulfide bond treatments are shown with multiple configurations to demonstrate that these simulations can be run on a modern PC or a computer cluster.

Software	Force Field	Disulfide Bond	Number of Interaction Centers:		Timestep, Cutoff Values (Method to Calculate van der Waals/Coulomb Interactions)	Real Time for 1,000,000 Steps	Hardware	
			Peptide	Total			CPU/GPU Model	Core No. and Frequency
Amber 22	All-atom Amber ff19SB	none	627	60,718	2 fs 0.9 nm (PME/PME)	580 min 0 s	2*Intel(R) Xeon(R) E5-2670 v2	20 cores @ 2500 MHz
						8 min 50 s	NVIDIA A100-SXM4-40GB	6912 cores @ 1410 MHz
						38 min 21 s	GeForce GTX 1660 SUPER	3584 cores @ 1800 MHz
		static	609	62,396	2 fs 0.9 nm (PME/PME)	6 min 50 s	NVIDIA GeForce RTX 4090	16,384 cores @ 2520 MHz
						6 min	NVIDIA A100-SXM4-40GB	6912 cores @ 1410 MHz
						40 min 13 s	GeForce GTX 1660 SUPER	3584 cores @ 1800 MHz
		dynamic	611	62,378	2 fs 0.9 nm (PME/PME)	10 min	NVIDIA A100-SXM4-40GB	6912 cores @ 1410 MHz
						40 min 20 s	GeForce GTX 1660 SUPER	3584 cores @ 1800 MHz
	Coarse-grained SIRAH	none	195	4774	20 fs 1.2 nm (PME/PME)	50 min 20 s	2*Intel(R) Xeon(R) E5-2670 v2	20 cores @ 2500 MHz
						4 min 35 s	NVIDIA GeForce RTX 4090	16,384 cores @ 2520 MHz
						3 min 40 s	NVIDIA A100-SXM4-40GB	6912 cores @ 1410 MHz
						9 min 45 s	GeForce GTX 1660 SUPER	3584 cores @ 1800 MHz
		static	195	4926	20 fs 1.2 nm (PME/PME)	3 min 40 s	NVIDIA A100-SXM4-40GB	6912 cores @ 1410 MHz
						7 min 10 s	GeForce GTX 1660 SUPER	3584 cores @ 1800 MHz
Gromacs 2023.5	Coarse-grained MARTINI 3	none	96	6722	20 fs 1.1 nm (cutoff/reaction-field)	1 min 07 s	AMD EPYC 7763	64 cores @ 2450 MHz
						4 min 20 s	Intel(R) Core(TM) i7-5820K	6 cores @ 3300 MHz
						1 min 22 s	GeForce GTX 1660 SUPER	3584 cores @ 1800 MHz
		static	96	6770	20 fs 1.1 nm (cutoff/reaction-field)	5 min 5 s	Intel(R) Core(TM) i7-5820K	6 cores @ 3300 MHz
						1 min 19 s	GeForce GTX 1660 SUPER	3584 cores @ 1800 MHz
	SIRAH	none	195	4644	20 fs 1.2 nm (cutoff/PME)	1 min 43 s	AMD EPYC 7763	64 cores @ 2450 MHz
						4 min 37 s	AMD Ryzen 7 5700G	8 cores @ 3800 MHz
						1 min 59 s	GeForce GTX 1660 SUPER	3584 cores @ 1800 MHz
		static	195	4644	20 fs 1.2 nm (PME/PME)	2 min 53 s	AMD EPYC 7763	64 cores @ 2450 MHz
						7 min 38 s	AMD Ryzen 7 5700G	8 cores @ 3800 MHz
						9 min 38 s	GeForce GTX 1660 SUPER	3584 cores @ 1800 MHz

**Figure A2:**
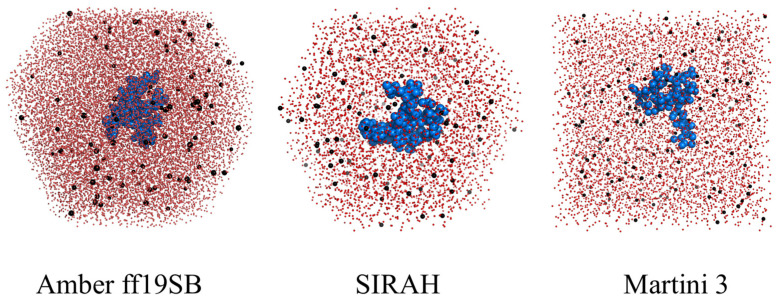
Visualization of the simulation boxes using both all-atom and coarse-grained methods. Atoms or interaction centers (beads) are represented as spheres in red (water), black (ions), and blue (Aβ42 peptide). To save computational time, the MD simulations in the Amber package employed a truncated octahedron box, while the Martini simulations used a standard cubic box.

**Figure A3:**
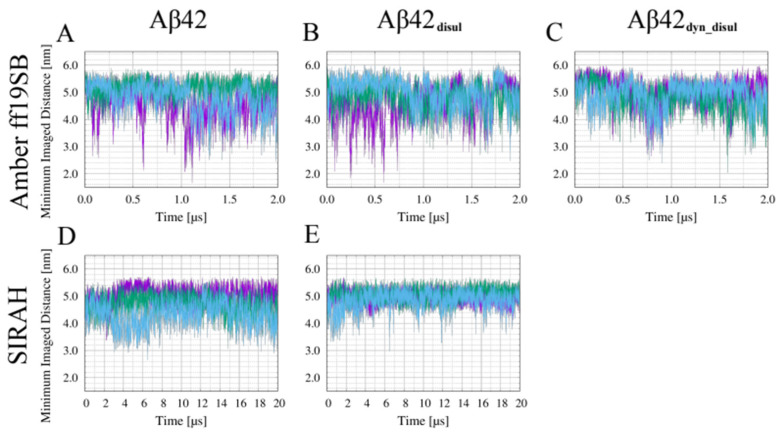
Time evolution of the minimum non-self image distance in the all-atom Amber ff19SB simulations of Aβ42 (**A**), of Aβ42 with the static disulfide bond (**B**), and of Aβ42 with the dynamic disulfide bond (**C**), as well as in the SIRAH coarse-grained simulations of Aβ42 (**D**) and of Aβ42 with the static disulfide bond (**E**). Different colors are used to indicate various trajectories.

**Figure A4:**
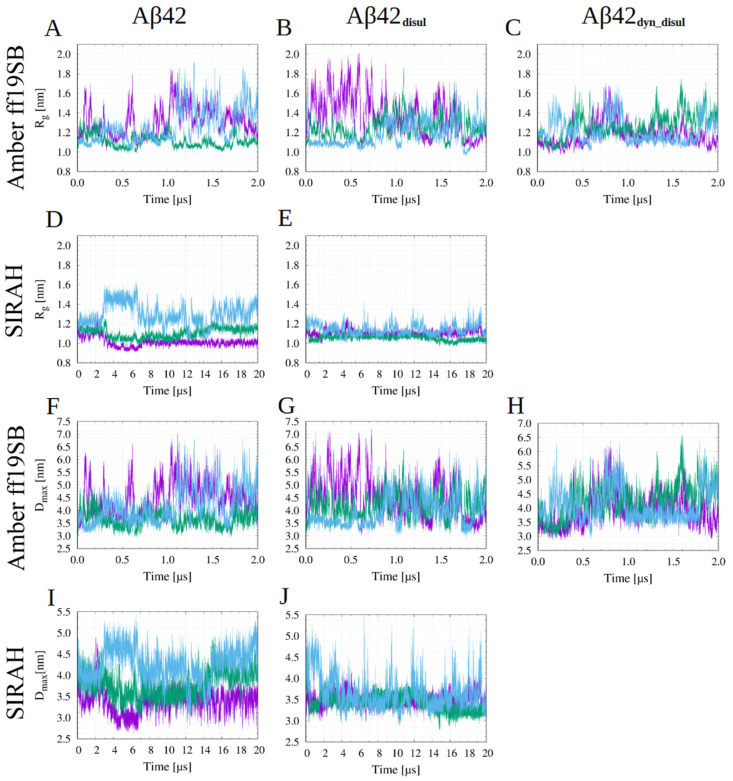
Time evolution of the radius of gyration (**A**–**E**) and of the maximum dimension (**F**–**J**) obtained from the MD simulations employing the all-atom Amber force field ff19SB (**A**–**C**,**F**–**H**) and SIRAH coarse-grained force field (**D**,**E**,**I**,**J**) without the disulfide bond (**A**,**D**,**F**,**I**), with the static disulfide bond (**B**,**E**,**G**,**J**), and with the dynamic treatment of the disulfide bond (**C**,**H**). Different colors are used to indicate various trajectories.

**Figure A5:**
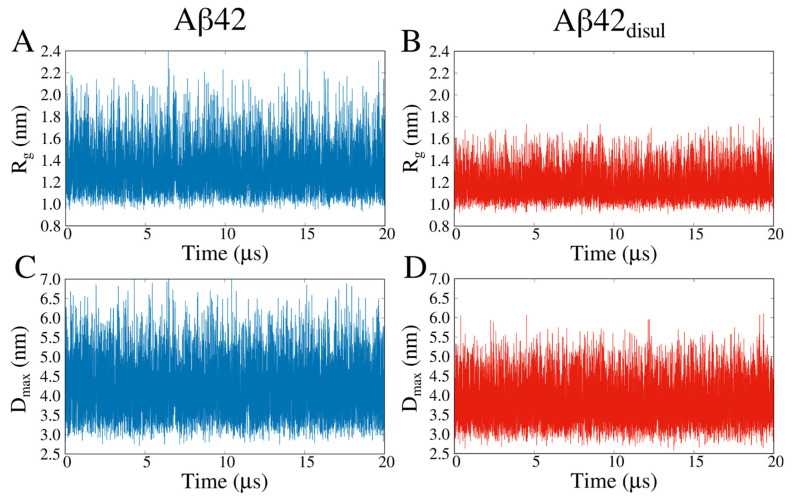
Time evolution of the radius of gyration (**A**,**B**) and of the maximum dimension (**C**,**D**) obtained from the Martini simulations of Aβ42 (**A**,**C**) and Aβ42_disul_ (**B**,**D**) with λ = 1. Both Aβ42 and Aβ42_disul_ exhibit large conformational fluctuations at the thermodynamic equilibrium. Because of the constraint imposed by the covalent bond between Cys17 and Cys34, Aβ42_disul_ exhibits smaller values of R_g_ and D_max_ than Aβ42.

## Data Availability

All of the scripts and initial files necessary to perform the simulations are included in the Supplementary Materials. Additional data is available upon request from the corresponding author.

## Supplementary Materials

The following supporting information can be downloaded at: https://www.mdpi.com/article/10.3390/ijms25126698/s1.

## Appendix A

**Simulation Protocol**

Note: For more details and explanations, the following protocol steps can be compared with descriptions in the Representative Results. Some additional explanations are also given in readme files and as comments in scripts provided in the Supplementary Materials.

This protocol is based on the use of the Ubuntu 22.04 LTS distribution of Linux, but analogous steps should be applicable to other UNIX-based operating systems. The protocol demonstrates how to efficiently run Amber MD calculations on a desktop computer equipped with a CUDA-compatible GPU as well as Gromacs MD simulations on a consumer-type CPU; however, similar calculations can be performed analogously using supercomputers or compute clusters.

The protocol consists of several options; options 1–4 are based on Amber 22 software, while options 5–7 are based on Gromacs 2023.5. Options 1–2 are for all-atom simulations, while options 3–7 demonstrate the use of software for SIRAH and Martini 3 coarse-grained models. Options 2, 4, and 6 include the mutagenesis of selected amino-acid residues to cysteines and the inclusion of a disulfide bond between them in the simulations. Option 8 presents, as an alternative, the use of CHARMM-GUI to process the initial structure and generate example input files. The application of the protocol to simulate other proteins or peptides is discussed in the Representative Results. The general scheme for performing the steps is presented in Figure A1. Users can also look at other available protocols [105].

**Prerequisites**

Before running any simulations, users should complete the following actions:

- Download the archive file from the Supplementary Materials and decompress it.
- Ensure that all the required software is installed (in the case of a personal computer) or loaded in the form of modules (in the case of computer clusters or supercomputers). Some details about that are provided in the equipment table attached to this publication.
- Make sure that software and packages are not only properly compiled but also installed, which may require adding some directories and libraries to operating system paths.
- In the case of Amber software, make sure that the “amber.sh” file is exported (by the “export AMBERPATH/bash.sh” command or by adding this line permanently to the .bashrc file) before starting the procedure, as indicated in the Amber installation guide.

**Use of Amber as a Computational Engine**
**Option 1**
  1. All-atom simulations of monomeric Aβ42 in the ff19SB force field using Amber software.
    1.1. Prepare the PDB input file to run MD simulations using the PDB2PQR server [106]: https://server.poissonboltzmann.org/pdb2pqr (accessed on 10 March 2024)
      1.1.1. Click “Upload a PDB file” button, then click the “Select File” button, and select the PDB file from the extracted directory.
      1.1.2. Fill out the “pH field” with the requested value; in this case, input “7” and select the default option “Use PROPKA to assign protonation states at provided pH”. Select “AMBER” as a requested force field and “AMBER” as an output naming scheme.
      1.1.3. Select the following additional options to ensure proper file processing: (i) ensure that new atoms are not rebuilt too close to existing atoms; (ii) optimize the hydrogen bonding network; (iii) add/keep chain IDs in the PQR file; and (iv) remove the waters from the output file.
      1.1.4. Click “Start Job” and wait for its completion (it usually takes less than a minute).
      1.1.5. Find a file with a “pqr” extension in “PDB2PQR Output Files” and click the “Download” button next to it to download the PQR file. Then rename the downloaded “pqr” file to “***input.pqr***” and copy it to ***amber/tleap***.
    1.2. Enter the directory ***Amber_all-atom_and_SIRAH*** and execute the bash script ***amber/setup_amber.sh*** to perform system setup, energy minimization, and equilibration (it can take up to one hour). Ensure that the script is working properly by checking output files at every step by manual inspection in any text editor.
    1.3. Execute the bash script ***amber/MD1/dyn1/start_dyn1.sh*** to start the production phase (it can take a few days).
    1.4. Execute the bash script ***amber_analysis/run_analysis.sh*** to perform typical analysis of the trajectories with the use of cpptraj, a part of AmberTools package.
    1.5. Execute the script ***amber_analysis/run_analysis_rg_snap.sh*** to find and extract snapshots for the all-atom simulations with the minimum and maximum Rg values.
    1.6. To visualize the snapshots, open PyMOL by typing “pymol” in the terminal or clicking the application’s icon:
      1.6.1. Click File from the top menu, then click Open from the list, and select the PDB file generated during the analysis.
      1.6.2. Click “A” in the side menu on the right hand side of the object name, which, in this case, is the name of the PDB file without the extension and click “remove water” to make the visualization of the protein more clear.
      1.6.3. Click “S” in the side menu on the right hand side of the object name and click “cartoon” to select a cartoon representation of the protein.
      1.6.4. Click “C” in the side menu on the right-hand side of the object name, and then click “spectrum” and select “rainbow” to use a rainbow representation to visualize the N- to C-termini of the polypeptide chain.

**Option 2**
  2. All-atom simulations of a monomeric Aβ42 mutant with a disulfide bond in the ff19SB force field using Amber software.
    2.1. Enter the directory ***Amber_all-atom_and_SIRAH*** and modify the PDB file using PyMOL 2.5 software to mutate two amino-acid residues to cysteines:
      2.1.1. Open PyMOL by typing “pymol” in the terminal window or click the application’s icon.
      2.1.2. Click “S” button in the bottom right corner of the visualization window to show the protein sequence.
      2.1.3. Click “Wizard” from the top window, then click the “Mutagenesis” button from the list and click on the amino-acid residue L17 from the displayed sequence.
      2.1.4. Click on the “No Mutation” button in the right panel and select “CYS”. Ensure that the “No Mutation” button is changed to “Mutate to CYS”, click “Apply”, and click “Done” to mutate selected amino-acid residue.
      2.1.5. Repeat steps 2.1.3 and 2.1.4, selecting the amino-acid residue L34 to mutate it to cysteine.
      2.1.6. Click “File” from the top list and click “Save Molecule…”. Click “OK”, change the directory and name the file ***mutated_disul.pdb*** and then click “Save” to save the file on the computer.
      2.1.7. Close PyMOL
    2.2. Prepare the PDB file by using the PDB2PQR tool (i.e., execute step 1.1), copy it to folder ***amber_disul/smd_disul/tleap*** and change its name to ***input_smd.pqr***.
    2.3. Execute the bash script ***amber_disul/smd_disul/setup_smd_disul.sh*** to perform energy minimization, equilibration, and steered molecular dynamics simulation to bring the sulfur atoms of the two cysteines close to each other. Then execute the bash script ***amber_disul/tleap/cys2cyx.sh*** to convert cysteine residues (CYS to CYX) to include information about the disulfide bond between C17 and C34.
    2.4. Execute step 1.1 to process the file ***amber_disul/tleap/output_smd_cyx.pdb*** using the PDB2PQR tool. After the pqr file is generated, copy it to ***amber_disul/tleap*** as **input_disul.pqr**.
    2.5. Execute bash script amber_disul/setup_amber_disul.sh to perform system setup, energy minimization, and equilibration, if one would like to use the static disulfide bond model, or amber_dyn_disul/setup_amber_dyn_disul.sh, if one would like to use the dynamic disulfide model (it can take up to one hour). Ensure that the script is working properly by checking output files at every step by manual inspection in any text editor.
    2.6. Execute the bash script amber/MD1/dyn1/start_dyn1.sh to start the production phase (it can take a few days) and run steps 1.4–1.6 to analyze and visualize the simulation results.

**Option 3**
  3. Coarse-grained MD simulations of monomeric Aβ42 in the SIRAH force field using Amber software:
    3.1. Enter the directory ***Amber_all-atom_and_SIRAH*** and perform step 1.1 to convert the PDB file to the PQR file.
    3.2. Execute ***amber_sirah/setup_sirah.sh***, which converts the .pqr file using the bash script ***sirah_convert.sh*** to generate a coarse-grained structural model from the all-atom structure and then performs system setup, energy minimization, and equilibration.
    3.3. Execute the bash script ***amber_sirah/MD1/dyn1/start_dyn1.sh*** to start the production phase (it can take a few days).
    3.4. Execute the bash script ***amber_sirah_analysis/run_analysis.sh*** to perform typical analyses of the MD trajectories with the use of cpptraj, a part of AmberTools package. Then execute the script ***amber_sirah_analysis/rg_min_max_cg_snap.sh*** to identify and extract simulation snapshots with the minimum and maximum R_g_ values.
    3.5. Execute the VMD script ***sirah_vmdtk.tcl*** to map the simulation structures from the coarse-grained representation back to the all-atom representation. To do that, perform the following operations:
      3.5.1. Enter the directory ***amber_sirah/MD1/dyn1/*** for backmapping the whole trajectory or the directory ***ptraj_MD1*** (created in step 3.5 automatically) for backmapping a trajectory stripped from water and ions, and open VMD with ***sirah_vmdtk.tcl*** script using the command ***vmd -e ../../sirah_x2.2_20-08.amber/tools/sirah_vmdtk.tcl*** in the terminal.
      3.5.2. Click “File” in the top left corner of the VMD Main window and click “New Molecule”.
      3.5.3. In the pop-up window named Molecule File Browser:
        3.5.3.1. Click “Browse…” and select file system.top, then choose from Determine file type menu: “AMBER7 Parm” and click “Load”.
        3.5.3.2. Click “Browse…” and select file traj.nc, then choose from Determine file type menu: AMBER Coordinates with Periodic Box and click “Load”.
        3.5.3.3. Choose the directory and file name, and click “OK”.
      3.5.4. Click “Extensions” and select “Tk Console” to open a new window with a VMD terminal. and type ***sirah_backmap*** in the Tkconsole to perform the backmapping of all snapshots in the trajectory (alternatively, type ***sirah_backmap now*** to perform the backmapping of only the currently displayed snapshot).
      3.5.5. Save the backmapped trajectory as a single pdb file.
        3.5.5.1. Click “File” in the top left corner of the VMD Main window and click “Save Coordinates”.
        3.5.5.2. In the newly pop-up window named Save Trajectory, choose from the file type list: pdb, then choose from Selected atoms: all, click “Save” and choose the directory and filename, and click “OK”.
    3.6. Perform step 1.6 to visualize the snapshots in the all-atom representation generated in the above step.

**Option 4**
  4. Coarse-grained MD simulations of a monomeric Aβ42 mutant with a disulfide bond in the SIRAH force field using Amber software.
    4.1. Perform steps 2.1 to 2.4 to mutate the selected amino-acid residues to cysteines and to convert the PDB file to the PQR format.
    4.2. Execute ***amber_sirah_disul/setup_sirah_disul.sh*** to convert the .pqr file using the ***sirah_convert.sh*** script to obtain a coarse-grained structural model from the all-atom structure.
    4.3. Execute the bash script ***amber_sirah/MD1/dyn1/start_dyn1.sh*** to start the production phase (it can take a few days).
    4.4. Perform steps 3.4 and 3.6 to analyze the simulation data and to convert coarse-grained structural models to all-atom structures, and then run step 1.6 to visualize the snapshots in the all-atom representation.

**Use of Gromacs as a Computational Engine**
**Option 5**
  5. Coarse-grained MD simulations of monomeric Aβ42 with solute-solvent interaction scaling in the Martini force field using Gromacs 2023.5 software:
    5.1. Enter the directory ***Gromacs_Martini/ABeta*** and execute the bash script ***setup_scripts/setup_martini_AB.sh*** to convert the all-atom structure of Aβ42 to the corresponding Martini coarse-grained model, solvate it, and perform energy minimization (it can take up to an hour).
    5.2. Execute the bash script ***equilibration/run_equilibration.sh*** to perform equilibration simulations (it can take up to an hour).
    5.3. If using a computer cluster, execute 15 bash scripts ***production/qsub_096.sh*** to ***qsub_110.sh*** to run production simulations with the solute-solvent interaction rescaling parameter in the range from λ = 0.96 to λ = 1.1 (it can take up to days to complete). Alternatively, to run the simulations on a desktop computer, execute the bash script ***production/run_on_PC.sh***.
    5.4. Execute the bash script ***production/script_pbc.sh*** to perform the analysis of R_g_. Then execute the bash script ***production/validation.sh*** to calculate average R_g_ values for λ = 0.96, …1.1.
    5.5. Execute the bash script ***production/dmax.sh*** which calculates the maximum dimension of the Aβ42 molecule, D_max_, for each of the simulation structures in each of the trajectories. Then execute the bash script ***production/contactdata.sh*** which calculates contact frequency maps based on the trajectories with λ = 0.96, … 1.1.
    5.6. Open VMD by typing ***vmd peptide.gro run_pbc_lambda1.00.xtc*** in the terminal to visualize the Martini trajectories.
      5.6.1. Type ***pbc box*** in the command line of VMD to display the simulation box
      5.6.2. Click “Graphics”. Click “Representations”. Select “VDW” from the Drawing Method to visualize the system (by default, the backbone beads are depicted in pink, whereas the side chain beads are shown in yellow).
      5.6.3. Use the slider in the main window to move between frames. Use the play button to visualize the whole trajectory.
    5.7. Execute the script ***production/backmapping/backmapping.sh*** to convert the coarse-grained structures generated in the Martini simulations to all-atom structures.
    5.8. Open VMD by typing ***vmd Ab42_input.pdb merged.xtc*** to display the trajectory of Aβ42 in the all-atom representation centered in the simulation box.
      5.8.1. Perform steps analogous to 5.6.1–5.6.3 to visualize the trajectory in the all-atom representation.
      5.8.2. Click “Graphics” to change the background color. Click “Colors”. Choose “Display” and then “Background” from “Categories” and set the color to white. Click **File → Render** and choose **Start Rendering** to visualize and save a selected snapshot.
    5.9. Open ChimeraX to visualize the system.
      5.9.1. Click **File → Open** and choose one of the backmapped structures (e.g., ***production/backmapping/splitted_traj/lambda_1.03/temp/frame_100.pdb***). Go to **Favorites → Settings** and in the first tab, **Background** change the color in **Background color** option.
      5.9.2. Click **Select → Chains → chain ?** to select a whole polypeptide chain. Click **Select → Chains → chain ?** and then select **Actions → Atoms/Bonds → Show** to show side-chain atoms. Click **Actions → Atoms/Bonds → Atom Style → Ball & Stick** to show atoms of the side chain in a different style. Click **Actions → Cartoon → Hide** to hide the ribbon representation and display backbone atoms.
      5.9.3. Click **File → Save…** and in **Files of type** menu, choose the **PNG image (*.png)**, next specify the file name in **File name,** and, after setting the resolution in the **Size** option, use the **Save** button to save the image with the visualization.

**Option 6**
  6. Coarse-grained MD simulations of a monomeric Aβ42 mutant with a disulfide bond in a Martini force field using Gromacs software.
    6.1. Enter the directory ***Gromacs_Martini/ABeta_CC*** and execute the bash script ***setup_scripts/setup_martini_AB.sh*** to convert the all-atom model of Aβ42 to the Martini coarse-grained model, solvate it, and perform energy minimization with the addition of the disulfide bond (it can take up to an hour).
    6.2. Perform steps from 5.2 to 5.9 to simulate, analyze, and visualize the system.

**Option 7**
  7. Coarse-grained MD simulations of monomeric Aβ42 in the SIRAH force field using Gromacs software:
    7.1. Enter the directory ***Gromacs_Sirah*** and execute the bash script ***setup_sirah.sh*** with parameter ***Ab42*** (by typing “bash setup_sirah.sh Ab42” in the terminal) to protonate the all-atom model of Aβ42, convert it to the SIRAH coarse-grained representation, solvate the protein, and create a topology file of the whole simulation system.
    7.2. Run the bash script ***run_min_eq.sh*** with parameter ***Ab42*** (i.e., type “./run_min_eq.sh Ab42” in the terminal) to perform energy minimization and equilibration simulations (it can take a few minutes).
    7.3. Execute the bash script ***run_md.sh*** with parameter ***Ab42*** (by typing “./run_md.sh Ab42” in the terminal) to run the production simulation on a desktop computer.
    7.4. Run the script ***vis_trajectory.sh*** with parameter ***Ab42*** (i.e., type “./vis_trajectoty.sh Ab42“ in the terminal) to visualize the SIRAH trajectory using the VMD program. NOTE: Steps analogous to those described in points 5.4 and 5.5 above can be performed to analyze the SIRAH simulation results.

**Use of CHARMM-GUI to Generate Input Files for the MD Simulations**
**Option 8**
  8. Go to the official website of CHARMM-GUI: https://www.charmm-gui.org/ (accessed on 10 March 2024)
    8.1. Click “Input Generator” from the list on the left-hand side of the website. If this is the first time, register as a non-profit user (if applicable) or log in with your credentials if the account is already registered.
    8.2. Click “Solution Builder” from the expanded list on the left-hand side of the website, click “Browse…” in the “Protein Solution System” menu, and choose the file ***Amber_all-atom_and_SIRAH/amber/tleap/input.pdb***.
      8.2.1. Select the option “Check/Correct PDB Format” to ensure that CHARMM-GUI will fully process the PDB file. Write down the “JOB ID” from the top right corner of the website in case the procedure is not completed in a single use. This number can be used with the Job Retriever in the top left corner of the Input Generator menu to recover the job.
    8.3. Click “Next Step: Select Model/Chain” in the bottom right corner of the website to proceed (each step may take a few seconds to a few minutes, depending on the PDB file and the current server load).
    8.4. Check if the system has properly recognized all amino-acid residues and click “Next Step: Manipulate PDB” in the bottom right corner of the website to proceed further.
    8.5. Check the option list and click “Next Step: Generate PDB” in the bottom right corner of the website to proceed further.
      Note: Mutations or adding disulfide bonds can be performed at this step, but it is not possible to perform both operations simultaneously. To do both, first mutate L17 to C17 and L34 to C34, then generate and download the resulting PDB in the next step, and then repeat the procedure points from 8.4 to 8.7 with the newly generated PDB file, selecting the formation of a disulfide bond.
    8.6. In the “Fit Waterbox Size to Protein Size” box, select the thickness of the water layer around the protein to 23 Å or any other value that would prohibit contacts between periodic images. Click “Next Step: Solvate Molecule’‘ in the bottom right corner of the website to proceed further.
    8.7. Click “Next Step: Setup Periodic Boundary Condition” in the bottom right corner of the website to proceed further. Download the ***step3_pbcsetup.pdb*** file and visualize it to check if the protein structure is fine. NOTE: This PDB file can be used in the standard Amber or Gromacs procedure (options 1–7) after removing water molecules or by manually specifying box size.
    8.8. Select “Amber” in the “Force Field Options:” field and ensure that “FF19SB” is selected as the force field for proteins.
    8.9. Select “Amber” in the “Input Generation Options:” field. Alternatively, “Gromacs” or another computational engine may be selected.
    8.10. Select “NVT Ensemble” in the “Dynamics Input Generation Options:” field and ensure that “Temperature:” is set to 300K.
    8.11. Download the package of the generated input files by clicking “download.tgz” in the top right corner of the website.
    8.12. Extract the downloaded file, then copy ***amber/step3_input.rst7*** as ***system.crd*** to the ***Amber_all-atom_and_SIRAH/amber/tleap*** directory and copy ***amber/step3_input.parm7*** as ***system.top*** to the ***Amber_all-atom_and_SIRAH/amber/tleap*** directory.
    8.13. Execute the bash script ***Amber_all-atom_and_SIRAH/amber/setup_amber_ch-gui.sh*** to perform system setup, energy minimization, and equilibration (it can take up to one hour). Ensure that the script is working properly by checking output files at every step through manual inspection in any text editor.
    8.14. Execute the bash script ***amber/MD1/dyn1/start_dyn1.sh*** to start the production phase (it can take a few days) and perform steps 1.5–1.7 to analyze and visualize the simulation results.

## Appendix B

**Description, options, tips, and tricks to the protocol presented in Appendix A**

When following any tutorial on molecular dynamics, it is important to always consult appropriate manuals, in this case Amber (https://ambermd.org/Manuals.php (accessed on 10 March 2024)) or GROMACS (https://manual.gromacs.org/ (accessed on 10 March 2024)), as they are updated on a regular basis and thoroughly explain all program syntax, flags, and scope of use. Users should be aware that it is often required to process or prepare the PDB file before it can be used as input for MD simulations. Although the presented results are based on a typical situation when a high-quality PDB file is used, one should take into consideration the following steps before starting MD simulations: (i) An initial conformation of a peptide or protein can be downloaded as a PDB file from the PDB, PMDB, or other database or source or predicted using modeling software or servers. (ii) All of the molecules, atoms, and ions that should not be included in the simulation system need to be removed from the PDB file. Please note that it is usually safer to remove hydrogen atoms (by, e.g., using “reduce -Trim *.pdb”) or to use an external tool to predict the correct protonation state for the system (PDB2PQR [106]) as described in Protocol step 1.1, and that external tools and servers, such as CHARMM-GUI [107], can be used for system generation, especially if one would like to include other molecules, such as lipids (micelles, lipid bilayers), or post-translational modifications. (iii) It may be necessary to rebuild missing fragments of the structure. If they are small (a couple of amino-acid residues missing), the task is trivial and can be done manually (approximation of the Cα position in a text editor) or using a visualization tool such as PyMOL or any software for structure prediction (Modeller [108], I-TASSER [109], AlphaFold [110]). However, if a larger fragment is missing in the structure, its modeling may significantly impact the simulation results. (iv) If a study includes mutagenesis, as in the case of a disulfide bond addition to the monomeric Aβ42, it can be performed manually, using a visualization tool such as PyMOL (step 2.1 in Protocol), or using other modeling software. (v) In certain cases, non-standard amino acids could be converted to the standard ones. However, if they are necessary in the system under study, or if other molecules that are supposed to be studied are not present in the force field, they have to be manually parameterized in the case of all-atom force fields or approximated by existing bead types in coarse-grained representations; for all-atom Amber force fields, AnteChamber, which is a part of the AmberTools package [103], or an external server such as PyRed [111] can be used.

Users must ensure that sufficient simulation time is achieved to observe the intended effects of the investigation. Achieving this often necessitates the use of simplifications in both the system and its representation. To ensure proper sampling, it is advisable to routinely simulate multiple trajectories instead of relying on a single one. In some cases, the use of multiple initial conformations can expedite simulations and provide a more comprehensive scan of the conformational space. Moreover, if trajectories converge starting from various points, this convergence can serve as a check for the reliability of the simulation results. In the case of monomeric Aβ42, any method suitable for studies of IDPs should be able to reach convergence within several microseconds of the MD simulation. However, for the examination of oligomeric Aβ42, enhanced sampling methods with multiple starting points may be necessary to obtain reliable results. It should be noted that in the case of IDPs, the convergence of simulation results cannot be defined simply as the stability of a chosen parameter (such as RMSD or R_g_) over time [112]. Instead, it is better characterized as the system’s ability to traverse between similar conformational states multiple times, reflecting the inherently disordered nature of IDPs. One of the straightforward ways to check convergence is to calculate averages over various fragments of the trajectories and compare if they are statistically different or not. However, none of the methods guarantees that the results would not change drastically if the simulation were, for example, one order of magnitude longer. Therefore, it is a good practice to run as many and as long trajectories as possible, preferably with the use of more than a single method, and compare the results with available data. Beginner users are strongly encouraged to learn more about convergence and equilibrium in MD simulations [113,114].

**Simulations in Amber Software**
**Software Tips:**

Users should ensure that the following software requirements are fulfilled: (i) The latest version of the proprietary NVIDIA graphics card driver should be installed. Alternatively, AMD and Intel graphics cards can be used if a special version of Amber is utilized (please refer to the official Amber website for more information about compatibility and performance). Although CUDA Toolkit has to be installed (download available from the NVIDIA official website), it is recommended to install graphics card drivers from the repository and not from the CUDA driver package to avoid compatibility issues during an operating system update. (ii) It is recommended to use the latest version of Amber, a software package that facilitates the setup of simulation systems and enables the execution and analysis of MD simulations (the license for academic use of both Amber and AmberTools is currently free). It is important to verify the installation of all software and ensure that their versions are compatible with the current version of Amber, especially the cmake, gcc, and gfortran compilers, the CUDA toolkit, and the GPU driver. For more information, please refer to: https://stackoverflow.com/questions/6622454/cuda-incompatible-with-my-gcc-version, accessed on 10 March 2024). (iii) After downloading and unpacking AmberTools and Amber files, one should remember to run the update_amber.sh script and the adjust **build/run_cmake** script, e.g., change the flag “DCUDA = TRUE” to enable CMake to install pmemd.cuda, a program that allows running programs on the GPU. Since not all programs are GPU-compatible, it is recommended to first compile the code on the CPU and then on the GPU. If one would like to use multiple CPU cores for MD simulations in the Amber package, another compilation with the flag “DMPI = TRUE” should be performed, and pmemd.MPI was used instead of pmemd to run the simulations. In this case, ensure that MPI or OpenMP is installed.

**Option 1: All-Atom MD Simulations of Aβ42**

The first step of every simulation procedure presented in this publication is to process the input PDB file with the use of the PDB2PQR server [106] as presented in Protocol step 1.1. Note that a standalone version of this software can be used instead [115].

The second step (Protocol step 1.2) is to generate the topology and coordinate files based on the selected force field, and it performs the following operations: (i) The initial structure of Aβ42, as given in the PDB file ***Ab42_input.pdb*** (due to the disordered character of Aβ42, there are no experimentally determined structures of its monomeric form in an aqueous environment; here, one of the conformations obtained in previous MD studies is used as the initial model [27]), is converted to Amber topology and coordinate files using the **tLeap** program with the ff19SB force field for proteins. (ii) The protein is solvated with the OPC water, and co-optimized ions (sodium and chloride) [37] are added to neutralize the system and reach the physiological salt concentration of about 0.15 M. Counter-ions are calculated automatically if a *-1* number is specified in the tLeap input file (e.g., “addions2 system Na+ −1”), but salt concentration needs to be inserted manually. For additional details on setting up the salt concentration, please refer to the Amber tutorial [116] or, alternatively, on the CHARMM-GUI site, the input generator tool allows one to calculate the salt concentration. Finally, (iii) the system is placed in a selected type of periodic boundary condition (PBC) box (in this case: truncated octahedron), the size of which is determined based on the added water layer.

The provided scripts for all-atom simulations in Amber utilize a default set of the protein force field (ff19SB) coupled with the recent and most versatile water model (OPC). Despite the higher computational cost of using the four-point OPC water model, it is a recommended model to be used in Amber all-atom force fields, as it provides much better results if solute-solvent interactions are important. This is especially true for IDPs, such as the monomeric Aβ42, when compared to classical three-point models, such as TIP3P [54]. It should be noted that users can easily switch between various force field versions and water models by simply modifying the top lines in the leap.in file according to the official Amber website and a manual or publications of unofficial force field versions, such as, for example, ff14IDPs [117], designed specifically for IDPs.

Once the simulation system is set up, energy minimization is performed. Parameters of the energy minimization procedure are specified in the input file ***md_min.in***, and the bash script ***start_min.sh*** is used to copy the generated topology and coordinate files and to run the minimization. During the energy minimization, the optimization method is switched from the steepest descent to the conjugate gradient, combining stability with efficiency. Restraints are typically imposed to maintain the initial positions of solute-heavy atoms.

If the energy minimization cannot be executed successfully, one should check for any warnings or issues in the tLeap log file (leap.log) and consider increasing the number of minimization steps in both algorithms. In the rare event that the steepest descent calculation gets stuck in a high-energy local minimum, the subsequent conjugate-gradient calculation may fail. In such a situation, one may try to perform only the steepest descent minimization followed by a very short equilibration run (below ps) with a significantly reduced time step (several orders of magnitude shorter than usual) to move the system out of this conformation. It is advisable to repeat the standard minimization procedure afterward.

The system is equilibrated in two successive steps. In the first one, equilibration is performed using the CPU to increase stability in the NPT ensemble. The second step utilizes the GPU to expedite the process without risking instabilities. For the first equilibration run (on the CPU), all simulation parameters are specified in the input file ***md_eq1.in***. The bash script ***start_eq1.sh*** is used to initiate the first equilibration run. For the second equilibration run (on GPU), the input file ***md_eq2.in*** and the bash file ***start_eq2.sh*** are used. The simulation time step is set to 2 fs, and the total equilibration time is 1 ns, which should be adequate for a typical system without a lipid bilayer to reach the proper temperature and density. This time can be increased if necessary. During the equilibration process, initial velocities are generated, and the system is heated up to the production temperature (300 K) to prevent potential steric clashes. In systems with multiple components, such as ligands or lipids, restraints are applied to groups of atoms to ensure proper system preparation and to prevent component dissociation resulting from minor structural imperfections that could lead to localized high energy. Equilibration in the NPT ensemble establishes the correct system density, allowing the production phase of the simulation to proceed in the NVT ensemble. A cutoff distance of 0.9 nm is set, and the simulation uses a Langevin thermostat with a collision frequency of 2 ps^−1^ and a Berendsen barostat with isotropic position scaling. To calculate the long-ranged electrostatic interactions, the Particle-Mesh Ewald (PME) method is used, which speeds up the calculations. For GPU calculations on NVIDIA GPUs, one can use the ‘CUDA_VISIBLE_DEVICES’ flag to select which GPU unit to use when multiple GPUs are available. Additional equilibration with the use of a GPU can be performed. To do this, users need to create a directory named ‘**eq3**’, copy the contents of the ‘**eq2**’ directory into ‘**eq3**’, and rename the bash script ‘**start_eq2.sh**’ to ‘**start_eq3.sh**’. Then modify the script to initiate the simulation from the restart file from the second equilibration run: ‘**-c system_eq2.crd**’, and generate a third restart file: ‘**-r system_eq3.crd**’. Finally, execute the bash script ‘**start_eq3.sh**’ to run the third equilibration simulation.

The scripts ***start_min.sh***, ***start_eq1.sh*** and ***start_eq2.sh*** copy the necessary input coordinate and topology files from the respective subdirectories of the previous steps. They then initiate simulations that generate trajectory files, which contain coordinates of the system (snapshots) saved at specified intervals, a ‘restart’ file with coordinates and velocities (after equilibration), output with simulation information, and details regarding the simulation’s progress.

The final simulational step (see Protocol step 1.3) is to run production MD simulations. All the parameters for the MD simulation are specified in the input file ***md_dyn1.in***. In this phase, the simulation time step is set to 2 fs for all-atom simulations, and the production time is 1 μs. For the coarse-grained simulations, the simulation time step is set to 20 fs, and the production time is 20 μs. To enhance the simulation speed and stability, the NVT ensemble is typically used for production trajectories [118]. The cut-off distance remains at 0.9 and 1.2 nm for all-atom and coarse-grained simulations, respectively, and Langevin dynamics with a collision frequency of 2 ps^−1^ at a temperature of 300 K are applied. The only difference compared to the equilibration phase is the absence of pressure control since a constant volume, not pressure, is maintained. The SHAKE algorithm is used to constrain hydrogen bond lengths to allow for a longer time step and higher stability in the simulation.

If one needs to extend the simulations, navigate to the folder ***MD1/dyn2***, set the desired production steps using the ‘nstlim’ flag, and execute the bash script ***start_dyn2.sh***. This extension can be performed multiple times, but make sure to use appropriate file naming. To run multiple trajectories, duplicate the input folder ***MD1*** (i.e., ***MD2***, ***MD3***, etc.) as many times as needed (a reasonable minimum for averaging is three trajectories) and follow the analogous steps as for MD1.

During the simulations, the following files are generated: **traj.nc** file containing atom/bead coordinates; **system_dyn*.nc** restart file with coordinates and velocities, which allows to restart the simulation; **min.out** file with simulation information output; **mdinfo** file with information about the simulation progress, speed of the simulation, and the estimated time to finish the production run.

After the simulations are finished, there are several analyses that allow one to study the structural properties of the system based on the MD trajectories. The bash script ***amber_analysis/run_analysis.sh*** performs the following operations (see Protocol step 1.4): (i) creates folders for analysis for each trajectory listed in the script and runs the ***cpptraj*** program with commands provided in the input file ***ptraj_traj.in***. This process generates topology and coordinate files in the **ptraj_MD*** folders after centering the solute in the periodic boundary condition box (using the ‘autoimage’ command), followed by the removal of water molecules and ions. Caution while using the ‘autoimage’ command in more complex systems is recommended, as it may cause unwanted shifts of solute molecules. This step is important for speeding up all the following analyses. (Note: if there is any analysis that needs to incorporate initial coordinates or water molecules and ions, it should be run on the initial coordinate and topology files). Please note that it is not recommended to run any further analysis in a single script if the RMSD calculation has been performed, as it may impact the results. Conversely, be cautious about the orientation of the solute in the PBC box when calculating RMSF, or other analyses requiring superpositioning with a reference structure. (ii) Runs **cpptraj** program with the commands included in the input files **ptraj_rmsd_rg.in**, ***ptraj_pdb.in*,** and ***ptraj_image_distance.in***. ***Ptraj_rmsd_rg.in*** calculates RMSD, R_g_, and RMSF; ***ptraj_pdb.in*** creates PDB files from Amber topology and trajectory files; and ***ptraj_image_distance.in*** calculates the minimum non-self imaged distance, which is used to determine if the periodic box is of sufficient size to prohibit interactions between periodic images. (iii) Copies all the resulting text and PDB files with the overwritten names of each trajectory to the corresponding folders (rg, rmsd, rmsf, pdb, mindist).

To visualize snapshots for the all-atom simulations with the minimum and maximum R_g_ values, the batch script ***amber_analysis/rg_min_max_snap.sh*** performs the following operations (see Protocol step 1.5): is searching for the minimum and maximum values of R_g_ and identifying the corresponding structures; then it extracts those structures from the trajectory file and saves them in the pdb format. Visualization is presented in Protocol step 1.6.

**Option 2: All-Atom MD Simulations of Aβ42 with an Artificially Added Disulfide Bond**

The second set of MD simulations in Amber is performed with the disulfide bond present in the monomeric Aβ42. However, as this system does not include cysteines that can form disulfide bonds in the native state, two amino-acid residues have to be mutated to cysteines. Note that in Amber software, when simulating a protein that contains disulfide bonds in its wild-type form, the amino-acid three-letter code in the PDB file must be changed from CYS to CYX to reflect these bonds. Additionally, the tLeap input file should include a line specifying which sulfur atoms are bonded together. If the simulation aims to reduce or remove these disulfide bonds, the amino-acid code should remain as CYS.

Mutations in the PDB file can be performed manually with a text editor or using visualization, modeling, or structure prediction tools. If a single-point mutation is planned, then the fastest approach should be to use a manual method; however, if more extensive modifications are planned, it may be necessary to use more advanced methods such as I-TASSER or AlphaFold, as described briefly below.

**Manual mutation**

To manually mutate any amino acid residue in the PDB file, except for Gly, remove all atoms from the side chain except for the CB atom and change the three-letter code of the residue to the desired amino acid. For Gly, change the amino-acid code for the CA atom only. During system preparation, tLeap will automatically assign any missing atoms. Be cautious as this process may lead to steric clashes; therefore, perform the minimization and equilibration steps with extra care.

**Visualization-tool mutation (PyMol)**

The PyMOL Mutagenesis Wizard tool can be used to change any of the amino-acid residues (Protocol step 2.1). The tool also allows the user to choose a rotamer, but it’s important to note that they may still result in steric clashes (for more information, see the official pymol explanation of the function [119].

**Use of a structure prediction tool**

If extensive mutations or deletions are planned, using advanced software such as I-TASSER [120] or AlphaFold [110], which predicts structures based on the provided sequence and known protein structures, may be advisable. Such tools not only have extensive checks to avoid steric clashes caused by the mutation but, to some extent, can also incorporate local or even global conformational changes upon mutation; therefore, they should provide more reliable initial structures than manual modifications.

**Insertion of a disulfide bond**
**Selection of the conformation with cysteine residues in the proximity**

In some cases, there may be a conformation in the trajectory of the wild-type protein where amino acid side chains that need to be mutated are in close proximity. To check this, one can use cpptraj with the ‘distance’ command to measure the distance between the desired CB or CG atoms. If a suitable conformation is found, it can be extracted and then mutated as described above to create the initial conformation.

**SMD of a structure to bring cysteines together**

If the previous method fails or if there is no trajectory without mutation available, another option is to run a short steered MD (SMD) simulation (Protocol step 2.3). After mutating the chosen amino-acid residues to cysteines, the initial steps (system setup, minimization, and equilibration) should follow the same procedure as for the wild-type version. Then, run a short production run of SMD with distance restraints imposed on the sulfur atoms in cysteine side-chains, reducing the distance between them to 0.2–0.25 nm. If the simulation were to fail, slow down the pulling speed by increasing the number of steps in the md_smd.in file. Convert the last snapshot to a pdb file and, after verifying the structure with PyMOL or other molecular visualization software, use it as the initial conformation. The corresponding scripts and input files can be found in the directory ***amber_disul/disul_smd***.

**Selection of a disulfide-bond model**

There are two options for applying disulfide bonds in MD simulations using the Amber software: one can simply use covalent bonds, which can be present or absent throughout the entire simulation (static treatment), or one can use a pseudopotential that allows for breaking and reforming disulfide bonds during the simulation (dynamic treatment).

The static disulfide-bond treatment is performed by the bash script ***amber_disul/setup_amber_disul.sh*.** This script performs operations analogous to those described for simulations in Amber without disulfide bonds with two modifications: (i) The initial structure in the PDB file ***amyloidB_withSS.pdb*** is used as input. This structure of Aβ42 contains a disulfide bond between Cys17 and Cys34, indicated as CYX in the PDB file. (ii) The tleap program has an additional command ***bond*** to set disulfide bonds between the sulfur atoms. Tleap detects and incorporates the disulfide bonds into the topology file.

The dynamic disulfide-bond treatment is performed by the bash script ***amber_dyn_disul/setup_amber_dyn_disul.sh***. This script performs operations analogous to those described for simulations in Amber without disulfide bonds with three modifications: (i) The initial structure in the PDB file ***amyloidB_dynSS.pdb*** is used as input. This structure of Aβ42 contains a disulfide bond between Cys17 and Cys34, which is indicated as ‘CYS’ in this PDB file, in contrast to the static disulfide bond ‘CYX’ version. (ii) There is a third equilibration step with restraints placed on the sulfur atoms to mimic a breakable bond. (iii) Both the third equilibration and production steps have an additional input file that indicates the sulfur atoms that are restrained.

It is important to note that this approach represents a heavily simplified description of disulfide bond formation and disruption, and should therefore be used with caution. Classical all-atom force fields cannot capture chemical reactions, and therefore the hydrogen atom in the thiol group of cysteine remains present throughout the simulation. Nevertheless, we have found this approach to be effective in both the bound and unbound states of cysteines, as exemplified by bovine ribonuclease A simulations [17], whose results are not only in agreement with coarse-grained simulations but also have been confirmed very recently by independent experimental studies [121]. It should also be noted that the approach of using identical beads for the bound and unbound cysteine residues is commonly utilized in coarse-grained force fields, including Martini and SIRAH, also allowing for disulfide bond formation and breaking [56,122].

**Options 3 and 4: SIRAH Coarse-Grained MD Simulations of Aβ42 without and with Disulfide Bonds**

The cash script ***amber_sirah/setup_sirah.sh*** (see Protocol step 3.2) converts the .pqr file using ***sirah_convert.sh***. The .pqr file can be prepared with charge assignment using the PROPKA tool, for example, via the PDB2PQR server (protocol step 1.1). The pqr files are ready to be used as input for tLeap, with syntax analogous to that of all-atom simulations. The only changes required are in the names of the protein and water parameters, which are also explained in the ***README*** file provided by the SIRAH developers.

Similarly, the bash script ***amber_sirah_disul/setup_sirah_disul.sh*** (see Protocol step 4.2) uses the coarse-grained SIRAH force field and models the Aβ42 double Cys mutant based on the PQR file. In the case of the coarse-grained simulations using ***amber_sirah_disul***, the tleap input includes an additional command to set a disulfide bond between the appropriate coarse-grained beads.

The bash script ***amber_sirah_analysis/rg_min_max_cg_snap.sh*** (see Protocol step 3.4) is used to perform the analysis for the visualization of the SIRAH coarse-grained simulation results. This script performs operations analogous to those from the all-atom procedure with two modifications: (i) The extracted structures are saved in Amber topology and coordinate files. (ii) The VMD script ***sirah_vmdtk.tcl*** is used to map the simulation structures from the coarse-grained representation back to the all-atom representation. This script is provided by SIRAH developers, and is included in the SI file in the ***Amber_all-atom_and_SIRAH/sirah_x2.2_20-08.amber/tools*** directory. After opening VMD with the trajectory file, the backmapping is performed with the command ***sirah_backmap***, which backmaps every snapshot in the trajectory, or with the command ***sirah_backmap now***, which backmaps only the current snapshot.

**Simulations in Gromacs Software**

Access to a computer cluster is useful but not necessary to perform the Martini simulations. The MD simulations can be run on a desktop computer with a multi-core CPU. However, the main advantage of using a computer cluster is that many simulations can be run simultaneously on separate compute nodes. Here, we provide bash scripts to execute the Martini simulations on a computer cluster equipped with the Slurm queue system and for simulations run on a desktop computer. On a supercomputer, or computer cluster, the installation of necessary software is typically performed by the system administrator and can be loaded using the “module load” command.

**Option 5: Martini Coarse-Grained MD Simulations of Aβ42**

General steps to run simulations in Gromacs are similar to the ones in Amber. The bash script ***setup_scripts/setup_martini_AB.sh*** (see point 5.1 in the protocol) performs the following operations: The initial structure of Aβ42, as given in the PDB file ***Ab42_input.pdb***, is converted to a coarse-grained representation using the martinize2 program. Martini topology and structure files are generated based on the information from the provided PDB file. It is important to note at this point that if one would like to use another version of the Martini force field, one simply has to copy the force-field parameter files to the directory ***Gromacs_Martini/ABeta/setup_scripts*** and update the ***setup_martini_AB.sh*** script accordingly. Then the coarse-grained protein structure is placed in a cubic box with a side length of 9 nm using the Gromacs editconf tool. In the next step, the protein is solvated using the Python script ***insane.py***. Sodium and chloride ions are added to neutralize the system and reach the physiological salt concentration of 0.15 M. Note that the flag “-salt” applied to the ***insane.py*** script indicates the salt concentration in molar units. Finally, energy minimization is performed. The parameters of the energy minimization procedure are specified in the Gromacs input file ***minimization.mdp***.

The goal of performing a series of MD simulations with different λ-values is to determine the optimal value of parameter λ that yields the best agreement between the simulation and experiment [48]. Note that λ = 1 corresponds to unscaled solute-solvent interactions, i.e., to the original Martini 3 force field. The force-field parameters describing non-bonded interactions between the protein and water beads are re-scaled by a factor λ using the bash script ***rescaling.sh***. The values of parameter λ are set here to 0.96, 0.97 … 1.10 and can be manually modified, if necessary.

The bash script ***equilibration/run_equilibration.sh*** (see point 5.2 in the protocol) performs equilibration simulations. The same range of λ-values as in the system setup step described above (i.e., λ = 0.96, 0.97 … 1.10) is used here. All the parameters of the equilibration simulations are specified in the Gromacs input file ***equilibration.mdp***. Importantly, the integration time step is set to 2 fs to allow for slow relaxation of the system. The equilibration time is set to 10 ns, which should be enough for typical protein systems to reach the specified temperature and density.

In the first step, the script ***run_equilibration.sh*** generates a binary tpr file that contains information about all the simulation parameters (including force field parameters), about the system topology, and about the starting coordinates and velocities of all the beads forming the simulation system. Here, the starting velocities are generated on the basis of the Maxwell-Boltzmann distribution at the specified temperature. In the second step, the MD simulation is started using the tpr file as input. The simulation box size after the equilibration can be checked at the end of the ***eqb1.00.gro*** file. Here, the simulation box is a cube with a side length of 9.23 nm.

Production runs are performed by executing the bash scripts ***qsub_096.sh*** … ***qsub_110.sh*** (see point 5.3 in the protocol). Each of the scripts starts one simulation with a specified λ-value, ranging from λ = 0.96 to λ = 1.1. All the simulation parameters are given in the Gromacs input file ***dynamic.mdp***. Here, the integration time step is set to 20 fs, and the total simulation time is 20 μs. The temperature and pressure are kept constant at T = 300 K and p = 1 bar, respectively, using the velocity-rescaling thermostat and the Parrinello-Rahman barostat. Nonbonded interactions are treated with the Verlet cutoff scheme. The cutoff for van der Waals interactions is set to 1.1 nm. Coulomb interactions are treated using the reaction-field method with a cutoff of 1.1 nm and a dielectric constant of 15. Frames are saved every 1 ns.

Similarly, as in the equilibration procedure, the scripts ***qsub_096.sh*** … ***qsub_110.sh*** first generate binary tpr files containing all the information needed to start the simulations (i.e., system topology, MD simulation parameters, and starting coordinates and velocities of the beads). Then the MD simulations are started using the tpr files as input. Each of the production simulations takes about two days on a 12-core compute node.

The following files are generated in the course of the simulations: ***run_pbc_lambda*.gro*** files containing the Cartesian coordinates of the beads at the final step of the simulation; ***run_pbc_lambda*.xtc*** trajectory files; ***run_pbc_lambda*.edr*** binary files that contain time-series non-trajectory data such as energy breakdowns, temperature, and pressure of the simulation system; ***run_pbc_lambda*.log*** files containing information about the overall performance of the simulation. These files also include energy breakdowns as well as the instantaneous values of the pressure and temperature of the simulation system; ***run_pbc_lambda*.cpt*** binary files are required for restarting the simulation. The complete information about the system state is stored in these checkpoint files.

After the production simulations are completed, bash the script ***production/script_pbc.sh*** (see point 5.4 in the protocol) and perform the following tasks: Firstly, the Gromacs trjconv tool with the flags “-pbc mol -center” makes the Aβ42 peptide centered inside the simulation box, taking into account the periodic boundary conditions. The resulting trajectory files are named ***run_pbc_lambda*.xtc*** and moved to directories ***AB_**** for all the simulated values of parameter λ. Secondly, the radius of gyration of the Aβ42 molecule, Rg, is calculated for all of the simulation structures and recorded in the output files rg_run_pbc_lambda*.xvg, which are moved to the folder ***/analysis_results/RG***. It should be noted that the radius of gyration is calculated here using the coordinates of all the beads forming Aβ42.

Next, the bash script ***production/validation.sh*** (see point 5.4 in the protocol) calculates the average radius of gyration for each of the trajectories with λ = 0.96, …, 1.1. The statistical errors are estimated using block averaging. Namely, a given trajectory is divided into 10 blocks of 2 μs each, and the average value of R_g_ is calculated for each of the blocks. The standard deviation of these black-averaged R_g_ values estimates the statistical error of the average value of R_g_ in the whole trajectory.

Then the bash script ***production/dmax.sh*** (see point 5.5 in the protocol) calculates the maximum dimension of the Aβ42 molecule, D_max_, for each of the simulation structures in each of the trajectories. D_max_ is defined as the distance between the two farthest beads of Aβ42. The D_max_ values are recorded in files ***dmax*.xvg*** and moved to folder ***dmax***.

Finally, the bash script ***production/contactdata.sh*** (see point 5.5 in the protocol) calculates contact frequency maps based on the ***run_pbc_lambda*.xtc*** trajectory files. The contact frequency maps are recorded in the file ***contact*.xvg*** and moved to the folder ***contactmap***. The only parameter in the contact map calculation is the cutoff distance below which a contact is assumed to exist between a pair of beads. Here, the default value of 0.7 nm is used (flag cutoff = 0.7 in the Python script ***contactdata.py***). Thus, if the distance between any bead of a given amino-acid residue and any bead of another amino-acid residue is smaller than 0.7 nm, then this residue pair is deemed to be in contact.

The Aβ42 conformations sampled in the Martini simulations and saved in the trajectory files ***run_pbc_lambda*.xtc*** can be conveniently visualized using the VMD program (see point 5.6 in the protocol). In addition, the bash script ***production/backmapping/backmapping.sh*** (see point 5.7 in the protocol) converts the coarse-grained structures generated in the Martini simulations to all-atom structures. This script is based on the backmapping algorithm introduced by Wassenaal et al. [123] and performs the following operations: (i) an all-atom topology file is created using the PDB file ***Ab42_input.pdb*** and the CHARMM36 force field; (ii) a single coarse-grained structure is extracted from a given trajectory file, run_pbc_lambda*.xtc; (iii) the coarse-grained structure is mapped back to an all-atom structure using the ***backward.py*** program; this backmapped structure is relaxed in several short cycles of energy minimization and position-restrained MD simulations with the CHARMM36 force field; (iv) the two points above are iterated over all the coarse-grained structures obtained from the Martini simulations, combining the resulting all-atom structures into a single trajectory file.

The backward program consists of several scripts and mapping definition files, including the Python script ***backward.py*** which conducts the actual backmapping; the bash wrapper ***initral-v5.sh***, which performs the energy minimization and position-restraint MD simulations using Gromacs with the CHARMM36 force field; files ****.map,*** which contain definitions of the mapping between the all-atom and coarse-grained representations; and the Python script ***init .py*** to interpret the map files. It should be noted that the all-atom structures can be used not only to visualize the simulation trajectory (see points 5.8 and 5.9 in the protocol) but also to perform analyses analogous to those in points 5.4 and 5.5 of the protocol.

**Option 6: Martini Coarse-Grained MD Simulations of Aβ42 with an Artificially Added Disulfide Bond**

The bash script ***ABeta_CC/setup_scripts/setup_martini_AB.sh*** (see point 6.1 in the protocol) sets up the Martini simulations of the Aβ42 Leu17Cys/Leu34Cys mutant, denoted here by Aβ42_disul_. This script performs operations analogous to those described in the previous subsection 5 for the Aβ42 variant without the disulfide bond, with two modifications: (i) The PDB file ***amyloidB_withSS.pdb*** with an Aβ42_disul_ structure is used as input. This structure contains a disulfide bond between Cys17 and Cys34. (ii) The martinize2 program is called with the flag “-cys auto”. With this flag, martinize2 automatically detects and incorporates disulfide bonds into the topology file as constraints on the distance between the cysteine side-chain SC1 beads. Here, the distance between the SC1 beads of Cys17 and Cys34 is constrained at 0.24 nm using the LINCS algorithm [124]. Importantly, accurately including disulfide bonds in the input structure may require a high-resolution initial structure or preprocessing, such as all-atom energy minimization.

**Application of Martini Coarse-Grained MD Simulations to Structured and Mix-Folded Proteins**

It should also be noted that proteins with well-defined structures can be simulated using the Martini coarse-grained model with just a few modifications to the protocol presented in this work. To set up Martini simulations of a protein with a well-defined structure, it is necessary to use an elastic network [100], i.e., a set of harmonic restraints (or so-called “rubber bands”) that ensure that the secondary and tertiary structure of the protein is preserved in the course of the simulation. To this end, the martinize2 program should be called with additional flags, e.g., “-elastic -ef 700.0 -el 0.5 -eu 0.9 -ea 0 -ep 0 -scfix”. This imposes harmonic restraints between backbone beads within the lower and upper distance cut-offs of 0.5 and 0.9 nm, respectively. The spring constant of the harmonic restraints is set to 700 kJ mol^−1^ nm^−2^. In addition, the flag “-scfix” adds extra torsional potentials between backbone beads and sidechain beads [125]. These potentials are included in the force field to prevent unphysical flipping of side chains in β-strands.

It is important to note that the elastic network is created on the basis of a reference secondary structure that needs to be indicated to the marinize2 program, which can be completed by adding the flag “-dssp mkdssp”. With this flag, the DSSP program is used to determine the secondary structure elements based on the input structure. DSSP recognizes secondary structure based on the positions of both heavy atoms and hydrogens; therefore, it is important to use a high-resolution initial structure or perform preprocessing, such as all-atom energy minimization, to generate accurate secondary structure restraints.

To set up Martini simulations of mix-folded proteins, in which folded protein domains are linked by intrinsically disordered regions, it is recommended to follow the method and procedures introduced by the Lindorff-Larsen group [48].

**Option 7: SIRAH Coarse-Grained MD Simulations of Aβ42**

Although SHRAH and Martini are quite different in various aspects, the general protocol for running MD simulations with these two force fields is actually the same when using Gromacs. The bash script ***setup_sirah.sh*,** called with parameter ***Ab42*** (see point 7.1 in the protocol), performs the following operations: (i) the initial structure of Aβ42, as given in the PDB file ***Ab42.pdb***, is protonated at pH = 7.0 using the PDB2PQR 3.6.2 software (with the with-ph = 7.0 flag) and converted to a coarse-grained representation using the cgconv.pl program. The SIRAH topology and structure files are generated based on the information from the provided PDB file. (ii) The coarse-grained protein structure is placed in a cubic box of side length 9 nm using the Gromacs editconf tool (with the -box 9 9 9 flag) and solvated using the Gromacs solvate tool. (iii) Sodium and chloride ions are added to the simulation box in order to neutralize the system and reach the physiological salt concentration of 0.15 M. Note that the flags “-np 66 -pname NaW -nn 63 -nname ClW” define the numbers of ions in the system. Users must manually recalculate the numbers of ions if they want to use a different ionic strength in their simulations.

The bash script ***run_min_eq.sh*,** called with parameter ***Ab42*** (see point 7.2 in the protocol), performs energy minimization and equilibration simulations. The energy minimization is performed in two subsequent steps with parameters given in files ***md_sim_parameters/em1_CGPROT.mdp*** and ***md_sim_parameters/em2_CGPROT.mdp***. The equilibration simulations are conducted at constant volume (NVT ensemble) in two subsequent steps, respectively, with hard and soft restraints on the coordinates of the backbone beads. The parameters of these equilibration simulations are specified in the Gromacs input files ***md_sim_parameters/eq1_CGPROT.mdp*** and ***md_sim_parameters/eq2_CGPROT.mdp***. Analogously, the parameters of the production simulation are given in the input file ***md_CGPROT.mdp***.

The bash script ***run_md.sh,*** called with parameter ***Ab42*** (see point 7.3 in the protocol), starts the production simulation at constant pressure (NPT ensemble). Next, when this simulation is completed, it centers the protein in the simulation box, taking into account the periodic boundary conditions. The resulting trajectory file is named ***Ab42_cg_md_pbc.xtc*** and is placed in the directory ***run_md***. The final configuration of the simulation system is saved in the ***Ab42_cg_md.gro*** file. With these two files, one can analyze the SIRAH simulation trajectory in the same way as the Martini simulation trajectories are analyzed here (see points 5.4 and 5.5 in the protocol).

It should be noted that in order to perform SIRAH simulations of a different protein using Gromacs, users can simply copy a PDB file of their protein (e.g., ***myprotein.pdb***) to the ***GROMACS_SIRAH*** directory and perform steps from 7.1 to 7.4 of the protocol by successively executing the four scripts with the input parameter being the name of the PDB file (e.g., ***myprotein***).

**Option 8: Use of CHARMM-GUI for MD Simulations of Aβ42**

CHARMM-GUI stands as a versatile tool, facilitating a range of essential functions for manipulations of PDB files and generation of initial files for simulating various biological systems. Its capabilities span through the generation of lipid bilayers and micelles, the addition of various ions, and more. For comprehensive tutorials and practical demonstrations, users can visit the official CHARMM-GUI website [126].

## Appendix C

Most typical MD simulations are performed in explicit solvent using periodic boundary conditions, which allow for the estimation of concentration and avoid unphysical problems associated with interactions with the box sides. Figure A2 illustrates the differences between the all-atom and coarse-grained simulation boxes in the Amber and Gromacs packages. In Amber, a truncated octahedron periodic box is used to save computational time by omitting calculations for the water molecules placed in the corners of the box. In contrast, Gromacs, when coupled with the Martini 3 force field, uses a slightly more computationally expensive but simpler cubic box in terms of symmetry operations and further analysis.

In a typical simulation of a monomeric system, the user must ensure that the selected periodic boundary box is large enough to prevent contacts between the periodic images of the solute. To verify whether there are any contacts between periodic images (in other words, to check if the simulation box is sufficiently large), one can calculate the minimum-image distance (Figure A3). This distance is the minimum distance between any solute atoms, calculated only between solute atoms in different periodic images and not within the same periodic box. In our case, it is clear that there are no interactions between periodic images within the cut-off range (0.9 and 1.2 nm for the ff19SB and SIRAH models, respectively), and the lowest observed values are above 1.6 and 2.6 nm for the ff19SB and SIRAH models, respectively. These values are well above the threshold, indicating that the periodic box was sufficiently large.

Another approach to ensuring the proper size of the periodic boundary box is to measure D_max_, i.e., the maximum of distances between all pairs of solute atoms (within the same periodic box), and compare it with the box size (Figure A4 and Figure A5). Although simpler, as this approach calculates only distances within a single periodic box, it does not take into account the influence of the solute’s orientation in the simulation box on the possibility of forming inter-box contacts. Therefore, in some cases, even with D_max_ values comparable to the box size, no contacts between solute molecules may be observed. It should be noted that in our simulations, D_max_ values do not exceed 7 nm, which is clearly smaller than the simulation box side length of approximately 9 nm. Therefore, the simulation box is sufficiently large to ensure that the peptide does not interact with its periodic image during any of the simulations performed.

The last technical check of the simulations involves ensuring that they reach convergence and equilibrium [114]. This check is important, as it may indicate if the simulations span a sufficient timescale to capture interesting phenomena. However, there are no universal methods to ensure convergence and full equilibration (please see Appendix B for more details) [114]. Typically, convergence is assumed when structural properties (such as RMSD, R_g_, or D_max_) reach a plateau or when averages over various parts of the simulations are qualitatively equal. However, such behavior is not expected for IDPs, such as monomeric Aβ42, for which multiple transitions between various conformational states are expected instead. This behavior is clearly observed for Amber and Martini simulations (Figure A3, Figure A4 and Figure A5). In contrast, in SIRAH trajectories, the results are not very similar to each other, and the observed fluctuations are rather small, despite using the longest total computational time of 60 µs (Figure A3 and Figure A4). This suggests a possible overstabilization of certain Aβ42 conformations in the SIRAH force field rather than inadequate sampling.
