# Supplementary material for: A Practical Guide to All-Atom and Coarse-Grained Molecular Dynamics Simulations Using Amber and Gromacs: A Case Study of Disulfide-Bond Impact on the Intrinsically Disordered Amyloid Beta
Source: Int J Mol Sci. 2024 Jun 18;25(12):6698. doi: 10.3390/ijms25126698 (PMC11204378; doi:10.3390/ijms25126698)
Supplement: Supplementary file 1 [file ijms-25-06698-s001.zip › Scripts.zip!/Amber_all-atom_and_SIRAH/sirah_x2.2_20-08.amber/tutorial/SIRAH_FAQs.pdf]

SIRAH FAQs!

Tips and tricks to make your life easier

By Matias Machado
Mail any comment or suggestion to spantano@pasteur.edu.uy

The SIRAH force field is easy to use and we mean it! We put a lot of effort to make it plug-and-play and self-contained so for most cases it should work fine just out of the box. However, there are some user specific issues that need to be addressed...

General questions

What does SIRAH name stand for?

SIRAH is an acronym for "South-American Initiative for a Rapid and Accurate Hamiltonian", but it is also the name of an historically old grape very popular in Argentina and Uruguay.

In which MD engines is it possible to run the SIRAH force field?

We currently provide native ports for AMBER (14 or later) and GROMACS (4.5.5 or later). However, it could be easily implemented in other MD software as long as it supports the AMBER potential and setting out-off diagonal Lennard-Jones interactions.

What does the package version stand for?

The SIRAH packages are distributed as TAR files including a code to easily identify the version number, year and month of release (e.g. x2_19-03 stands for SIRAH 2.0 released on March 2019).

Do I need a PQR file for mapping to SIRAH?

No, you just need a structure containing the heavy atoms and the mapped polar hydrogens, charge and vdW records are not used at all. Indeed, the frequently absent protons can be added using any software you like (e.g. pdb4amber, H++) or you can even use structures which already have them from an atomistic MD, NMR or a high resolution X-ray.

Why do you suggest mapping from a PQR file?

Because processing a PDB structure through the PDB2PQR server has several advantages: 1) The server is able to build missing residue atoms provided there is enough information (i.e. more than just the CA atom). 2) The server not only add protons but it can predict and assign the protonation state of HIS, GLU, ASP and LYS according to the hydrogen network and the defined pH. 3) The server can use the best compatible naming scheme (AMBER) for mapping to SIRAH. That means not only protonation states but also CYS forming disulfide bonds are detected and renamed accordingly (CYX).

Which are the disadvantages of the PDB2PQR server?

The server can only handle and print residues or molecules for which it has parameters. Other residues will be removed and won't be used in pKa calculations and protonation state assignation. Examples of modified residues lacking parameters are: MSE (seleno MET), TPO (phosphorylated THY) and SEP (phosphorylated SER). A workaround for that issue is mutating the residues to their unmodified form before submitting the structure to the server. Be aware that blank lines in the input PDB file may be interpreted as an EOF (end-of-file), causing the server to stop reading the file. In that case, the output will depend on the parsed information up to that point. The server may also have problems to deal with big systems like entire viral particles, due to memory restrictions.

I appended the PDB coordinates of a ligand to the PQR file and VMD makes a mess with the connectivity, why?

This is just a visualization problem of VMD when interpreting the HETATM keyword within the PQR file format. To solve the problem rename the field HETATM to ATOM or force VMD to read the PQR file as a PDB by adding the flag *-pdb* before the PQR file on the command line, which is the same as choosing explicitly PDB format in the *Molecule File Browser* at the GUI interface.

Which are the main cautions for mapping protein structures directly from experimental or atomistic force fields?

The main concern is the name of protonable residues. Although we provide compatibility for naming schemes in PDB, AMBER, GMX, GROMOS, CHARMM and OPLS, there always may be some ambiguity in the residue naming, specially regarding protonation states, that may lead to a wrong mapping. For example, SIRAH Tools always maps the residue name “HIS” to a Histidine protonated at $N\varepsilon$ regardless the actual proton placement. To unambiguously distinguish each case use “HIE” or “HSE” names for $N\varepsilon$ protonation and “HID” or “HSD” names for $N\delta$ protonation. Be aware, there is no model for double protonated Histidine yet, so the default mapping is to a $N\varepsilon$ protonation. Similarly, protonated Glutamic and Aspartic acid residues must be named “GLH” and “ASH”, otherwise they will be treated as negatively charged residues. In addition, reduced and disulfide bonded Cysteines must be named “CYS” and “CYX”, respectively. These kind of situations need to be carefully checked by the users. In all cases the residues preserve their identity when mapping and back-mapping the structures. Hence, the total charge of the protein should be the same at atomistic and SIRAH level. You can always check the mappings at folder *tools/CGCONV/maps/sirah_prot.map* within the SIRAH package.

Is it possible to account for different protonation states and pH effects in SIRAH?

Yes, but only in the protein's model and for some residues.

Can SIRAH account for Post-Translational Modifications (PTMs)?

Yes! Starting from SIRAH 2.1 (Garay et al. [JCIM, **2019**]), there is out of the box support for the most common PTMs.

Can SIRAH account for disulfide bonds?

Yes, but the way to set them depends on the MD engine used to build and simulate the system (see AMBER and GROMACS specific questions).

Which protein termini residues are available for SIRAH proteins?

Both charged and neutral termini are supported. By default charged termini are used in all MD engines, see AMBER and GROMACS specific questions to learn how to set them neutral.

Which are the main cautions for mapping lipids?

Presently, lipids have no systematic nomenclature as aminoacids and nucleotides do. Hence, different conventions were adopted by force field developers to implement them in MD engines. Despite a universal solution is not possible, the SIRAH package provides a set of mapping files (MAPs) compatible with widely used force fields (Lipid11-17, GAFF, CHARMM 27/36, OPLS, GROMOS and Slipids) as implemented in different databases (AMBER, GROMACS, CHARMM-GUI, Lipidbook, MemBuilder, VMD and HTMD). By default no mapping is applied to lipids, hence users are requested to append the corresponding file from the available options at folder *tools/CGCONV/maps/*. Due to

possible nomenclature conflicts, users are advised to check and modify the MAPs as required. SIRAH supports residue-based and fragment-based topologies. In case of fragment-based topologies (the AMBER new standard), the order of fragments in the PDB file is relevant to the lipid constitution and identity, being tail(sn1)-head-tail(sn2) the expected format. Tails are connected to the head through the glycerol moiety, which is asymmetric, so swapping tail order changes the lipid nature. See AMBER and GROMACS questions for specific tips on each implementation.

Can I use MAP files to inter-convert between different lipid names or topology conventions?

No, the provided MAP files are meant to map a given atomistic model to its CG representation, so the MAP *tools/CGCONV/maps/amber_lipid.map* won't generate a fragment-based topology from a residue-based description.

I'm getting the following warning message when running cgconv.pl: "*Some residues were not found in MAP files: WAT*". Is it an error?

No, it is just fine, there is no CG mapping in SIRAH for atomistic water (WAT). However, a similar message may pop-up for other residues, in which case the user is required to check whether they are not supported in the force field or there is some issue in the mapping.

Why are DNA bead types very different from the original model?

Since SIRAH version x2_19-06, DNA bead types were renamed from the former work of Dans et al. [JCTC, **2010**, 6:1711] to prevent conflicts and overwriting all-atom force fields (e.g. ff14SB) when performing multiscale simulations. The OLD:NEW names are CX:D2, NW:D1, NX:D6, OY:M2, NZ:M3, OX:M4, OV:S2, NU:S3, NT:S4, NS:J2, NR:J1, OZ:J6.

Which integration time-step should I use in SIRAH simulations?

SIRAH was developed and validated to run with a time-step of 20 fs. Using a lower integration step is a waste of speed up!

Why did my simulation crash using a 20 fs time-step?

It may happens, particularly starting from low resolution structures, homology models or 'frankensteinian' systems, that bad contacts/conformations lead to abrupt energy/coordinate changes making the calculation crashes even at the equilibration step. The most common solution to that problem consists on running a few simulation steps (typically 500 to 1000 ps) at 2 fs and then switching to 20 fs.

Why do you recommend a two step minimization/equilibration protocol for proteins?

The coarse-grained nature of SIRAH provides some sensibility to the initial structure of the system. Occasionally, atomistic packing is required to properly describe a given conformation or structural motif (e.g. a binding pocket for an ion). Such context of interactions may not be correctly described at CG level due to granularity limitations. Hence, by first allowing side-chains to relax while fixing the backbone conformation greatly improves the structural stability of proteins by avoiding major distortions to secondary structure elements and the overall folding. Then the whole protein could be relaxed. This strategy also helps the proper hydration of the side-chains. The graduality at which the protein is released from the positional restraints may depend on the system, for most cases we found two steps are enough.

Why did my protein partially unfold during the simulation?

The are certainly many reasons for that, which range from the initial structure of the system to wrong setup of the MD options and intrinsic limitations of the SIRAH force field. Due to resign degrees of freedom and interactions by coarse-graining, the structure will always change to some extent. However, an issue to be aware is the presence of charged residues within the protein folding (e.g.: GLU222 in 1QYO or ASP320 in 3EHG). Such hydrophilic residues won't be pleased in a hydrophobic environment. Similarly, highly charged pockets without their binding ligands or ions may unfold disturbing the structure (e.g.: 1CFD). Depending on the system, using neutral species (e.g. GLH in case of GLU) may ameliorate these problems. Another problematic situation is the misplacing of WT4/ions inside the hidrophobic core at the solvation step. Despite erasing WT4 molecules up to 0.3 nm from the protein same molecules may eventually remain. These solvent molecules may make their way to the solution by disrupting the protein folding. The solution is removing the problematic solvent molecules. However, there are cases in which water or ions play important structural roles, but those cavities may be inaccessible or impossible to fill due to granularity limitations (e.g. 2M06 or 4XDJ). A possible way to overcome this limitation may be using restraints or local elastic networks to preserve the structural motif. Importantly, all mentioned cases need to be check before performing the simulations, as a very gently equilibration may not be enough to guaranty the stability of the protein.

***sirah_ss* assign some residues as coil, does it means they are unfold or randomly moving?**

Not necessarily, the strict definition of coil used by *sirah_ss* is "*not helix nor extended sheet*", which means a residue that can not satisfy either condition. Importantly, the secondary structure is assigned according to the Ramachandran and the hydrogen bond network. Particularly, the later is very sensitive to small fluctuation around the distance criteria used to define the interaction. Hence, transient coil states may be more likely to point the lost of hydrogen bonds in well folded proteins, rather that shifts in the conformational space.

Why did my membrane dramatically shrink in XY planes during the simulation?

We have observed that sometimes membranes may randomly experience a dramatic collapse in XY planes while forming a multi-lamellar-like structure in Z axis due to PBC conditions. This often happens at the very beginning of the production simulation (first 50 to 100 ns). So far, such behavior was seen while running many replicates of membrane tutorials for GROMACS and it was particularly notorious after versions 2016.6 and 2018.6. The new implemented equilibration protocols in GROMACS tutorials should have diminished those events, however they may eventually occur, in which case we recommend running the simulation again. Despite several reasons may be behind this phenomena, it is evident that membrane systems are very sensitive to initial conditions and should be treated gently.

Can I use advanced sampling strategies with SIRAH?

Yes! Check these examples from the literature: *Enantioselective Catalysis by Using Short, Structurally Defined DNA Hairpins as Scaffold for Hybrid Catalysts* [Chem.Eur.J, **2017**, 23:6004], *Fast Calculation of Protein–Protein Binding Free Energies Using Umbrella Sampling with a Coarse-Grained Model* [JCTC, **2018**, 14:991]. Indeed, MD engines don't distinguish SIRAH from atomistic system, so in principle you could apply any feature available in them. However, remember that SIRAH is a CG model so you should be careful in the validation and interpretation of the results. Hence, follow the recommendation of people who has already tested it or be the first in doing it!

Is SIRAH sensitive to electric fields?

Yes! See *Cues to Opening Mechanisms From in Silico Electric Field Excitation of Cx26 Hemichannel and in Vitro Mutagenesis Studies in HeLa Transfectans* [Front.Mol.Neurosci, **2018**, 11:170]. Electroporation was also showed in *Fat SIRAH: Coarse-Grained Phospholipids To Explore Membrane–Protein Dynamics* [JCTC, **2019**, 15:5674].

AMBER specific questions

Why can't I display files **.ncrst* with VMD?

AMBER restart files in NetCDF format are supported from VMD version 1.9.3.

How do I set disulfide bonds in AMBER?

First, Cysteine residues forming a disulfide bond must be named CYX in the atomistic structure and mapped to sX at SIRAH level. Check that the mapping was done OK or fix it by renaming the corresponding residues. Then define each disulfide bond explicitly in LEAP using the command *bond* (e.g.: *bond unit.ri.BSG unit.rj.BSG*). Where *ri* and *rj* correspond to the residue indexes in the topology file, which on the contrary to the biological sequence in the PDB file, they always start from 1. You can try the command *pdb4amber* to get those residue indexes from the atomistic structure.

How do I set neutral protein termini in AMBER?

Neutral terminals can be set by renaming the corresponding residues from s[*code*] to a[*code*] (Nt-acetylated) or m[*code*] (Ct-amidated) after mapping, where [*code*] is the root residue name in SIRAH. For example, to set a neutral N-terminal Histidine protonated at Nε rename it from “sHe” to “aHe”.

Is it possible to use the fragment-based framework of AMBER for SIRAH lipids in AMBER?

Yes! SIRAH provides native ports for relevant lipid heads and tails, and the mapping file *tools/CGCONV/maps/amber_lipid.map* to generate the CG models from the atomistic coordinates. As in Lipid11-17 force fields, the order of fragments in the PDB file is relevant to the lipid constitution and identity, being tail(sn1)-head-tail(sn2) the expected format. Notice, tails are connected to the head through the glycerol moiety, which is asymmetric, so swapping the tail order changes the lipid nature. Each lipid unit must be delimited by TER statements to correctly connect the head and tail fragments.

Why do I need to scale down the WT4 radii during the solvation of the computational box?

The solvation strategy of Leap consists on filling the computational box with clusters of pre-equilibrated solvent molecules and then removing the overlapping ones with the solute. Although fast, this strategy leaves interstices around the solute solvation shell which are fixed in subsequent simulation steps. In CG systems this issue becomes more pronounced due to the granularity of the particles. In practice, using the actual bead’s sizes of SIRAH produces poorly solvated proteins, because no solvent molecules are present up to 0.5 nm away. Such condition has dramatic effects during the energy minimization and equilibration as charged amino acids at the surface of the protein interact alike *in vacuum* at initial steps leading to important structural distortions. A way to improve the initial solute solvation is allowing for small overlaps with solvent beads when generating the solvation box, which are then relaxed during the energy minimization. The scaling factor of 0.7 arises from considering the radius of a WT4 bead and the most represented solute bead, which coincidentally is 0.21 nm, and setting the effective overlapping radius so that (0.21 + 0.21) * 0.7 ~ 0.3 nm. This solvation scheme was empirically tested to work.

Why do you recommend equilibrate the system in NPT ensemble?

After solvating a system, Leap deletes all solvent molecules crossing the boundaries of the computational box. As a result, the solvent density at the interface of periodic images is reduced. This defect is fixed in subsequent simulation steps. In CG systems this issue becomes very pronounced due to the granularity of the particles. Hence, notorious vacuum bubbles may appear during initial NVT simulations. To avoid such behavior, we recommend equilibrate the system in NPT to adjust the box size to the actual solvent density.

Why is it critical to correct the size of the simulation box in a membrane system before MD?

To add solvent, Leap defines the size of the simulation box according to the vdW radius of the farthest atom at each coordinate of the system. Such criteria generates bigger boxes than required, leaving important void spaces at boundaries, which break the continuity of the membrane along periodic images, despite using a zero distance to box sides. In case of using pre-equilibrated systems this issue may be more pronounced, as Leap can't handle situations where lipid molecules may split through periodic images of the box. As a consequence, the equilibration process may became very tricky to avoid the system explosion, pore formations or other artefactual effects. A simple solution implies post-processing the coordinates of the output system (e.g. by using *cpptraj*) to fix the system boundaries. When using PACKMOL to both generate the membrane and solvate the system, all molecules remain whole within the box, as no PBC is considered. In that situation, post-processing may be avoided by using the following command in Leap to set the box according to atoms' centers: *setbox unit centers 0*.

How do I check the topology for Lennard-Jones interactions out-off the combination rules?

If the system contains special Lennard-Jones (LJ) interactions then the following message should be read in all output files (**.out*) of *pmemd* and *pmemd.cuda* :

```
| INFO: Off Diagonal (NBFIX) LJ terms found in prmtop.
|       The prmtop file has been modified to support atom
|       type based pairwise Lennard-Jones
```

Alternatively, you can use the command *printLJMatrix @%** in Parmed.

Why is it important to set *chngmask=0* keyword at *&ewald* section in the input files?

This is just required for running SIRAH simulation with SANDER. By default SANDER re-builds the 1-4 exclusion list from the *prmtop* and store it in memory. However it fails to do it properly in case of triangular bonds (e.g. in WT4), causing the calculation to abort with the error message *EXTRA POINTS: nnb too small!*. The *chngmask=0* keyword at *&ewald* section avoids re-building the list, which is correctly done by LEAP when creating the *prmtop*. Important, PMEMD (CPU or GPU) codes do not have such issue, so setting *chngmask=0* is not mandatory.

GROMACS specific questions

How do I set disulfide bonds in GROMACS?

Disulfide bonds are automatically detected in the structure by *pdb2gmx* if the file *specbond.dat* is present at the same folder where the command is executed. However some caution words must be said: Disulfide bonds can only be detected within the same molecular unit, so for an inter-chain disulfide bond to be correctly set both protein chains must be merge into a single topology. There is also a distance criteria specified at *specbond.dat* that must be satisfied within a +/-10% cut-off. Any

bond outside that threshold won't be recognized. This last issue may be frequent in low resolution structures (e.g. cryo-EM structures).

How can I fix long distance disulfide bonds?

These are two possible solution from [GROMACS How-to]: 1) Modify the file *specbond.dat* so that unbound atoms meet the distance criteria. Warning! This solution may solve the problem for some bonds but translate the issue to others. 2) Minimize the system using distance restrains between unbound atoms to force their proximity and then rebuild the topology with *pdb2gmx*.

How do I set neutral protein termini in GROMACS?

Just add the *-ter* flag to the *pdb2gmx* command line and set them interactively when prompted. Notice, GROMACS does not rename terminal residues.

Why do I need to remove WT4 molecules within 0.3 nm of the protein?

The solvation strategy of GROMACS consists on filling the computational box with clusters of pre-equilibrated solvent molecules and then removing the overlapping ones with others. Overlaps are defined according to a default radii of 0.105 nm for atoms not present in the van der Waals database (*vdwradii.dat*). Due to the granularity of the CG model, solvent molecules are placed by default in close contact with the protein, sometimes inside the folding, leading to clashes or structural deformations during simulations. On the other hand, using the actual bead's sizes from the *vdwradii.dat*, which is provided in the SIRAH package, produces poorly solvated proteins, because no solvent molecules are present up to 0.5 nm away. Such condition has dramatic effects during the energy minimization and equilibration as charged amino acids at the surface of the protein interact alike *in vacuum* at initial steps leading to important structural distortions. In addition, the solvent density is compromised by solvent-solvent overlaps. As a solution, we empirically found that removing WT4 molecules within 0.3 nm from the solute after a default solvation, was a good trade off between side chain hydration, structural stability and solvent density.

Is it possible to use the fragment-based framework of AMBER for SIRAH lipids in GROMACS?

Yes! SIRAH provides native ports for relevant lipid heads and tails, and the mapping file *tools/CGCONV/maps/amber_lipid.map* to generate the CG models from the atomistic coordinates. As in AMBER, the order of fragments in the PDB file is relevant to the lipid constitution and identity, being tail(sn1)-head-tail(sn2) the expected format. Notice, tails are connected to the head through the glycerol moiety, which is asymmetric, so swapping tail order changes the lipid nature. Due to our implementation of fragments in GROMACS, we recommend removing TER statements delimiting lipids before generating the topology with *pdb2gmx* to avoid creating hundreds of individual topology files for each molecule. Ideally a single TER statement splitting leaflets is enough to easily handle the membrane.

Why is GROMACS not able to correctly define Protein, DNA or other groups?

That is probably because the file *residuetype.dat* is not present in the same folder where you are executing *pdb2gmx* or *make_ndx* commands.

Is it OK to get missing atom errors when running *pdb2gmx* or *grompp* commands on a SIRAH system?

No, that is a big error which probably trace back to the mapping step. Be aware that the script *cgconv.pl* does not check or add missing atoms, so inspect your atomistic and mapped structures to be sure that all mapped atoms are present in your former structure. Avoid using the flag *-missing* in

pdb2gmx and don't carry on the simulation until the problem is solved.

I can not minimize my system, what is the problem?

The most likely answer is you have big clashes (atom overlaps) on your system. If you don't fix them any subsequent simulation will crash. You are likely to have these issues in 'frankensteinian' systems. You can use the selection 'serial #' in VMD (where '#' is the atom number presenting the maximum force in the GROMACS log file) to visually identify the problematic contact and then remove or move the corresponding molecule.

Why do you recommend to equilibrate in NVT ensemble?

Due to technical reasons, GROMACS may fail to correctly apply positional restraints in NPT ensemble. As documented for mdp option *refcoord-scaling*, by default when using pressure coupling the center of mass of the reference coordinates is scaled without considering PBC conditions. As a result, systems in which restrained molecules are split though PBC images along the simulation box, may suffer from artifactual forces. Such issues are likely to arise in large systems (e.g. viral particles).
