# Supplementary material for: A Practical Guide to All-Atom and Coarse-Grained Molecular Dynamics Simulations Using Amber and Gromacs: A Case Study of Disulfide-Bond Impact on the Intrinsically Disordered Amyloid Beta
Source: Int J Mol Sci. 2024 Jun 18;25(12):6698. doi: 10.3390/ijms25126698 (PMC11204378; doi:10.3390/ijms25126698)
Supplement: Supplementary file 1 [file ijms-25-06698-s001.zip › Scripts.zip!/Amber_all-atom_and_SIRAH/sirah_x2.2_20-08.amber/tutorial/Tutorial_2_sirah4amb.pdf]

Tutorial 2. SIRAH force field in AMBER

Simulation of a coarse grained DNA molecule in explicit solvent

By Matias Machado
Mail any comment or suggestion to *spantano@pasteur.edu.uy*

This tutorial shows how to use the SIRAH force field to perform a coarse grained (CG) simulation of a double stranded DNA embedded in explicit solvent (called WatFour, WT4). The main references for this tutorial are: Darré et al. *WAT4?* [JCTC, **2010**, 6:3793], Machado et al. *SIRAH Tools* [Bioinformatics, **2017**, 32:1568]. We strongly advise you to read these articles before starting the tutorial.

Required Software
AMBER 16 and AMBER Tools 16 or later versions properly installed and running in your computer. The molecular visualization program VMD 1.9.3 or later (freely available at *www.ks.uiuc.edu/Research/vmd*).

Prior knowledge
How to perform a standard atomistic molecular dynamic simulation with AMBER and basic usage of VMD. If you are not familiar with DNA stuff we strongly recommend you to first perform the AMBER tutorial on DNA (*http://ambermd.org/tutorials/basic/tutorial1*).

Hands on
0) Download the file *sirah_[version].amber.tgz* from *www.sirahff.com* and uncompress it into your working directory. Notice: *[version]* should be replaced with the actual package version e.g.: x2_18-09

```
tar -xzvf sirah_[version].amber.tgz
```

You will get a folder *sirah_[version].amber/* containing the force field definition, the SIRAH Tools in *sirah_[version].amber/tools/*, molecular structures to build up systems in *sirah_[version].amber/PDB/,* frequently asked questions in *sirah_[version].amber/tutorial/SIRAH_FAQs.pdf* and the required material to perform the tutorial in *sirah_[version].amber/tutorial/2/*

Make a new folder for this tutorial in your working directory:

```
mkdir tutorial2; cd tutorial2
```

Create the following symbolic link in the folder *tutorial2*:

```
ln -s ../sirah_[version].amber sirah.amber
```

1) Map the atomistic structure of a 20-mer DNA to its CG representation:

```
./sirah.amber/tools/CGCONV/cgconv.pl\
 -i ./sirah.amber/tutorial/2/dna.pdb\
 -o dna_cg.pdb
```

The input file *dna.pdb* contains all the heavy atoms composing the DNA molecule, while the output *dna_cg.pdb* preserves a few of them. Please check both PDB structures using VMD:

```
vmd -m ./sirah.amber/tutorial/2/dna.pdb dna_cg.pdb
```

Notice: This is the basic usage of the script *cgconv.pl*, you can learn other capabilities from its help:

```
./sirah.amber/tools/CGCONV/cgconv.pl -h
```

From now on it is just normal AMBER stuff!

2) Use a text editor to create the file *gensystem.leap* including the following lines:

```
# Load SIRAH force field
  addPath ./sirah.amber
  source leaprc.sirah

# Load model
  dna = loadpdb dna_cg.pdb

# Info on system charge
  charge dna

# Add solvent, counterions and 0.15M NaCl
# Tuned solute-solvent closeness for best hydration
  solvateoct  dna WT4BOX 20 0.7
  addionsrand dna NaW 88 ClW 50

# Save Parms
  saveAmberParmNetcdf dna dna_cg.prmtop dna_cg.ncrst

# EXIT
  quit
```

Notice: The available ionic species in SIRAH force field are: Na+ (NaW), K+ (KW) and Cl- (ClW). One ion pair (e.g. NaW-ClW) each 34 WT4 molecules renders a salt concentration of ~0.15M (see Appendix 1). Counterions were added according to Machado et al. *SPLIT* [JCTC, **2020**].

3) Run the LEAP application to generate the molecular topology and initial coordinate files:

```
tleap -f gensystem.leap
```

Notice: Warning messages about long, triangular or square bonds in *leap.log* file are fine and expected due to the CG topology.

This should create a topology file *dna_cg.prmtop* and a coordinate file *dna_cg.ncrst*.

Use VMD to check how the CG model looks like:

```
vmd dna_cg.prmtop dna_cg.ncrst -e ./sirah.amber/tools/sirah_vmdtk.tcl
```

Notice: VMD assigns default radius to unknown atom types, the script *sirah_vmdtk.tcl* sets the right ones. It also provides a kit of useful selection macros, coloring methods and backmapping utilities. Use the command *sirah_help* in the Tcl/Tk console of VMD to access the manual pages.

4) Run the simulation

Make a new folder for the run:

```
mkdir -p run; cd run
```

In the course of long MD simulations the capping residues may eventually separate, this effect is called helix fraying. To avoid such behavior create a symbolic link to the file *dna_cg.RST*, which contains the definition of Watson-Crick restraints for the capping base pairs of this CG DNA:

```
ln -s ../sirah.amber/tutorial/2/SANDER/dna_cg.RST
```

Notice: The file *dna_cg.RST* can only be read by SANDER, PMEMD reads a different restrain format.

The folder *sirah.amber/tutorial/2/SANDER/* contains typical input files for energy minimization (*em_WT4.in*), equilibration (*eq_WT4.in*) and production (*md_WT4.in*) runs. Please check carefully the input flags therein, in particular the definition of flag chngmask=0 at &ewald section is mandatory.

Energy Minimization:

```
sander -O\
 -i ../sirah.amber/tutorial/2/SANDER/em_WT4.in\
 -p ../dna_cg.prmtop\
 -c ../dna_cg.ncrst\
 -o    dna_cg_em.out\
 -r    dna_cg_em.ncrst &
```

Equilibration (NPT):

```
sander -O\
 -i   ../sirah.amber/tutorial/2/SANDER/eq_WT4.in\
 -p   ../dna_cg.prmtop\
 -c      dna_cg_em.ncrst\
 -ref    dna_cg_em.ncrst\
 -o      dna_cg_eq.out\
 -r      dna_cg_eq.ncrst\
 -x      dna_cg_eq.nc &
```

Production (100ns):

```
sander -O\
 -i ../sirah.amber/tutorial/2/SANDER/md_WT4.in\
 -p ../dna_cg.prmtop\
 -c    dna_cg_eq.ncrst\
 -o    dna_cg_md.out\
 -r    dna_cg_md.ncrst\
 -x    dna_cg_md.nc &
```

Notice: You can find example input files for CPU and GPU versions of *pmemd* at folder *PMEMD/* within *sirah.amber/tutorial/2/*

That's it! Now you can analyze the trajectory.

Process the output trajectory to account for the Periodic Boundary Conditions (PBC):

```
echo -e "autoimage\ngo\nquit\n" |
cpptraj\
 -p ../dna_cg.prmtop\
 -y dna_cg_md.nc\
 -x dna_cg_md_pbc.nc\
 --interactive
```

Load the processed trajectory in VMD:

```
vmd ../dna_cg.prmtop ../dna_cg.ncrst dna_cg_md.nc\
 -e ../sirah.amber/tools/sirah_vmdtk.tcl
```

Appendix 1: Calculating ionic concentrations

$\rho_{WT4} = \rho_{H2O} = 1000$ g/L
$MW_{H2O} = 18$ g/mol
1 WT4 ~ 11 H_2O

$$M=\frac{mol}{V} \quad ; \quad n=mol\,N_A \quad ; \quad \rho=\frac{m}{V} \quad ; \quad m=mol\,MW$$

$$V=\frac{m}{\rho}=\frac{mol\,MW_{H_2O}}{\rho}=\frac{n_{H_2O}\,MW_{H_2O}}{N_A\,\rho} \quad ; \quad M=\frac{mol}{V}=\frac{n_{ion}}{N_A V}=\frac{n_{ion}}{N_A}\frac{N_A\,\rho}{n_{H_2O}MW_{H_2O}}=\frac{n_{ion}1000}{n_{WT4}(11)(18)}\sim 5\frac{n_{ion}}{n_{WT4}}$$

Number of WT4 molecules per ion at 0.15M: $n_{WT4}=5\frac{n_{ion}}{M}=\frac{5(1)}{0.15}\sim 34$
