# Supplementary material for: A Practical Guide to All-Atom and Coarse-Grained Molecular Dynamics Simulations Using Amber and Gromacs: A Case Study of Disulfide-Bond Impact on the Intrinsically Disordered Amyloid Beta
Source: Int J Mol Sci. 2024 Jun 18;25(12):6698. doi: 10.3390/ijms25126698 (PMC11204378; doi:10.3390/ijms25126698)
Supplement: Supplementary file 1 [file ijms-25-06698-s001.zip › Scripts.zip!/Amber_all-atom_and_SIRAH/sirah_x2.2_20-08.amber/tutorial/Tutorial_3_sirah4amb.pdf]

Tutorial 3. SIRAH force field in AMBER

Multiscale simulation of TBP bound to HIV promoter in implicit solvent

By Matias Machado
Mail any comment or suggestion to *spantano@pasteur.edu.uy*

This tutorial shows how to apply a multiscale representation of DNA, which is available in the SIRAH force field, to study protein-DNA interactions. In this approach, the protein and its binding region are treated with an atomistic force field, while the contextual DNA is represented at coarse-grained (CG) level. The simulated system is composed of the human TATA binding protein (TBP, PDB: 1C9B) bounded to the TATA box at the promoter region (-64 to +13) of the Human Immunodeficiency Virus type 1 (HIV-1, GenBank: K03455, Figure 1). Solvent effects are considered implicitly using the Generalized Born model (GB).

The main references for this tutorial are: Machado et al. *All-atoms/CG DNA* [PCCP, **2011**, 13:18134], Machado et al. *SIRAH Tools* [Bioinformatics, **2017**, 32:1568]. We strongly advise you to read these articles before starting the tutorial.

```
   -60       -50       -40       -30       -20       -10       +1       +10
....|.........|.........|.........|.........|.........|.........|........|...
GCGGGACTGGGGAGTGGCGAGCCCTCAGATCCTGCATATAAGCAGCTGCTTTTTGCCTGTACTGGGTCTCTCTGGTT
```

Figure 1 | Promoter region of HIV-1. The TATA box is highlighted in orange, while base pairs at 0.7nm of TBP are colored in yellow.

Required Software

AMBER 16 and AMBER Tools 16 or later versions properly installed and running in your computer. The molecular visualization program VMD 1.9.3 or later version (freely available at *www.ks.uiuc.edu/Research/vmd*).

Prior knowledge

How to perform a standard atomistic molecular dynamic simulation with AMBER and basic usage of VMD. If you are not familiar with DNA stuff we strongly recommend you to first perform the AMBER tutorial on DNA (*http://ambermd.org/tutorials/basic/tutorial1*).

Hands on

0) Download the file *sirah_[version].amber.tgz* from *www.sirahff.com* and uncompress it into your working directory. Notice: *[version]* should be replaced with the actual package version e.g.: x2_18-09

```
tar -xzvf sirah_[version].amber.tgz
```

You will get a folder *sirah_[version].amber/* containing the force field definition, the SIRAH Tools in *sirah_[version].amber/tools/*, molecular structures to build up systems in *sirah_[version].amber/PDB/,* frequently asked questions in *sirah_[version].amber/tutorial/SIRAH_FAQs.pdf* and the required material to perform the tutorial in *sirah_[version].amber/tutorial/3/*

Make a new folder for this tutorial:

```
mkdir tutorial3; cd tutorial3
```

1) Map only the atomistic structure of nucleotides outside the TATA box of the HIV-1 promoter to CG:

```
./sirah.amber/tools/CGCONV/cgconv.pl\
 -i ./sirah.amber/tutorial/3/tbphiv.pdb\
 -R 1-32,45-110,123-154 \
 -o dna_hyb.pdb
```

The input file *tbphiv.pdb* contains all the heavy atoms composing the protein-DNA system. Mapped residues are selected through option -R. The selection considers a buffer of two base pairs at each side of the TATA box (Figure 1). The atomistic/CG interface must always be a step of B form DNA. The resulting coordinates are saved in the output *dna_hyb.pdb*. Please check both PDB structures in VMD:

```
vmd -m ./sirah.amber/tutorial/3/tbphiv.pdb dna_hyb.pdb
```

Notice: This is an advanced usage of the script *cgconv.pl*, you can learn more about it from its help:

```
./sirah.amber/tools/CGCONV/cgconv.pl -h
```

From now on it is just normal AMBER stuff!

2) Use a text editor to create the file *gensystem.leap* including the following lines:

```
# Load AMBER force field (parm14SB/parmbsc0)
  source oldff/leaprc.ff14SB

# Load SIRAH force field
  addPath ./sirah.amber
  source leaprc.sirah

# Load model
  dna = loadpdb dna_hyb.pdb

# Save Parms
  saveAmberParmNetcdf dna dna_hyb.prmtop dna_hyb.ncrst

# EXIT
  quit
```

Notice: According to AMBER version *10*, *11* or *12 (14)* the source file for parm99SB/bsc0 is *leaprc.ff99bsc0*, *leaprc.ff10* or *leaprc.ff12SB* respectively.

3) Run the LEAP application to generate the molecular topology and initial coordinate files:

```
tleap -f gensystem.leap
```

Notice: Warning messages about long, triangular or square bonds in *leap.log* file are fine and expected due to the CG topology of some residues.

This should create a topology file *dna_hyb.prmtop* and a coordinate file *dna_hyb.ncrst*.

Use VMD to check how the multiscale model looks like:

```
vmd dna_hyb.prmtop dna_hyb.ncrst -e ./sirah.amber/tools/sirah_vmdtk.tcl
```

Notice: VMD assigns default radius to unknown atom types, the script *sirah_vmdtk.tcl* sets the right ones. It also provides a kit of useful selection macros, coloring methods and backmapping utilities.

Use the command *sirah_help* in the Tcl/Tk console of VMD to access the manual pages.

4) Run the simulation

Make a new folder for the run:

```
mkdir -p run; cd run
```

In the course of long MD simulations the capping residues may eventually separate, this effect is called helix fraying. To avoid such behavior create a symbolic link to the file *dna_hyb.RST*, which contains the definition of Watson-Crick restraints for the capping base pairs of this CG DNA:

```
ln -s ../sirah.amber/tutorial/3/SANDER/dna_hyb.RST
```

Notice: The file *dna_hyb.RST* can only be read by SANDER, PMEMD reads a different restrain format.

The folder *sirah.amber/tutorial/3/SANDER* contains typical input files for energy minimization (*em_HYB.in*), equilibration (*eq_HYB.in*) and production (*md_HYB.in*) runs. Please check carefully the input flags therein.

Notice: This simulation is very time consuming owing to the system's size, so try a parallel implementation of AMBER.

Energy Minimization:

```
sander -O\
 -i ../sirah.amber/tutorial/3/SANDER/em_HYB.in\
 -p ../dna_hyb.prmtop\
 -c ../dna_hyb.ncrst\
 -o    dna_hyb_em.out\
 -r    dna_hyb_em.ncrst &
```

Equilibration:

```
sander -O\
 -i ../sirah.amber/tutorial/3/SANDER/eq_HYB.in\
 -p ../dna_hyb.prmtop\
 -c    dna_hyb_em.ncrst\
 -o    dna_hyb_eq.out\
 -r    dna_hyb_eq.ncrst\
 -x    dna_hyb_eq.nc &
```

Production (10ns):

```
sander -O\
 -i ../sirah.amber/tutorial/3/SANDER/md_HYB.in\
 -p ../dna_hyb.prmtop\
 -c    dna_hyb_eq.ncrst\
 -o    dna_hyb_md.out\
 -r    dna_hyb_md.ncrst\
 -x    dna_hyb_md.nc &
```

Notice: You can find example input files for CPU and GPU versions of *pmemd* at folders *PMEMD.CPU/* and *PMEMD.GPU/* within *sirah.amber/tutorial/3/*

That’s it! Now you can check the simulation using VMD:

```
vmd ../dna_hyb.prmtop ../dna_hyb.ncrst dna_hyb_md.nc\
 -e ../sirah.amber/tools/sirah_vmdtk.tcl
```
