# Supplementary material for: A Practical Guide to All-Atom and Coarse-Grained Molecular Dynamics Simulations Using Amber and Gromacs: A Case Study of Disulfide-Bond Impact on the Intrinsically Disordered Amyloid Beta
Source: Int J Mol Sci. 2024 Jun 18;25(12):6698. doi: 10.3390/ijms25126698 (PMC11204378; doi:10.3390/ijms25126698)
Supplement: Supplementary file 1 [file ijms-25-06698-s001.zip › Scripts.zip!/Amber_all-atom_and_SIRAH/sirah_x2.2_20-08.amber/tutorial/Tutorial_4_sirah4amb.pdf]

Tutorial 4. SIRAH force field in AMBER

Simulations of closed circular DNA in implicit solvent

By Matias Machado
Mail any comment or suggestion to *spantano@pasteur.edu.uy*

This tutorial shows how to use the SIRAH force field to perform a coarse grained (CG) simulation of a closed circular DNA using the Generalized Born model (GB) for implicit solvent. The main references for this tutorial are: Dans et al. *SIRAH DNA* [JCTC, **2010**, 6:1711] (latest parameters are reported in: Darré et al. *WAT4?* [JCTC, **2010**, 6:3793]), Machado et al. *SIRAH Tools* [Bioinformatics, **2017**, 32:1568]. We strongly advise you to read these articles before starting the tutorial.

Required Software
AMBER 16 and AMBER Tools 16 or later versions properly installed and running in your computer. The molecular visualization program VMD 1.9.3 or later version (freely available at *www.ks.uiuc.edu/Research/vmd*).

Prior knowledge
How to perform a standard atomistic molecular dynamic simulation with AMBER and basic usage of VMD. If you are not familiar with DNA stuff we strongly recommend you to first perform the AMBER tutorial on DNA (*http://ambermd.org/tutorials/basic/tutorial1*).

Hands on
0) Download the file *sirah_[version].amber.tgz* from *www.sirahff.com* and uncompress it into your working directory. Notice: *[version]* should be replaced with the actual package version e.g.: x2_18-09

```
tar -xzvf sirah_[version].amber.tgz
```

You will get a folder *sirah_[version].amber/* containing the force field definition, the SIRAH Tools in *sirah_[version].amber/tools/*, molecular structures to build up systems in *sirah_[version].amber/PDB/,* frequently asked questions in *sirah_[version].amber/tutorial/SIRAH_FAQs.pdf* and the required material to perform the tutorial in *sirah_[version].amber/tutorial/4/*

Make a new folder for this tutorial in your working directory:

```
mkdir tutorial4; cd tutorial4
```

Create the following symbolic link in the folder *tutorial4*:

```
ln -s ../sirah_[version].amber sirah.amber
```

1) Map the atomistic structure of the closed circular DNA to its CG representation:

```
./sirah.amber/tools/CGCONV/cgconv.pl -i ./sirah.amber/tutorial/4/ccdna.pdb |
sed -e 's/DCX A   1/CW5 A   1/'\
    -e 's/DCX B   1/CW5 B   1/'\
    -e 's/DGX A 100/GW3 A 100/'\
    -e 's/DGX B 100/GW3 B 100/'\
> ccdna_cg.pdb
```

Notice: 5' and 3' end residues mast be renamed to AW5, TW5, GW5 or CW5 and AW3, TW3, GW3 or CW3 to represent the corresponding Adenine, Thymine, Guanine or Cytosine extremes in a closed circular DNA.

Notice: This is the basic usage of the script *cgconv.pl*, you can learn other capabilities from its help:

```
./sirah.amber/tools/CGCONV/cgconv.pl -h
```

The input file *ccdna.pdb* contains all the heavy atoms composing the DNA molecule, while the output *ccdna_cg.pdb* preserves a few of them. Please check both PDB structures using VMD:

```
vmd -m ./sirah.amber/tutorial/4/ccdna.pdb ccdna_cg.pdb
```

From now on it is just normal AMBER stuff!

2) Use a text editor to create the file *gensystem.leap* including the following lines:

```
# Load SIRAH force field
  addPath ./sirah.amber
  source leaprc.sirah

# Load model
  dna = loadpdb ccdna_cg.pdb

# Make a covalent closed circular DNA
  bond dna.1.PX   dna.100.C1X
  bond dna.101.PX dna.200.C1X

# Save Parms
  saveAmberParmNetcdf dna ccdna_cg.prmtop ccdna_cg.ncrst

# EXIT
  quit
```

3) Run the LEAP application to generate the molecular topology and initial coordinate files:

```
tleap -f gensystem.leap
```

Notice: Warning messages about long, triangular or square bonds in *leap.log* file are fine and expected due to the CG topology.

This should create a topology file *ccdna_cg.prmtop* and a coordinate file *ccdna_cg.ncrst*.

Use VMD to check how the CG model looks like:

```
vmd ccdna_cg.prmtop ccdna_cg.ncrst -e ./sirah.amber/tools/sirah_vmdtk.tcl
```

Notice: VMD assigns default radius to unknown atom types, the script *sirah_vmdtk.tcl* sets the right ones. It also provides a kit of useful selection macros, coloring methods and backmapping utilities. Use the command *sirah_help* in the Tcl/Tk console of VMD to access the manual pages.

4) Run the simulation

Make a new folder for the run:

```
mkdir -p run; cd run
```

The folder *sirah.amber/tutorial/4/CPU* contains typical input files for energy minimization (*em_GB.in*), equilibration (*eq_GB.in*) and production (*md_GB.in*) runs. Please check carefully the input flags therein.

Energy Minimization:

```
sander -O\
 -i ../sirah.amber/tutorial/4/CPU/em_GB.in\
 -p ../ccdna_cg.prmtop\
 -c ../ccdna_cg.ncrst\
 -o    ccdna_cg_em.out\
 -r    ccdna_cg_em.ncrst &
```

Equilibration:

```
sander -O\
 -i ../sirah.amber/tutorial/4/CPU/eq_GB.in\
 -p ../ccdna_cg.prmtop\
 -c    ccdna_cg_em.ncrst\
 -o    ccdna_cg_eq.out\
 -r    ccdna_cg_eq.ncrst\
 -x    ccdna_cg_eq.nc &
```

Production (100ns):

```
sander -O\
 -i ../sirah.amber/tutorial/4/CPU/md_GB.in\
 -p ../ccdna_cg.prmtop\
 -c    ccdna_cg_eq.ncrst\
 -o    ccdna_cg_md.out\
 -r    ccdna_cg_md.ncrst\
 -x    ccdna_cg_md.nc &
```

Notice: You can find example input files for CPU and GPU versions of *pmemd* at folders *CPU/* and *GPU/* within *sirah.amber/tutorial/4/*

That's it! Now you can check the simulation using VMD:

```
vmd ../ccdna_cg.prmtop ../ccdna_cg.ncrst ccdna_cg_md.nc\
 -e ../sirah.amber/tools/sirah_vmdtk.tcl
```
