# Supplementary material for: A Practical Guide to All-Atom and Coarse-Grained Molecular Dynamics Simulations Using Amber and Gromacs: A Case Study of Disulfide-Bond Impact on the Intrinsically Disordered Amyloid Beta
Source: Int J Mol Sci. 2024 Jun 18;25(12):6698. doi: 10.3390/ijms25126698 (PMC11204378; doi:10.3390/ijms25126698)
Supplement: Supplementary file 1 [file ijms-25-06698-s001.zip › Scripts.zip!/Amber_all-atom_and_SIRAH/sirah_x2.2_20-08.amber/tutorial/Tutorial_5_sirah4amb.pdf]

Tutorial 5. SIRAH force field in AMBER

Simulation of coarse grained proteins in explicit solvent

By Matias Machado
Mail any comment or suggestion to *spantano@pasteur.edu.uy*

This tutorial shows how to use the SIRAH force field to perform a coarse grained (CG) simulation of a protein in explicit solvent (called WatFour, WT4) in four simple steps: 1 download; 2 map; 3 solvate and; 4 run. The main references for this tutorial are: Darré et al. *WAT4?* [JCTC, **2010**, 6:3793], Machado et al. *SIRAH 2.0* [JCTC, **2019**, 15:2719], Machado et al. *SIRAH Tools* [Bioinformatics, **2017**, 32:1568]. We strongly advise you to read these articles before starting the tutorial.

Required Software
AMBER 16 and AMBER Tools 16 or later versions properly installed in your computer. The molecular visualization program VMD 1.9.3 or later (freely available at *www.ks.uiuc.edu/Research/vmd*). The plotting software Grace (*plasma-gate.weizmann.ac.il/Grace*) and the R statistical package (*www.r-project.org*).

Prior knowledge
How to perform a standard atomistic molecular dynamic simulation with AMBER.

Hands on
0) Download the file *sirah_[version].amber.tgz* from *www.sirahff.com* and uncompress it into your working directory. Notice: *[version]* should be replaced with the actual package version e.g.: x2_18-09

```
tar -xzvf sirah_[version].amber.tgz
```

You will get a folder *sirah_[version].amber/* containing the force field definition, the SIRAH Tools in *sirah_[version].amber/tools/*, molecular structures to build up systems in *sirah_[version].amber/PDB/,* frequently asked questions in *sirah_[version].amber/tutorial/SIRAH_FAQs.pdf* and the required material to perform the tutorial in *sirah_[version].amber/tutorial/5/*

Make a new folder for this tutorial in your working directory:

```
mkdir tutorial5; cd tutorial5
```

Create the following symbolic link in the folder *tutorial5*:

```
ln -s ../sirah_[version].amber sirah.amber
```

1) Map the protonated atomistic structure of protein 1CRN to its CG representation:

```
./sirah.amber/tools/CGCONV/cgconv.pl\
 -i ./sirah.amber/tutorial/5/1CRN.pqr\
 -o 1CRN_cg.pdb
```

Notice: The mapping to CG requires the correct protonation state of each residue at a given pH. We recommend using the PDB2PQR server (*http://nbcr-222.ucsd.edu/pdb2pqr*) and choosing the output naming scheme of AMBER for best compatibility. Be aware that modified residues lacking parameters such as: MSE (seleno MET), TPO (phosphorylated THY), SEP (phosphorylated SER) or others are deleted from the PQR file by the server. In that case, mutate the residues to their unmodified form before submitting the structure to the server.

Notice: Pay attention to residue names when mapping structures from other atomistic force fields or experimental structures. Although we provide compatibility for naming schemes in PDB, GMX, GROMOS, CHARMM and OPLS, there always may be some ambiguity in the residue naming, specially regarding protonation states, that may lead to a wrong mapping. For example, SIRAH Tools always maps the residue name “HIS” to a Histidine protonated at Nε regardless the actual proton placement. Similarly, protonated Glutamic and Aspartic acid residues must be named “GLH” and “ASH”, otherwise they will be treated as negative charged residues. In addition, protonated and disulfide bonded Cysteines must be named “CYS” and “CYX” respectively. These kind of situations need to be carefully checked by the users. In all cases the residues preserve their identity when mapping and back-mapping the structures. Hence, the total charge of the protein should be the same at atomistic and SIRAH level. You can check the following mapping file to be sure of the compatibility: *sirah.amber/tools/CGCONV/maps/sirah_prot.map*.

Notice: By default charged termini are used, but it is possible to set then neutral by renaming the residues from **s**[*code*] to **a**[*code*] (Nt-acetylated) or **m**[*code*] (Ct-amidated) after mapping to CG, where [*code*] is the root residue name in SIRAH. For example, to set a neutral N-terminal Histidine protonated at Nε rename it from “sHe” to “aHe”.

Notice: This is the basic usage of the script *cgconv.pl*, you can learn other capabilities from its help:

```
./sirah.amber/tools/CGCONV/cgconv.pl -h
```

The input file *1CRN.pqr* contains all the heavy atoms composing the protein, while the output *1CRN_cg.pdb* preserves a few of them. Please check both PDB and PQR structures using VMD:

```
vmd -m sirah.amber/tutorial/5/1CRN.pqr 1CRN_cg.pdb
```

From now on it is just normal AMBER stuff!

2) Use a text editor to create the file *gensystem.leap* including the following lines:

```
# Load SIRAH force field
  addPath ./sirah.amber
  source leaprc.sirah

# Load model
  protein = loadpdb 1CRN_cg.pdb

# Info on system charge
  charge protein

# Set S-S bridges
  bond protein.3.BSG  protein.40.BSG
  bond protein.4.BSG  protein.32.BSG
  bond protein.16.BSG protein.26.BSG

# Add solvent, counterions and 0.15M NaCl
# Tuned solute-solvent closeness for best hydration
  solvateOct  protein WT4BOX 20 0.7
  addIonsRand protein NaW 22 ClW 22

# Save Parms
  saveAmberParmNetcdf protein 1CRN_cg.prmtop 1CRN_cg.ncrst

# EXIT
  quit
```

Notice: Each disulfide bond must be defined explicitly in LEAP using the command *bond*, e.g.: "*bond unit.ri.BSG unit.rj.BSG*". Where *ri* and *rj* correspond to the residue index in the topology file starting from 1, which may differ from the biological sequence in the PDB file. You can try the command *pdb4amber* to get those indexes from the atomistic structure, but be aware that it may not work if the Cysteine residues are too far away:

```
pdb4amber -i sirah.amber/tutorial/5/1CRN.pqr -o 1CRN_aa.pdb
cat 1CRN_aa_sslink
```

Notice: The available ionic species in SIRAH force field are: Na+ (NaW), K+ (KW) and Cl- (ClW). One ion pair (e.g. NaW-ClW) each 34 WT4 molecules renders a salt concentration of ~0.15M (see Appendix 1). If needed, we recommend adding counterions according to Machado et al. *SPLIT* [JCTC, **2020**].

3) Run the LEAP application to generate the molecular topology and initial coordinate files:

```
tleap -f gensystem.leap
```

Notice: Warning messages about long, triangular or square bonds in *leap.log* file are fine and expected due to the CG topology.

This should create a topology file *1CRN_cg.prmtop* and a coordinate file *1CRN_cg.ncrst*

Use VMD to check how the CG model looks like and particularly the presence of disulfide bonds:

```
vmd 1CRN_cg.prmtop 1CRN_cg.ncrst -e ./sirah.amber/tools/sirah_vmdtk.tcl
```

Notice: VMD assigns default radius to unknown atom types, the script *sirah_vmdtk.tcl* sets the right ones. It also provides a kit of useful selection macros, coloring methods, backmapping utility and a command to calculate and display the secondary structure of SIRAH proteins. Use the command *sirah_help* in the Tcl/Tk console of VMD to access the manual pages.

4) Run the simulation

Make a new folder for the run:

```
mkdir -p run; cd run
```

The folder *sirah.amber/tutorial/5/* contains typical input files for energy minimization (*em_WT4.in*), equilibration (*eq_WT4.in*) and production (*md_WT4.in*) runs. Please check carefully the input flags therein, in particular the definition of flag chngmask=0 at &ewald section is mandatory.

Energy Minimization of side chains and solvent by restraining the backbone:

```
sander -O\
 -i   ../sirah.amber/tutorial/5/em1_WT4.in\
 -p   ../1CRN_cg.prmtop\
 -c   ../1CRN_cg.ncrst\
 -ref ../1CRN_cg.ncrst\
 -o      1CRN_cg_em1.out\
 -r      1CRN_cg_em1.ncrst &
```

Energy Minimization of whole system:

```
sander -O\
 -i ../sirah.amber/tutorial/5/em2_WT4.in\
 -p ../1CRN_cg.prmtop\
 -c    1CRN_cg_em1.ncrst\
 -o    1CRN_cg_em2.out\
 -r    1CRN_cg_em2.ncrst &
```

Solvent Equilibration (NPT):

```
sander -O\
 -i   ../sirah.amber/tutorial/5/eq1_WT4.in\
 -p   ../1CRN_cg.prmtop\
 -c      1CRN_cg_em2.ncrst\
 -ref    1CRN_cg_em2.ncrst\
 -o      1CRN_cg_eq1.out\
 -r      1CRN_cg_eq1.ncrst\
 -x      1CRN_cg_eq1.nc &
```

Notice: Option *restraintmask=':1-46'* in input file *eq1_WT.in* must be set specifically for each system to embrace all protein's residues.

Soft equilibration to improve side chain solvation (NPT):

```
sander -O\
 -i   ../sirah.amber/tutorial/5/eq2_WT4.in\
 -p   ../1CRN_cg.prmtop\
 -c      1CRN_cg_eq1.ncrst\
 -ref    1CRN_cg_eq1.ncrst\
 -o      1CRN_cg_eq2.out\
 -r      1CRN_cg_eq2.ncrst\
 -x      1CRN_cg_eq2.nc &
```

Production (1000ns):

```
sander -O\
 -i ../sirah.amber/tutorial/5/md_WT4.in\
 -p ../1CRN_cg.prmtop\
 -c    1CRN_cg_eq2.ncrst\
 -o    1CRN_cg_md.out\
 -r    1CRN_cg_md.ncrst\
 -x    1CRN_cg_md.nc &
```

Notice: The same input files can be used to run on CPU or GPU versions of *pmemd*

That's it! Now you can analyze the trajectory.

Example of trajectory analysis

Process the output trajectory to account for the Periodic Boundary Conditions (PBC):

```
echo -e "autoimage\ngo\nquit\n" |
cpptraj\
 -p ../1CRN_cg.prmtop\
 -y 1CRN_cg_md.nc\
 -x 1CRN_cg_md_pbc.nc\
 --interactive
```

Visualize the secondary structure

Load the processed trajectory in VMD:

```
vmd ../1CRN_cg.prmtop ../1CRN_cg.ncrst 1CRN_cg_md_pbc.nc\
 -e ../sirah.amber/tools/sirah_vmdtk.tcl
```

At the Tk/Tcl console run the command *sirah_ss* to get the secondary structure of the CG protein.

Notice: After assigning the secondary structure it is possible to represent α-helices with Bendix in VMD 1.9.2 or upper by setting the backbone particle name to GC (do not check the CG box).

To analyze the output files from *sirah_ss*, go back at the shell command line and execute:

```
xmgrace -nxy ss_by_frame.xvg
xmgrace -nxy ss_by_res.xvg
```

The file ss.mtx can be processed to visualize the time evolution of the secondary structure by residue:

```
../sirah.amber/tools/ssmtx2png.R --mtx=ss.mtx
display ssmtx.png
```

The usage of *ssmtx2png.R* can be accessed through:

```
../sirah.amber/tools/ssmtx2png.R --help
```

Appendix 1: Calculating ionic concentrations

$\rho_{WT4} = \rho_{H2O} = 1000$ g/L
$MW_{H2O} = 18$ g/mol
1 WT4 ~ 11 H_2O

$$M=\frac{mol}{V} \quad ; \quad n=mol\,N_A \quad ; \quad \rho=\frac{m}{V} \quad ; \quad m=mol\,MW$$

$$V=\frac{m}{\rho}=\frac{mol\,MW_{H_2O}}{\rho}=\frac{n_{H_2O}MW_{H_2O}}{N_A\rho} \quad ; \quad M=\frac{mol}{V}=\frac{n_{ion}}{N_A V}=\frac{n_{ion}}{N_A}\frac{N_A\rho}{n_{H_2O}MW_{H_2O}}=\frac{n_{ion}1000}{n_{WT4}(11)(18)}\sim 5\frac{n_{ion}}{n_{WT4}}$$

Number of WT4 molecules per ion at 0.15M: $n_{WT4}=5\frac{n_{ion}}{M}=\frac{5(1)}{0.15}\sim 34$
