# Supplementary material for: A Practical Guide to All-Atom and Coarse-Grained Molecular Dynamics Simulations Using Amber and Gromacs: A Case Study of Disulfide-Bond Impact on the Intrinsically Disordered Amyloid Beta
Source: Int J Mol Sci. 2024 Jun 18;25(12):6698. doi: 10.3390/ijms25126698 (PMC11204378; doi:10.3390/ijms25126698)
Supplement: Supplementary file 1 [file ijms-25-06698-s001.zip › Scripts.zip!/Amber_all-atom_and_SIRAH/sirah_x2.2_20-08.amber/tutorial/Tutorial_6_sirah4amb.pdf]

Tutorial 6. SIRAH force field in AMBER

Simulation of coarse grained lipid bilayers in explicit solvent

By Exequiel Barrera
Mail any comment or suggestion to spantano@pasteur.edu.uy

This tutorial shows how to use the SIRAH force field to perform a coarse-grained (CG) simulation of a DMPC bilayer in explicit solvent (called WatFour, WT4) in four simple steps: 1 download; 2 map; 3 solvate and; 4 run. The main references for this tutorial are: Barrera et al. *SIRAH Lipids* [JCTC, **2019**, 15:5674], Machado et al. *SIRAH Tools* [Bioinformatics, **2017**, 32:1568]. We strongly advise you to read these articles before starting the tutorial.

Required Software
AMBER 16 and AMBER Tools 16 or later versions properly installed in your computer. The molecular visualization program VMD (freely available at *www.ks.uiuc.edu/Research/vmd*). The plotting software Grace (*http://plasma-gate.weizmann.ac.il/Grace*).

Prior knowledge
How to perform a standard atomistic molecular dynamic simulation with AMBER and basic usage of VMD. If you are not familiar with membrane stuff we strongly recommend you to first perform the AMBER tutorial on lipids (*http://ambermd.org/tutorials/advanced/tutorial16/*).

Hands on
0) Download the file *sirah_[version].amber.tgz* from *www.sirahff.com* and uncompress it into your working directory. Notice: *[version]* should be replaced with the actual package version e.g.: x2_18-09

```
tar -xzvf sirah_[version].amber.tgz
```

You will get a folder *sirah_[version].amber/* containing the force field definition, the SIRAH Tools in *sirah_[version].amber/tools/*, molecular structures to build up systems in *sirah_[version].amber/PDB/*, frequently asked questions in *sirah_[version].amber/tutorial/SIRAH_FAQs.pdf* and the required material to perform the tutorial in *sirah_[version].amber/tutorial/6/* .

Make a new folder for this tutorial in your working directory:

```
mkdir tutorial6; cd tutorial6
```

Create the following symbolic links in the folder *tutorial6*:

```
ln -s ../sirah_[version].amber sirah.amber
```

1) Map the atomistic structure of the preassembled DMPC bilayer to its CG representation:

```
./sirah.amber/tools/CGCONV/cgconv.pl\
 -i sirah.amber/tutorial/6/DMPC64.pdb\
 -o DMPC64_cg.pdb\
 -a sirah.amber/tools/CGCONV/maps/tieleman_lipid.map
```

Notice: By default no mapping is applied to lipids, as there is no standard naming convention for them. So users are requested to append a MAP file from the list in Table 1, by setting the flag *-a* in *cgconv.pl*. We recommend using PACKMOL (free at *http://m3g.iqm.unicamp.br/packmol*) for building the system. Reference building-block structures are provided at folder *sirah.amber/PDB/*, which agree with the mapping scheme in *sirah.amber/tools/CGCONV/maps/tieleman_lipid.map*. The provided DMPC bilayer contains 64 lipid molecules per leaflet distributed in a 6.4 * 6.4 nm surface, taking into account

an approximate area per lipid of 0.64 nm^2 at 333 K. The starting configuration was created with the input file *sirah.amber/tutorial/6/DMPC_bilayer.pkm.* See *SIRAH_FAQs.pdf* for cautions on mapping lipids to SIRAH and tips on using fragment-based topologies.

Notice: This is an advanced usage of the script *cgconv.pl*, you can learn more from its help:

```
./sirah.amber/tools/CGCONV/cgconv.pl -h
```

The input file *DMPC64.pdb* contains all the heavy atoms composing the lipids, while the output *DMPC64_cg.pdb* preserves a few of them. Please check both PDB structures using VMD:

```
vmd -m sirah.amber/tutorial/6/DMPC64.pdb DMPC64_cg.pdb
```

From now on it is just normal AMBER stuff!

2) Use a text editor to create the file *gensystem.leap* including the following lines:

```
# Load SIRAH force field
  addPath ./sirah.amber
  source leaprc.sirah

# Load model
  Lipid = loadpdb DMPC64_cg.pdb

# Add solvent, counterions and 0.15M NaCl
# Tuned solute-solvent closeness for best hydration
  solvateBox Lipid WT4BOX {0 0 40} 0.7
  addIonsRand Lipid NaW 33 ClW 33

# Save Parms
  saveAmberParmNetcdf Lipid DMPC64_cg.prmtop DMPC64_cg.ncrst

# EXIT
  quit
```

Notice: The available ionic species in SIRAH force field are: Na+ (NaW), K+ (KW) and Cl- (ClW). One ion pair (e.g. NaW-ClW) each 34 WT4 molecules renders a salt concentration of ~0.15M (see Appendix 1). If needed, we recommend adding counterions according to Machado et al. *SPLIT* [JCTC, **2020**].

3) Run the LEAP application to generate the molecular topology and initial coordinate files:

```
tleap -f gensystem.leap
```

Notice: Warning messages about long, triangular or square bonds in *leap.log* file are fine and expected due to the CG topology.

This should create a topology file *DMPC64_cg.prmtop* and a coordinate file *DMPC64_cg.ncrst*

Use VMD to check how the CG model looks like. By selecting +X, +Y and +Z periodic images from the *Periodic* tab in the *Graphical Representations* window you will see small vacuum slices at box boundaries. In the following step we will fix this issue by reducing the box dimensions a few angstroms. See *SIRAH_FAQs.pdf* for issues on membrane systems in AMBER.

```
vmd DMPC64_cg.prmtop DMPC64_cg.ncrst -e ./sirah.amber/tools/sirah_vmdtk.tcl
```

Notice: VMD assigns default radius to unknown atom types, the script *sirah_vmdtk.tcl* sets the right

ones. It also provides a kit of useful selection macros, coloring methods, backmapping utility and a command to calculate and display the secondary structure of SIRAH proteins. Use the command *sirah_help* in the Tcl/Tk console of VMD to access the manual pages.

4) Use a text editor to create the file *resize_box.cpptraj* including the following lines:

```
# New box dimensions
  box x 66 y 66 z 132

# Amber NetCDF Restart generation
  trajout DMPC64_cg_nb.ncrst

# Do it!
  go

# Exit
  quit
```

Notice: As PACKMOL does not consider periodicity while building up the system, increasing the XY box sides a few Angstroms may be required to avoid bad contacts between images.

5) Run the CPPTRAJ application to adjust the size of the simulation box.

```
cpptraj -p DMPC64_cg.prmtop -y DMPC64_cg.ncrst -i resize_box.cpptraj
```

Once again, use VMD to check the PBC images in the new box of the system.

```
vmd DMPC64_cg.prmtop DMPC64_cg_nb.ncrst -e ./sirah.amber/tools/sirah_vmdtk.tcl
```

6) Run the simulation

Make a new folder for the run:

```
mkdir -p run; cd run
```

The folder *sirah.amber/tutorial/6/* contains typical input files for energy minimization (*em_Lipid.in*), heating (*heat_Lipid.in*), equilibration (*eq_Lipid.in*) and production (*md_Lipid.in*) runs. Please check carefully the input flags.

Energy Minimization:

```
pmemd.cuda -O\
 -i   ../sirah.amber/tutorial/6/em_Lipid.in\
 -p   ../DMPC64_cg.prmtop\
 -c   ../DMPC64_cg_nb.ncrst\
 -o      DMPC64_cg_em.out\
 -r      DMPC64_cg_em.ncrst &
```

Heating:

```
pmemd.cuda -O\
 -i   ../sirah.amber/tutorial/6/heat_Lipid.in\
 -p   ../DMPC64_cg.prmtop\
 -c      DMPC64_cg_em.ncrst\
 -o      DMPC64_cg_eq_0.out\
 -r      DMPC64_cg_eq_0.ncrst\
 -x      DMPC64_cg_eq_0.nc &
```

Periodic box equilibration in GPU code (500 ps x 9):

```
for i in $(seq 1 9)
do
    echo "running equilibration $i"
    pmemd.cuda -O\
     -i ../sirah.amber/tutorial/6/eq_Lipid.in\
     -p ../DMPC64_cg.prmtop\
     -c    DMPC64_cg_eq_$(($i -1)).ncrst\
     -o    DMPC64_cg_eq_$i.out\
     -r    DMPC64_cg_eq_$i.ncrst\
     -x    DMPC64_cg_eq_$i.nc
done &
```

Notice: To avoid "*skinnb* errors" on GPU due to large box size fluctuations, the system must be equilibrated by several "short" runs using a large *skinnb* value. The number and length of the runs may vary according to the characteristic stabilization times of the system. For more information visit the Amber Lipid Force Field Tutorial at http://ambermd.org/tutorials/advanced/tutorial16/ .

Production (1000 ns)

```
pmemd.cuda -O\
 -i ../sirah.amber/tutorial/6/md_Lipid.in\
 -p ../DMPC64_cg.prmtop\
 -c    DMPC64_cg_eq_9.ncrst\
 -o    DMPC64_cg_md.out\
 -r    DMPC64_cg_md.ncrst\
 -x    DMPC64_cg_md.nc &
```

That's it! Now you can analyze the trajectory.

Example of trajectory analysis

Process the output trajectory to account for the Periodic Boundary Conditions (PBC):

```
echo -e "autoimage\ngo\nquit\n" |
cpptraj\
 -p ../DMPC64_cg.prmtop\
 -y    DMPC64_cg_md.nc\
 -x    DMPC64_cg_md_pbc.nc\
 --interactive
```

Now you can check the simulation using VMD:

```
vmd ../DMPC64_cg.prmtop DMPC64_cg_md_pbc.nc\
 -e ../sirah.amber/tools/sirah_vmdtk.tcl
```

Calculate the area per lipid

```
cpptraj -p ../DMPC64_cg.prmtop -i ../sirah.amber/tutorial/6/area_lipid.cpptraj
```

Use Grace to plot the results:

```
xmgrace apl_DMPC64_310K.dat
```

To calculate the area per lipid divides the membrane's area by the DMPC molecules per leaflet.

$$Area/Lipid = \frac{Box(x) * Box(y)}{64}$$

Density profiles and bilayer thickness

```
cpptraj -p ../DMPC64_cg.prmtop -i ../sirah.amber/tutorial/6/dens_profile.cpptraj
```

Use Grace to plot the results:

```
xmgrace -nxy dens_profile_DMPC64_310K.dat
```

The thickness of the bilayer is the distance between the two peaks corresponding to the position of phosphate beads (BFO) along the z-axis.

Mapping atomistic lipids to SIRAH

Table 1. Available mapping files (MAPs) at folder *sirah.amber/tools/CGCONV/maps/* for converting atomistic lipid structures to SIRAH models. Important! MAPs can not inter-convert different name conventions, e.g. *amber_lipid.map* won't generate fragment-based residues from residue-based force fields. Due to possible nomenclature conflicts, users are advised to check and modify the MAPs as required.

MAP	Type[1]	Compatibility	Source
amber_lipid.map	F	AMBER Lipid11-17 force fields	AMBER<br>HTMD
GAFF_lipid.map	R	AMBER GAFF force field	LipidBook
charmm_lipid.map	R	CHARMM 27/36 force field, and "CHARMM compatible" GAFF nomenclature	CHARMM-GUI<br>GROMACS<br>LipidBook<br>MemBuilder<br>HTMD<br>VMD
slipids.map	R	Stockholm lipids force field	SLIPIDS<br>MemBuilder
OPLSA-AA_2014_lipid.map	R	All-atoms lipids for OPLS force field	Maciejewski et al. 2014
OPLSA-UA_lipid.map	R	United-atom lipids for OPLS force field	LipidBook
GROMOS43a1_lipid.map	R	United-atom lipids for GROMOS 43a1 and CKP force fields	LipidBook<br>MemBuilder
GROMOS43a1-s3_lipid.map	R	United-atom lipids for GROMOS 43a1-s3 force field	GROMACS repo<br>LipidBook<br>MemBuilder
GROMOS53a6_lipid.map	R	United-atom lipids for GROMOS 53a6 force field	GROMACS repo<br>LipidBook<br>MemBuilder
tieleman_lipid.map	R	Berger lipids as implemented by Tieleman et al. for GROMOS force fields.	Tieleman<br>LipidBook

[1] Fragment-based (F) or Residue-based (R) topology.

Appendix 1: Calculating ionic concentrations

$\rho_{WT4} = \rho_{H2O}$ = 1000 g/L
MW_{H2O} = 18 g/mol
1 WT4 ~ 11 H_2O

$$M=\frac{mol}{V} \quad ; \quad n=mol\,N_A \quad ; \quad \rho=\frac{m}{V} \quad ; \quad m=mol\,MW$$

$$V=\frac{m}{\rho}=\frac{mol\,MW_{H_2O}}{\rho}=\frac{n_{H_2O}\,MW_{H_2O}}{N_A\rho} \quad ; \quad M=\frac{mol}{V}=\frac{n_{ion}}{N_A V}=\frac{n_{ion}}{N_A}\frac{N_A\rho}{n_{H_2O}MW_{H_2O}}=\frac{n_{ion}1000}{n_{WT4}(11)(18)}\sim 5\frac{n_{ion}}{n_{WT4}}$$

Number of WT4 molecules per ion at 0.15M: $n_{WT4}=5\frac{n_{ion}}{M}=\frac{5(1)}{0.15}\sim 34$
