# Supplementary material for: A Practical Guide to All-Atom and Coarse-Grained Molecular Dynamics Simulations Using Amber and Gromacs: A Case Study of Disulfide-Bond Impact on the Intrinsically Disordered Amyloid Beta
Source: Int J Mol Sci. 2024 Jun 18;25(12):6698. doi: 10.3390/ijms25126698 (PMC11204378; doi:10.3390/ijms25126698)
Supplement: Supplementary file 1 [file ijms-25-06698-s001.zip › Scripts.zip!/Amber_all-atom_and_SIRAH/sirah_x2.2_20-08.amber/tutorial/Tutorial_7_sirah4amb.pdf]

Tutorial 7. SIRAH force field in AMBER

Simulation of coarse grained membrane proteins in explicit solvent

By Exequiel Barrera
Mail any comment or suggestion to *spantano@pasteur.edu.uy*

This tutorial shows how to use the SIRAH force field to perform a coarse grained (CG) simulation of a protein embedded in a lipid bilayer using explicit solvent (called WatFour, WT4). The protocol consists of 5 simple steps: 1 download; 2 map; 3 insert the protein in the membrane; 4 solvate and; 5 run. The main references for this tutorial are: Machado et al. *SIRAH 2.0* [JCTC, **2019**, 15:2719], Barrera et al. *SIRAH Lipids* [JCTC, **2019**, 15:5674], Machado et al. *SIRAH Tools* [Bioinformatics, **2017**, 32:1568]. We strongly advise you to read these articles and complete Tutorials 5 and 6 before starting.

Required Software
AMBER 16 and AMBER Tools 16 or later versions properly installed in your computer. The molecular visualization program VMD (freely available at *www.ks.uiuc.edu/Research/vmd*). The plotting software Grace (*http://plasma-gate.weizmann.ac.il/Grace*).

Prior knowledge
How to perform a standard atomistic molecular dynamic simulation with AMBER and basic usage of VMD. Have performed Tutorials 5 and 6 of SIRAH in AMBER.

Hands on
0) Download the file *sirah_[version].amber.tgz* from *www.sirahff.com* and uncompress it into your working directory. Notice: *[version]* should be replaced with the actual package version e.g.: x2_18-09

```
tar -xzvf sirah_[version].amber.tgz
```

You will get a folder *sirah_[version].amber/* containing the force field definition, the SIRAH Tools in *sirah_[version].amber/tools/*, molecular structures to build up systems in *sirah_[version].amber/PDB/*, frequently asked questions in *sirah_[version].amber/tutorial/SIRAH_FAQs.pdf* and the required material to perform the tutorial in *sirah_[version].amber/tutorial/7/* .

Make a new folder for this tutorial in your working directory:

```
mkdir tutorial7; cd tutorial7
```

Create the following symbolic links in the folder *tutorial7*:

```
ln -s ../sirah_[version].amber sirah.amber
```

1) Map the protonated atomistic structure of protein 2KYV to its CG representation:

```
./sirah.amber/tools/CGCONV/cgconv.pl\
 -i sirah.amber/tutorial/7/2kyv.pqr\
 -o 2kyv_cg.pdb
```

Notice: The mapping to CG requires the correct protonation state of each residue at a given pH. See *SIRAH_FAQs.pdf* and Tutorial 5 for cautions while preparing and mapping atomistic proteins to SIRAH.

Notice: If you already have an atomistic protein within a membrane, then you can simply map the entire system to SIRAH (this is highly recommended) and skip the step 2, however clipping the

membrane patch may be required to set a correct solvation box (see bellow). By default no mapping is applied to lipids, as there is no standard naming convention for them. So users are requested to append a MAP file from the list in Table 1, by setting the flag *-a* in *cgconv.pl*. We recommend using PACKMOL (free at *http://m3g.iqm.unicamp.br/packmol*) for building the system. Reference building-block structures are provided at folder *sirah.amber/PDB/*, which agree with the mapping scheme *sirah.amber/tools/CGCONV/maps/tieleman_lipid.map*. See *SIRAH_FAQs.pdf* for cautions on mapping lipids to SIRAH and tips on using fragment-based topologies.

Notice: You can access the help of the script *cgconv.pl* by executing:

```
./sirah.amber/tools/CGCONV/cgconv.pl -h
```

2) Embed the protein in a lipid bilayer:
We will show one possible way to do it by starting from a pre-equilibrated CG membrane patch.

In *sirah.amber/tutorial/7/* you will find a pre-stabilized CG DMPC bilayer patch, concatenate it with the previously generated CG representation of the phospholamban (PLN) pentamer

```
head -qn -1 2kyv_cg.pdb ./sirah.amber/tutorial/7/DMPC_cg.pdb\
 > 2kyv_DMPC_cg_init.pdb
```

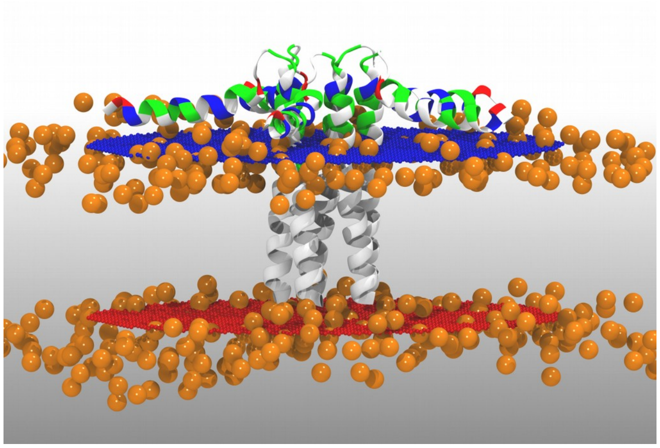

Luckily, we already oriented the protein inside the membrane. For setting up your own system you can go to Orientations of Proteins in Membranes (OPM) database (*http://opm.phar.umich.edu*) and (if your structure is available) use the dummy atoms provided there to make them match with your membrane model (see right image).

Using VMD to delete lipid molecules in close contact with the protein:

For a proper treatment and visualization of the system in VMD you must first generate the molecular topology and initial coordinate files:

3) Use a text editor to create the file *geninit.leap* including the following lines:

```
# Load SIRAH force field
  addPath ./sirah.amber
  source leaprc.sirah

# Load model
  ProtMem = loadpdb 2kyv_DMPC_cg_init.pdb

# Save Parms
  saveAmberParmNetcdf ProtMem 2kyv_DMPC_cg_init.prmtop 2kyv_DMPC_cg_init.ncrst

# EXIT
  quit
```

4) Run the LEAP application to generate the molecular topology and initial coordinate files:

```
tleap -f geninit.leap
```

Notice: Warning messages about long, triangular or square bonds in *leap.log* file are fine and expected due to the CG topology.

Open the files on VMD:

```
vmd 2kyv_DMPC_cg_init.prmtop 2kyv_DMPC_cg_init.ncrst\
 -e sirah.amber/tools/sirah_vmdtk.tcl
```

Notice: VMD assigns default radius to unknown atom types, the script *sirah_vmdtk.tcl* sets the right ones. It also provides a kit of useful selection macros, coloring methods, backmapping utility and a command to calculate and display the secondary structure of SIRAH proteins. Use the command *sirah_help* in the Tcl/Tk console of VMD to access the manual pages.

In the VMD main window, select *Graphics* > *Representations*. In the *Selected Atoms* text entry type:

```
not (same residue as (sirah_membrane within 3.5 of sirah_protein) or
(sirah_membrane and x < 5 or x > 142 or y < 2 or y > 140))
```

Notice: In the first part of the selection, lipid molecules in close contact with the protein are removed. The second one is made to “trim” the membrane patch, deleting lipids with acyl chains located in the periodic boundary images. This is frequent when using pre-equilibrated membrane patches and is necessary to avoid clashes in the following steps.

Save the refined protein-membrane system: In the VMD main window click on `2kyv_DMPC_cg_init.prmtop`, then select *File* > *Save Coordinates.* In the Selected atoms option choose the selection you have just created and *Save* as `2kyv_DMPC_cg.pdb`.

From now on it is just normal AMBER stuff!

5) Use a text editor to create the file *gensystem.leap* including the following lines:

```
# Load SIRAH force field
  addPath ./sirah.amber
  source leaprc.sirah

# Load model
  ProtMem = loadpdb 2kyv_DMPC_cg.pdb

# Info on system charge
  charge ProtMem

# Prevent adding solvent molecules beyond the membrane boundaries
  setbox ProtMem centers 0

# Add solvent, counterions and 0.15M NaCl
# Tuned solute-solvent closeness for best hydration
  solvateBox  ProtMem WT4BOX {0,0,27} 0.7
  addIonsRand ProtMem NaW 109 ClW 124

# Save Parms
  saveAmberParmNetcdf ProtMem 2kyv_DMPC_cg.prmtop 2kyv_DMPC_cg.ncrst

# EXIT
  quit
```

Notice: The available ionic species in SIRAH force field are: Na+ (NaW), K+ (KW) and Cl- (ClW). One ion pair (e.g. NaW-ClW) each 34 WT4 molecules renders a salt concentration of ~0.15M (see Appendix 1). Counterions were added according to Machado et al. *SPLIT* [JCTC, **2020**].

6) Run the LEAP application to generate the molecular topology and initial coordinate files:

```
tleap -f gensystem.leap
```

This should create a topology file 2kyv_DMPC_cg.*prmtop* and a coordinate file 2kyv_DMPC_cg.*ncrst*

Use VMD to check how the CG model looks like. By selecting +X, +Y and +Z periodic images from the *Periodic* tab in the *Graphical Representations* window you will see unwanted water near the hydrophobic region of the membrane and small vacuum slices at box boundaries. In the following step we will fix these issues by deleting those water molecules and reducing the box dimensions a few angstroms. See *SIRAH_FAQs.pdf* for issues on membrane systems in AMBER.

```
vmd 2kyv_DMPC_cg.prmtop 2kyv_DMPC_cg.ncrst -e ./sirah.amber/tools/sirah_vmdtk.tcl
```

7) Use a text editor to create the file *resize_box.cpptraj* including the following lines:

```
# Set reference coordinate file
  reference 2kyv_DMPC_cg.ncrst

# Remove water using distance-based masks
  strip :WT4&(@BCT1,BCT2,BC13,BC23<:12.0) parmout 2kyv_DMPC_cg.prmtop

# New box dimensions
  box x 128 y 128 z 114

# Amber NetCDF Restart generation
  trajout 2kyv_DMPC_cg_nb.ncrst

# Do it!
  go

# Exit
  quit
```

Notice: This is a critical step when preparing membrane systems to simulate with AMBER. In this case, the new box dimensions were set after some trial and error tests to allow for limited overlap between periodic box images. An excessive overlap may lead to important atom clashes an eventual system explosion during minimization/simulation, while insufficient overlap may impact the membrane cohesivity at PBC boundaries leading to pore formations or other issues.

8) Run the CPPTRAJ application to adjust the size of the simulation box.

```
cpptraj -p 2kyv_DMPC_cg.prmtop -y 2kyv_DMPC_cg.ncrst -i resize_box.cpptraj
```

Once again, use VMD to check the PBC images in the new box of the system.

```
vmd 2kyv_DMPC_cg.prmtop 2kyv_DMPC_cg_nb.ncrst\
 -e ./sirah.amber/tools/sirah_vmdtk.tcl
```

9) Run the simulation

Make a new folder for the run:

```
mkdir -p run; cd run
```

The folder *sirah.amber/tutorial/7/* contains typical input files for energy minimization (*em_Prot-Lip.in*), heating (*heat_Prot-Lip.in*), equilibration (*eq_Prot-Lip.in*) and production (*md_Prot-Lip.in*) runs. Please check carefully the input flags.

Energy Minimization of side chains by restraining the backbone:

```
pmemd.cuda -O\
 -i   ../sirah.amber/tutorial/7/em1_Prot-Lip.in\
 -p   ../2kyv_DMPC_cg.prmtop\
 -c   ../2kyv_DMPC_cg_nb.ncrst\
 -ref ../2kyv_DMPC_cg_nb.ncrst\
 -o      2kyv_DMPC_cg_em_1.out\
 -r      2kyv_DMPC_cg_em_1.ncrst &
```

Energy Minimization of the whole system:

```
pmemd.cuda -O\
 -i   ../sirah.amber/tutorial/7/em2_Prot-Lip.in\
 -p   ../2kyv_DMPC_cg.prmtop\
 -c      2kyv_DMPC_cg_em_1.ncrst\
 -ref    2kyv_DMPC_cg_em_1.ncrst\
 -o      2kyv_DMPC_cg_em_2.out\
 -r      2kyv_DMPC_cg_em_2.ncrst &
```

Heating:

```
pmemd.cuda -O\
 -i   ../sirah.amber/tutorial/7/heat_Prot-Lip.in\
 -p   ../2kyv_DMPC_cg.prmtop\
 -c      2kyv_DMPC_cg_em_2.ncrst\
 -ref    2kyv_DMPC_cg_em_2.ncrst\
 -o      2kyv_DMPC_cg_eq_0.out\
 -r      2kyv_DMPC_cg_eq_0.ncrst\
 -x      2kyv_DMPC_cg_eq_0.nc &
```

Periodic box equilibration in GPU code (500 ps x 9):

```
for i in $(seq 1 9)
do
    echo "running equilibration $i"
    pmemd.cuda -O\
     -i ../sirah.amber/tutorial/7/eq_Prot-Lip.in\
     -p ../2kyv_DMPC_cg.prmtop\
     -c    2kyv_DMPC_cg_eq_$(($i -1)).ncrst\
     -ref  2kyv_DMPC_cg_eq_$(($i -1)).ncrst\
     -o    2kyv_DMPC_cg_eq_$i.out\
     -r    2kyv_DMPC_cg_eq_$i.ncrst\
     -x    2kyv_DMPC_cg_eq_$i.nc
done &
```

Notice: To avoid “*skinnb* errors” on GPU due to large box size fluctuations, the system must be equilibrated by several “short” runs using a large *skinnb* value. The number and length of the runs may vary according to the characteristic stabilization times of the system. For more information visit the Amber Lipid Force Field Tutorial at http://ambermd.org/tutorials/advanced/tutorial16/ .

Production (1000 ns)

```
pmemd.cuda -O\
 -i ../sirah.amber/tutorial/7/md_Prot-Lip.in\
 -p ../2kyv_DMPC_cg.prmtop\
 -c    2kyv_DMPC_cg_eq_9.ncrst\
 -o    2kyv_DMPC_cg_md.out\
 -r    2kyv_DMPC_cg_md.ncrst\
 -x    2kyv_DMPC_cg_md.nc &
```

Notice: The same input files can be used to run on *sander* and the CPU version of *pmemd*

That’s it! Now you can analyze the trajectory.

Example of trajectory analysis

Process the output trajectory to account for the Periodic Boundary Conditions (PBC):

```
echo -e "autoimage\ngo\nquit\n" |
cpptraj\
 -p ../2kyv_DMPC_cg.prmtop\
 -y    2kyv_DMPC_cg_md.nc\
 -x    2kyv_DMPC_cg_md_pbc.nc\
 --interactive
```

Now you can check the simulation using VMD:

```
vmd ../2kyv_DMPC_cg.prmtop\
 2kyv_DMPC_cg_md_pbc.nc -e ../sirah.amber/tools/sirah_vmdtk.tcl
```

Mapping atomistic lipids to SIRAH

Table 1. Available mapping files (MAPs) at folder *sirah.amber/tools/CGCONV/maps/* for converting atomistic lipid structures to SIRAH models. Important! MAPs can not inter-convert different name conventions, e.g. *amber_lipid.map* won't generate fragment-based residues from residue-based force fields. Due to possible nomenclature conflicts, users are advised to check and modify the MAPs as required.

MAP	Topol[1]	Compatibility	Source
amber_lipid.map	F	AMBER Lipid11-17 force fields	AMBER<br>HTMD
GAFF_lipid.map	R	AMBER GAFF force field	LipidBook
charmm_lipid.map	R	CHARMM 27/36 force field, and "CHARMM compatible" GAFF nomenclature	CHARMM-GUI<br>GROMACS<br>LipidBook<br>MemBuilder<br>HTMD<br>VMD
slipids.map	R	Stockholm lipids force field	SLIPIDS<br>MemBuilder
OPLSA-AA_2014_lipid.map	R	All-atoms lipids for OPLS force field	Maciejewski et al. 2014
OPLSA-UA_lipid.map	R	United-atom lipids for OPLS force field	LipidBook
GROMOS43a1_lipid.map	R	United-atom lipids for GROMOS 43a1 and CKP force fields	LipidBook<br>MemBuilder
GROMOS43a1-s3_lipid.map	R	United-atom lipids for GROMOS 43a1-s3 force field	GROMACS repo<br>LipidBook<br>MemBuilder
GROMOS53a6_lipid.map	R	United-atom lipids for GROMOS 53a6 force field	GROMACS repo<br>LipidBook<br>MemBuilder
tieleman_lipid.map	R	Berger lipids as implemented by Tieleman et al. for GROMOS force fields.	Tieleman<br>LipidBook

[1] Fragment-based (F) or Residue-based (R) topology.

Appendix 1: Calculating ionic concentrations

$\rho_{WT4} = \rho_{H2O}$ = 1000 g/L
MW_{H2O} = 18 g/mol
1 WT4 ~ 11 H_2O

$$M=\frac{mol}{V} \quad ; \quad n=mol\,N_A \quad ; \quad \rho=\frac{m}{V} \quad ; \quad m=mol\,MW$$

$$V=\frac{m}{\rho}=\frac{mol\,MW_{H_2O}}{\rho}=\frac{n_{H_2O}MW_{H_2O}}{N_A\rho} \quad ; \quad M=\frac{mol}{V}=\frac{n_{ion}}{N_A V}=\frac{n_{ion}}{N_A}\frac{N_A\rho}{n_{H_2O}MW_{H_2O}}=\frac{n_{ion}1000}{n_{WT4}(11)(18)}\sim 5\frac{n_{ion}}{n_{WT4}}$$

Number of WT4 molecules per ion at 0.15M: $n_{WT4}=5\frac{n_{ion}}{M}=\frac{5(1)}{0.15}\sim 34$
