# Supplementary material for: A Practical Guide to All-Atom and Coarse-Grained Molecular Dynamics Simulations Using Amber and Gromacs: A Case Study of Disulfide-Bond Impact on the Intrinsically Disordered Amyloid Beta
Source: Int J Mol Sci. 2024 Jun 18;25(12):6698. doi: 10.3390/ijms25126698 (PMC11204378; doi:10.3390/ijms25126698)
Supplement: Supplementary file 1 [file ijms-25-06698-s001.zip › Scripts.zip!/Gromacs_Martini/ABeta/production/backmapping/charmm36.ff/forcefield.doc]

CHARMM all-atom force field

**********************************************************************************

* GROMACS port of the CHARMM force field written by Andras Wacha	*

* based on the original work of: *

* E. Prabhu Raman, Justin A. Lemkul, Robert Best and Alexander D. MacKerell, Jr. *

*	*

* Conversion script available at: https://gitlab.com/awacha/charmm2gmx	*

* On-line documentation	: https://awacha.gitlab.io/charmm2gmx	*

*	*

* Version 0.7.dev45+g7b82040.d20221208 of the charmm2gmx utility was used	*

*	*

* Questions, bug reports etc: https://gitlab.com/awacha/charmm2gmx/issues	*

**********************************************************************************

Parameters taken from CHARMM36 and CGenFF 4.6

Parameters and topologies are included from the following files:

================================================================

aminoacids:

-----------

- toppar_c36_jul22/par_all36m_prot.prm

- toppar_c36_jul22/top_all36_prot.rtf

- toppar_c36_jul22/stream/prot/toppar_all36_prot_heme.str

- toppar_c36_jul22/stream/prot/toppar_all36_prot_c36m_d_aminoacids.str

- toppar_c36_jul22/stream/prot/toppar_all36_prot_modify_res.str

- toppar_c36_jul22/stream/prot/toppar_all36_prot_retinol.str

- toppar_c36_jul22/stream/prot/toppar_all36_prot_arg0.str

- toppar_c36_jul22/stream/prot/toppar_all36_prot_fluoro_alkanes.str

- toppar_c36_jul22/stream/prot/toppar_all36_prot_na_combined.str

- toppar_c36_jul22/stream/prot/toppar_all36_prot_model.str

lipid:

------

- toppar_c36_jul22/par_all36_lipid.prm

- toppar_c36_jul22/top_all36_lipid.rtf

- toppar_c36_jul22/stream/lipid/toppar_all36_lipid_bacterial.str

- toppar_c36_jul22/stream/lipid/toppar_all36_lipid_cardiolipin.str

- toppar_c36_jul22/stream/lipid/toppar_all36_lipid_cholesterol.str

- toppar_c36_jul22/stream/lipid/toppar_all36_lipid_detergent.str

- toppar_c36_jul22/stream/lipid/toppar_all36_lipid_ether.str

- toppar_c36_jul22/stream/lipid/toppar_all36_lipid_inositol.str

- toppar_c36_jul22/stream/lipid/toppar_all36_lipid_lps.str

- toppar_c36_jul22/stream/lipid/toppar_all36_lipid_miscellaneous.str

- toppar_c36_jul22/stream/lipid/toppar_all36_lipid_prot.str

- toppar_c36_jul22/stream/lipid/toppar_all36_lipid_sphingo.str

- toppar_c36_jul22/stream/lipid/toppar_all36_lipid_yeast.str

silicates:

----------

- toppar_c36_jul22/silicates/toppar/top_silicates.inp

- toppar_c36_jul22/silicates/toppar/par_silicates.inp

metals:

-------

- toppar_c36_jul22/metals/CHARMM_METAL/top_all22_prot_metals.inp

- toppar_c36_jul22/metals/CHARMM_METAL/par_all22_prot_metals.inp

cgenff:

-------

- toppar_c36_jul22/par_all36_cgenff.prm

- toppar_c36_jul22/top_all36_cgenff.rtf

solvent:

--------

- toppar_c36_jul22/toppar_water_ions.str

- toppar_c36_jul22/stream/misc/toppar_ions_won.str

na:

---

- toppar_c36_jul22/par_all36_na.prm

- toppar_c36_jul22/top_all36_na.rtf

- toppar_c36_jul22/stream/na/toppar_all36_na_modifications.str

- toppar_c36_jul22/stream/na/toppar_all36_na_nad_ppi.str

carb:

-----

- toppar_c36_jul22/par_all36_carb.prm

- toppar_c36_jul22/top_all36_carb.rtf

- toppar_c36_jul22/stream/carb/toppar_all36_carb_glycolipid.str

- toppar_c36_jul22/stream/carb/toppar_all36_carb_glycopeptide.str

- toppar_c36_jul22/stream/carb/toppar_all36_carb_imlab.str

- toppar_c36_jul22/stream/carb/toppar_all36_carb_lignin.str

ethers:

-------

- toppar_c36_jul22/par_all35_ethers.prm

- toppar_c36_jul22/top_all35_ethers.rtf

References:

==========

aminoacids:

-----------

- Best, Robert B. and Zhu, Xiao and Shim, Jihyun and Lopes, Pedro E. M. and Mittal, Jeetain and Feig, Michael and MacKerell, Alexander D.: Optimization of the Additive CHARMM All-Atom Protein Force Field Targeting Improved Sampling of the Backbone {$\phi$}, {$\psi$} and Side-Chain Χ1 and Χ2 Dihedral Angles. J. Chem. Theory Comput. (2012) 8. 9 pp 3257-3273 (DOI: 10.1021/ct300400x)

- MacKerell, Alexander D. and Feig, Michael and Brooks, Charles L.: Improved Treatment of the Protein Backbone in Empirical Force Fields. J. Am. Chem. Soc. (2004) 126. 3 pp 698-699 (DOI: 10.1021/ja036959e)

- Huang, Jing and Rauscher, Sarah and Nawrocki, Grzegorz and Ran, Ting and Feig, Michael and {de Groot}, Bert L and Grubm{\"u}ller, Helmut and MacKerell, Alexander D: CHARMM36m: An Improved Force Field for Folded and Intrinsically Disordered Proteins. Nature Methods (2016) 0. pp - (DOI: 10.1038/nmeth.4067)

- MacKerell, A. D. and Bashford, D. and Bellott, M. and Dunbrack, R. L. and Evanseck, J. D. and Field, M. J. and Fischer, S. and Gao, J. and Guo, H. and Ha, S. and {Joseph-McCarthy}, D. and Kuchnir, L. and Kuczera, K. and Lau, F. T. K. and Mattos, C. and Michnick, S. and Ngo, T. and Nguyen, D. T. and Prodhom, B. and Reiher, W. E. and Roux, B. and Schlenkrich, M. and Smith, J. C. and Stote, R. and Straub, J. and Watanabe, M. and {Wi{\'o}rkiewicz-Kuczera}, J. and Yin, D. and Karplus, M.: All-Atom Empirical Potential for Molecular Modeling and Dynamics Studies of Proteins. J. Phys. Chem. B (1998) 102. 18 pp 3586-3616 (DOI: 10.1021/jp973084f)

Fluoro alkanes:

Chen, I Jen and Yin, Daxu and MacKerell Jr., Alexander D.: Combined Ab Initio/Empirical Approach for Optimization of Lennard-Jones Parameters for Polar-Neutral Compounds. Journal of Computational Chemistry (2002) 23. 2 pp 199-213 (DOI: 10.1002/jcc.1166)

Neutral arginine:

Li, Libo and Vorobyov, Igor and MacKerell, Alexander D. and Allen, Toby W.: Is Arginine Charged in a Membrane?. Biophysical Journal (2008) 94. 2 pp L11-L13 (DOI: 10.1529/biophysj.107.121566)

Phosphotyrosine:

Feng, Ming-Hsiang and Philippopoulos, Marios and MacKerell, Alexander D. and Lim, Carmay: Structural Characterization of the Phosphotyrosine Binding Region of a High-Affinity SH2 Domain-Phosphopeptide Complex by Molecular Dynamics Simulation and Chemical Shift Calculations. J. Am. Chem. Soc. (1996) 118. 45 pp 11265-11277 (DOI: 10.1021/ja961530r)

lipid:

------

- Leonard, Alison N. and Pastor, Richard W. and Klauda, Jeffery B.: Parameterization of the CHARMM All-Atom Force Field for Ether Lipids and Model Linear Ethers. J. Phys. Chem. B (2018) 122. 26 pp 6744-6754 (DOI: 10.1021/acs.jpcb.8b02743)

- Klauda, Jeffery B. and Venable, Richard M. and Freites, J. Alfredo and O'Connor, Joseph W. and Tobias, Douglas J. and {Mondragon-Ramirez}, Carlos and Vorobyov, Igor and MacKerell, Alexander D. and Pastor, Richard W.: Update of the CHARMM All-Atom Additive Force Field for Lipids: Validation on Six Lipid Types. The Journal of Physical Chemistry B (2010) 114. 23 pp 7830-7843 (DOI: 10.1021/jp101759q)

- Klauda, Jeffery B. and Monje, Viviana and Kim, Taehoon and Im, Wonpil: Improving the CHARMM Force Field for Polyunsaturated Fatty Acid Chains. J. Phys. Chem. B (2012) 116. 31 pp 9424-9431 (DOI: 10.1021/jp304056p)

Branched lipids:

Lim, Joseph B. and Klauda, Jeffery B.: Lipid Chain Branching at the Iso- and Anteiso-Positions in Complex Chlamydia Membranes: A Molecular Dynamics Study. Biochimica et Biophysica Acta (BBA) - Biomembranes (2011) 1808. 1 pp 323-331 (DOI: 10.1016/j.bbamem.2010.07.036)

Ceramide:

"Submitted by Sam Tonddast-Navaei of U. Cincinnati. Advised by Russell Devane"

Cyclic-replacement of double bond:

Pandit, Kunal R. and Klauda, Jeffery B.: Membrane Models of E. Coli Containing Cyclic Moieties in the Aliphatic Lipid Chain. Biochimica et Biophysica Acta (BBA) - Biomembranes (2012) 1818. 5 pp 1205-1210 (DOI: 10.1016/j.bbamem.2012.01.009)

Original model:

Pitman, Michael C. and Suits, Frank and MacKerell, Alexander D. and Feller, Scott E.: Molecular-Level Organization of Saturated and Polyunsaturated Fatty Acids in a Phosphatidylcholine Bilayer Containing Cholesterol. Biochemistry (2004) 43. 49 pp 15318-15328 (DOI: 10.1021/bi048231w)

PSM and SSM lipids:

Venable, Richard~M. and Sodt, Alexander~J. and Rogaski, Brent and Rui, Huan and Hatcher, Elizabeth and MacKerell, Alexander~D. and Pastor, Richard~W. and Klauda, Jeffery~B.: CHARMM All-Atom Additive Force Field for Sphingomyelin: Elucidation of Hydrogen Bonding and of Positive Curvature. Biophysical Journal (2014) 107. 1 pp 134-145 (DOI: 10.1016/j.bpj.2014.05.034)

Updated model:

Lim, Joseph B. and Rogaski, Brent and Klauda, Jeffery B.: Update of the Cholesterol Force Field Parameters in CHARMM. J. Phys. Chem. B (2012) 116. 1 pp 203-210 (DOI: 10.1021/jp207925m)

silicates:

----------

- Lopes, Pedro E. M. and Murashov, Vladimir and Tazi, Mouhsine and Demchuk, Eugene and MacKerell, Alexander D.: Development of an Empirical Force Field for Silica. Application to the Quartz-Water Interface. J. Phys. Chem. B (2006) 110. 6 pp 2782-2792 (DOI: 10.1021/jp055341j)

metals:

-------

- Feng, Jie and Slocik, Joseph M. and Sarikaya, Mehmet and Naik, Rajesh R. and Farmer, Barry L. and Heinz, Hendrik: Influence of the Shape of Nanostructured Metal Surfaces on Adsorption of Single Peptide Molecules in Aqueous Solution. Small (2012) 8. 7 pp 1049-1059 (DOI: 10.1002/smll.201102066)

- Heinz, Hendrik and Vaia, R. A. and Farmer, B. L. and Naik, R. R.: Accurate Simulation of Surfaces and Interfaces of Face-Centered Cubic Metals Using 12-6 and 9-6 Lennard-Jones Potentials. J. Phys. Chem. C (2008) 112. 44 pp 17281-17290 (DOI: 10.1021/jp801931d)

- Heinz, Hendrik and Farmer, Barry L. and Pandey, Ras B. and Slocik, Joseph M. and Patnaik, Soumya S. and Pachter, Ruth and Naik, Rajesh R.: Nature of Molecular Interactions of Peptides with Gold, Palladium, and Pd-Au Bimetal Surfaces in Aqueous Solution. J. Am. Chem. Soc. (2009) 131. 28 pp 9704-9714 (DOI: 10.1021/ja900531f)

- Coppage, Ryan and Slocik, Joseph M. and Briggs, Beverly D. and Frenkel, Anatoly I. and Heinz, Hendrik and Naik, Rajesh R. and Knecht, Marc R.: Crystallographic Recognition Controls Peptide Binding for Bio-Based Nanomaterials. J. Am. Chem. Soc. (2011) 133. 32 pp 12346-12349 (DOI: 10.1021/ja203726n)

- Feng, Jie and B.~Pandey, Ras and J.~Berry, Rajiv and L.~Farmer, Barry and R.~Naik, Rajesh and Heinz, Hendrik: Adsorption Mechanism of Single Amino Acid and Surfactant Molecules to Au {111} Surfaces in Aqueous Solution: Design Rules for Metal-Binding Molecules. Soft Matter (2011) 7. 5 pp 2113-2120 (DOI: 10.1039/C0SM01118E)

cgenff:

-------

- Yu, Wenbo and He, Xibing and Vanommeslaeghe, Kenno and MacKerell, Alexander D.: Extension of the CHARMM General Force Field to Sulfonyl-Containing Compounds and Its Utility in Biomolecular Simulations. Journal of Computational Chemistry (2012) 33. 31 pp 2451-2468 (DOI: 10.1002/jcc.23067)

- Vanommeslaeghe, K. and Hatcher, E. and Acharya, C. and Kundu, S. and Zhong, S. and Shim, J. and Darian, E. and Guvench, O. and Lopes, P. and Vorobyov, I. and Mackerell, A. D.: CHARMM General Force Field: A Force Field for Drug-like Molecules Compatible with the CHARMM All-Atom Additive Biological Force Fields. Journal of Computational Chemistry (2010) 31. 4 pp 671-690 (DOI: 10.1002/jcc.21367)

solvent:

--------

Ions by Roux et. al.:

Beglov, Dmitrii and Roux, Beno{\^i}t: Finite Representation of an Infinite Bulk System: Solvent Boundary Potential for Computer Simulations. J. Chem. Phys. (1994) 100. 12 pp 9050-9063 (DOI: 10.1063/1.466711)

Ions by Youngdo Won:

Won, Youngdo: Force Field for Monovalent, Divalent, and Trivalent Cations Developed under the Solvent Boundary Potential. J. Phys. Chem. A (2012) 116. 47 pp 11763-11767 (DOI: 10.1021/jp309150r)

TIP3P water:

Jorgensen, William L. and Chandrasekhar, Jayaraman and Madura, Jeffry D. and Impey, Roger W. and Klein, Michael L.: Comparison of Simple Potential Functions for Simulating Liquid Water. J. Chem. Phys. (1983) 79. 2 pp 926-935 (DOI: 10.1063/1.445869)

Zinc ion:

Stote, Roland H. and Karplus, Martin: Zinc Binding in Proteins and Solution: A Simple but Accurate Nonbonded Representation. Proteins: Structure, Function, and Bioinformatics (1995) 23. 1 pp 12-31 (DOI: 10.1002/prot.340230104)

na:

---

- Denning, Elizabeth J. and Priyakumar, U. Deva and Nilsson, Lennart and Mackerell, Alexander D.: Impact of 2'-Hydroxyl Sampling on the Conformational Properties of RNA: Update of the CHARMM All-Atom Additive Force Field for RNA. Journal of Computational Chemistry (2011) 32. 9 pp 1929-1943 (DOI: 10.1002/jcc.21777)

- Hart, Katarina and Foloppe, Nicolas and Baker, Christopher M. and Denning, Elizabeth J. and Nilsson, Lennart and MacKerell, Alexander D.: Optimization of the CHARMM Additive Force Field for DNA: Improved Treatment of the BI/BII Conformational Equilibrium. J. Chem. Theory Comput. (2012) 8. 1 pp 348-362 (DOI: 10.1021/ct200723y)

- Foloppe, Nicolas and Jr, Alexander D. MacKerell: All-Atom Empirical Force Field for Nucleic Acids: I. Parameter Optimization Based on Small Molecule and Condensed Phase Macromolecular Target Data. Journal of Computational Chemistry (2000) 21. 2 pp 86-104 (DOI: 10.1002/(SICI)1096-987X(20000130)21:2<86::AID-JCC2>3.0.CO;2-G)

- MacKerell, Alexander D. and Banavali, Nilesh K.: All-Atom Empirical Force Field for Nucleic Acids: II. Application to Molecular Dynamics Simulations of DNA and RNA in Solution. Journal of Computational Chemistry (2000) 21. 2 pp 105-120 (DOI: 10.1002/(SICI)1096-987X(20000130)21:2<105::AID-JCC3>3.0.CO;2-P)

Carbocyclic sugar parameters:

Wang, Peiyuan and Brank, Adam S. and Banavali, Nilesh K. and Nicklaus, Marc C. and Marquez, Victor E. and Christman, Judith K. and MacKerell, Alexander D.: Use of Oligodeoxyribonucleotides with Conformationally Constrained Abasic Sugar Targets To Probe the Mechanism of Base Flipping by HhaI DNA (Cytosine C5)-Methyltransferase. J. Am. Chem. Soc. (2000) 122. 50 pp 12422-12434 (DOI: 10.1021/ja001989s)

Locked nucleic acids:

Hartono, Yossa Dwi and Xu, You and Karshikoff, Andrey and Nilsson, Lennart and Villa, Alessandra: Modeling pK Shift in DNA Triplexes Containing Locked Nucleic Acids. J. Chem. Inf. Model. (2018) 58. 4 pp 773-783 (DOI: 10.1021/acs.jcim.7b00741)

NAD+, NADH, ADP, ATP, PPI and related analogs:

Pavelites, Joseph J. and Gao, Jiali and Bash, Paul A. and Mackerell, Alexander D.: A Molecular Mechanics Force Field for NAD+ NADH, and the Pyrophosphate Groups of Nucleotides. Journal of Computational Chemistry (1997) 18. 2 pp 221-239 (DOI: 10.1002/(SICI)1096-987X(19970130)18:2<221::AID-JCC7>3.0.CO;2-X)

Peptide nucleic acids:

Jasi{\'n}ski, Maciej and Feig, Michael and Trylska, Joanna: Improved Force Fields for Peptide Nucleic Acids with Optimized Backbone Torsion Parameters. J. Chem. Theory Comput. (2018) 14. 7 pp 3603-3620 (DOI: 10.1021/acs.jctc.8b00291)

carb:

-----

- Vermaas, Josh V. and Dellon, Lauren D. and Broadbelt, Linda J. and Beckham, Gregg T. and Crowley, Michael F.: Automated Transformation of Lignin Topologies into Atomic Structures with LigninBuilder. ACS Sustainable Chem. Eng. (2019) 7. 3 pp 3443-3453 (DOI: 10.1021/acssuschemeng.8b05665)

- Kern, Nathan~R. and Lee, Hui~Sun and Wu, Emilia~L. and Park, Soohyung and Vanommeslaeghe, Kenno and MacKerell, Alexander~D. and Klauda, Jeffery~B. and Jo, Sunhwan and Im, Wonpil: Lipid-Linked Oligosaccharides in Membranes Sample Conformations That Facilitate Binding to Oligosaccharyltransferase. Biophysical Journal (2014) 107. 8 pp 1885-1895 (DOI: 10.1016/j.bpj.2014.09.007)

- V.~Vermaas, Josh and Petridis, Loukas and Ralph, John and F.~Crowley, Michael and T.~Beckham, Gregg: Systematic Parameterization of Lignin for the CHARMM Force Field. Green Chemistry (2019) 21. 1 pp 109-122 (DOI: 10.1039/C8GC03209B)

O-glycan linkages:

Mallajosyula, Sairam S. and MacKerell, Alexander D.: Influence of Solvent and Intramolecular Hydrogen Bonding on the Conformational Properties of O-Linked Glycopeptides. J. Phys. Chem. B (2011) 115. 38 pp 11215-11229 (DOI: 10.1021/jp203695t)

Phosphates and sulfates:

Mallajosyula, Sairam S. and Guvench, Olgun and Hatcher, Elizabeth and MacKerell, Alexander D.: CHARMM Additive All-Atom Force Field for Phosphate and Sulfate Linked to Carbohydrates. J. Chem. Theory Comput. (2012) 8. 2 pp 759-776 (DOI: 10.1021/ct200792v)

carbohydrate derivatives and glycosidic linkages for glycoproteins:

Guvench, Olgun and Mallajosyula, Sairam S. and Raman, E. Prabhu and Hatcher, Elizabeth and Vanommeslaeghe, Kenno and Foster, Theresa J. and Jamison, Francis W. and MacKerell, Alexander D.: CHARMM Additive All-Atom Force Field for Carbohydrate Derivatives and Its Utility in Polysaccharide and Carbohydrate–Protein Modeling. J. Chem. Theory Comput. (2011) 7. 10 pp 3162-3180 (DOI: 10.1021/ct200328p)

furanose monosaccharides:

Hatcher, Elizabeth and Guvench, Olgun and MacKerell, Alexander D.: CHARMM Additive All-Atom Force Field for Aldopentofuranoses, Methyl-aldopentofuranosides, and Fructofuranose. J. Phys. Chem. B (2009) 113. 37 pp 12466-12476 (DOI: 10.1021/jp905496e)

glycosidic linkages involving furanoses:

Raman, E. Prabhu and Guvench, Olgun and MacKerell, Alexander D.: CHARMM Additive All-Atom Force Field for Glycosidic Linkages in Carbohydrates Involving Furanoses. J. Phys. Chem. B (2010) 114. 40 pp 12981-12994 (DOI: 10.1021/jp105758h)

hexopyranose glycosidic linkages:

Guvench, Olgun and Hatcher, Elizabeth and Venable, Richard M. and Pastor, Richard W. and MacKerell, Alexander D.: CHARMM Additive All-Atom Force Field for Glycosidic Linkages between Hexopyranoses. J. Chem. Theory Comput. (2009) 5. 9 pp 2353-2370 (DOI: 10.1021/ct900242e)

linear sugars, sugar alcohols, and inositol:

Hatcher, Elizabeth R. and Guvench, Olgun and MacKerell, Alexander D.: CHARMM Additive All-Atom Force Field for Acyclic Polyalcohols, Acyclic Carbohydrates, and Inositol. J. Chem. Theory Comput. (2009) 5. 5 pp 1315-1327 (DOI: 10.1021/ct9000608)

pyranose monosaccharides:

Guvench, Olgun and Greene, Shannon N. and Kamath, Ganesh and Brady, John W. and Venable, Richard M. and Pastor, Richard W. and Mackerell, Alexander D.: Additive Empirical Force Field for Hexopyranose Monosaccharides. Journal of Computational Chemistry (2008) 29. 15 pp 2543-2564 (DOI: 10.1002/jcc.21004)

ethers:

-------

- Vorobyov, Igor and Anisimov, Victor M. and Greene, Shannon and Venable, Richard M. and Moser, Adam and Pastor, Richard W. and MacKerell, Alexander D.: Additive and Classical Drude Polarizable Force Fields for Linear and Cyclic Ethers. J. Chem. Theory Comput. (2007) 3. 3 pp 1120-1133 (DOI: 10.1021/ct600350s)

- Lee, Hwankyu and Venable, Richard M. and MacKerell, Alexander D. and Pastor, Richard W.: Molecular Dynamics Studies of Polyethylene Oxide and Polyethylene Glycol: Hydrodynamic Radius and Shape Anisotropy. Biophysical Journal (2008) 95. 4 pp 1590-1599 (DOI: 10.1529/biophysj.108.133025)
