# Supplementary material for: A Practical Guide to All-Atom and Coarse-Grained Molecular Dynamics Simulations Using Amber and Gromacs: A Case Study of Disulfide-Bond Impact on the Intrinsically Disordered Amyloid Beta
Source: Int J Mol Sci. 2024 Jun 18;25(12):6698. doi: 10.3390/ijms25126698 (PMC11204378; doi:10.3390/ijms25126698)
Supplement: Supplementary file 1 [file ijms-25-06698-s001.zip › Scripts.zip!/Gromacs_SIRAH/sirah.ff/forcefield.doc]

SIRAH force field 2.2 [Jul 2019]

**********************************************************

* SIRAH force field © | version July 2019

*

**********************************************************
